# Target-Directed Dynamic Combinatorial Chemistry Affords Binders of *Mycobacterium tuberculosis* IspE

**DOI:** 10.1021/acsomega.4c05537

**Published:** 2024-08-29

**Authors:** Maria Braun-Cornejo, Camilla Ornago, Vidhisha Sonawane, Jörg Haupenthal, Andreas M. Kany, Eleonora Diamanti, Gwenaëlle Jézéquel, Norbert Reiling, Wulf Blankenfeldt, Peter Maas, Anna K. H. Hirsch

**Affiliations:** † Specs Compound Handling, B.V., Bleiswijkseweg 55, 2712 PB Zoetermeer, The Netherlands; ‡ Saarland University, Department of Pharmacy, Campus Building E8.1, 66123 Saarbrücken, Germany; § Helmholtz Institute for Pharmaceutical Research Saarland (HIPS) − Helmholtz Centre for Infection Research (HZI), Campus Building E8.1, 66123 Saarbrücken, Germany; ∥ Department Structure and Function of Proteins Helmholtz Centre for Infection Research Inhoffenstrasse 7, 38124 Braunschweig, Germany; ⊥ RG Microbial Interface Biology, Research Center Borstel Leibniz Lung Center, Parkallee 1-40, 23845 Borstel, Germany; # German Center for Infection Research (DZIF), Partner Site Hamburg-Lübeck-Borstel-Riems, 23845 Borstel, Germany

## Abstract

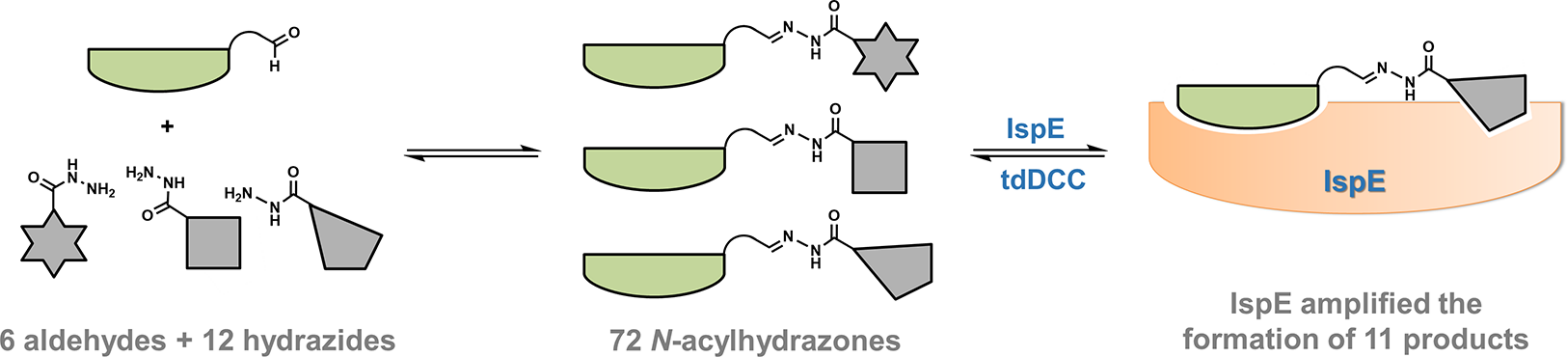

In the search for new antitubercular compounds, we leveraged target-directed dynamic combinatorial chemistry (tdDCC) as an efficient hit-identification method. In tdDCC, the target selects its own binders from a dynamic library generated *in situ*, reducing the number of compounds that require synthesis and evaluation. We combined a total of 12 hydrazides and six aldehydes to generate 72 structurally diverse *N*-acylhydrazones. To amplify the best binders, we employed anti-infective target 4-diphosphocytidyl-2*C*-methyl-d-erythritol kinase (IspE) from *Mycobacterium tuberculosis* (*Mtb*). We successfully validated the use of tdDCC as hit-identification method for IspE and optimized the analysis of tdDCC hit determination. From the 72 possible *N*-acylhydrazones, we synthesized 12 of them, revealing several new starting points for the development of IspE inhibitors as antibacterial agents.

## Introduction

Target-directed dynamic combinatorial chemistry (tdDCC) has evolved into an effective hit-identification method for medicinal chemistry. A set of “building blocks” with complementary reactivities engage in a reversible bond-forming reaction, resulting in a dynamic combinatorial library (DCL) that comprises all possible building block combinations.^1^ A target protein can influence this equilibrium by stabilizing binders, resulting in their amplification. This selective amplification circumvents the need to synthesize the entire library, making tdDCC particularly efficient in the time- and cost-sensitive early stages of drug discovery.^2−4^ Recent literature highlights the application of new targets, analytical methods, and reaction types for tdDCC, resulting in robust protocols and an increasing number of success stories.^5−8^ One of the frequently used reactions in tdDCC is the formation of acylhydrazones from aldehydes and hydrazides. In 2021, we applied this reaction to 1-deoxy-d-xylulose-5-phosphate synthase (DXPS, or DXS), the first enzyme of the 2*C*-methyl-d-erythritol 4-phosphate (MEP) pathway obtaining promising new anti-infective compounds.^6^

While all enzymes of the MEP-pathway are considered potential antibiotic drug targets, few inhibitors have been reported since its discovery in the 1990s.^9−11^ This pathway is vital for critical pathogens, such as *Mycobacterium tuberculosis* (*Mtb*) and Gram-negative bacteria to afford the essential isoprenoid precursors isopentenyl diphosphate (IDP) and dimethylallyl diphosphate (DMADP).^12^ Moreover, it is absent in mammals, where the mevalonate pathway yields the same building blocks, making target-related toxicity less of a concern.^9,13^ The identification of new inhibitors against the fourth enzyme of the pathway, 4-diphosphocytidyl-2*C*-methyl-d-erythritol kinase (IspE), is challenging. Most available crystal structures of IspE homologs are cocrystals with the natural substrates. So far, only one IspE structure in complex with an inhibitor has been published, likely due to its structural similarity to the natural IspE substrate and to its high potency (Figure S1).^14,15^ With single-digit micromolar activity, this compound is the most potent known IspE inhibitor, but it lacks whole-cell activity. A few more IspE inhibitors have been discovered, however, activities are moderate and no significant improvements in whole-cell activity have been achieved.^16^ Recent efforts to discover IspE hits have included structure-based virtual screening, however, only a very limited number of hits could be identified. In particular, we focused on filtering the screening library for compounds with properties that aid accumulation in *Escherichia coli* (*Ec*). The applied filter seemed successful, but optimization of the affinity for *Ec*IspE proved to be difficult.^17^ Most IspE studies use protein homologs from model organisms like *E. coli* and *Aquifex aeolicus*. Very recently, Choi *et al.* took a step toward the identification of antitubercular IspE inhibitors by screening 15 million compounds *in silico* against *Mtb*IspE (PDB: 3PYG). On this basis, one hit was confirmed, but could not be optimized thus far.^18^ To the author’s knowledge, no other inhibitors targeting *Mtb*IspE have been reported. Therefore, we highlight the need for additional efforts toward investigating IspE from critical pathogen homologs like *M. tuberculosis*.

We explored a new avenue in IspE hit-discovery by assessing the compatibility of *Mtb*IspE with tdDCC. This targeted approach allows to explore novel scaffolds without relying on knowledge of protein structure or pre-existing inhibitors. One of the limitations of tdDCC is the need of a substantial amount of protein, which has to be stable under the reaction conditions. Therefore, more robust proteins originating from model organism are often used in place of pathogenic proteins. Recently, we optimized the production of *Mtb*IspE (Supporting Information) and could verify its stability under the conditions necessary to build an acylhydrazone DCL (Figure S2).^6^ Therefore, we are confident that tdDCC can be a valuable method to obtain new specific starting points for MEP-pathway inhibitors using the pathogen homolog *Mtb*IspE.

## Results and Discussion

### Design of Dynamic Combinatorial Libraries

In this study, we used *Mtb*IspE as target protein and the reversible reaction of hydrazides and aldehydes to form dynamic combinatorial libraries (DCLs) of acylhydrazones. This reaction proved to be successful using DXPS, another MEP pathway enzyme, which is why we applied the same approach with IspE. To accelerate the reaction at neutral pH, aniline was used as nucleophilic catalyst activating the aldehydes.^2,4,19^ We analyzed the protein’s effect on the DCLs by comparing HPLC-MS/MS data of blank vs protein-templated (PT) experiments. The experiments spanned 3 days, with periodic sampling (every 2 h, followed by two samples per day after 8 h). The addition of NaOH to raise the pH and halt the reactions ensured the sample composition was preserved for analysis.^19^ Templated experiments were conducted in duplicate, and the protein was denatured and removed prior to HPLC-MS/MS analysis.

**Figure 1:**
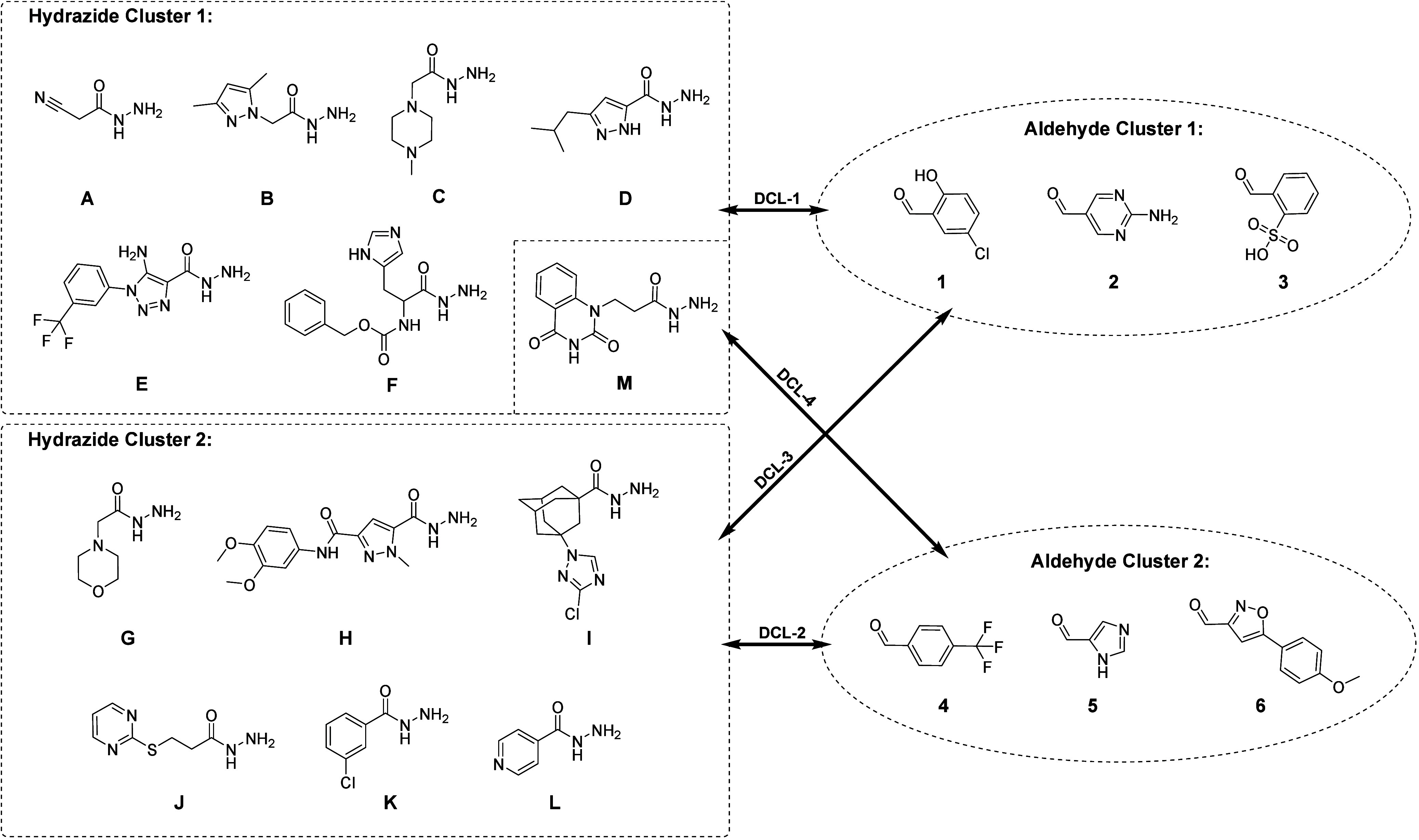
Clusters of hydrazides (**A**–**M**) and aldehydes (**1**–**6**) building blocks used in four dynamic combinatorial libraries (DCLs). Note: In DCL-1 hydrazides **A**–**F** were used, and in DCL-4, hydrazide **A** was exchanged for **M**.

By employing a randomized tdDCC hit-identification approach, we covered a wide chemical space, not relying on any structural information on the protein or its ligands. We selected diverse commercially available aldehyde and hydrazide building blocks with various geometries, sizes, and functional groups, including (hetero)aromatic rings (six- and five-membered, electron-rich/-poor), aliphatic moieties (morpholine, piperazine, adamantane), and various types of spacers. To ensure the HPLC-MS/MS analysis would be feasible, not too cluttered, nor contain isobaric products, we designed four distinct DCLs, each comprising 18 acylhydrazones. The building blocks were organized into two clusters of hydrazides (**A**–**M**) with six building blocks each and two clusters of aldehydes (**1**–**6**) with three building blocks each. To cover all possible combinations, we reacted aldehydes from cluster 1 with hydrazides from cluster 1 in DCL-1, aldehydes from cluster 2 with hydrazides from cluster 2 in DCL-2, aldehydes from cluster 1 with hydrazides from cluster 2 in DCL-3, and aldehydes from cluster 2 with hydrazides from cluster 1 in DCL-4, theoretically leading to 72 products (Figure 1).

### Determination of tdDCC IspE Hits

**Figure 2:**
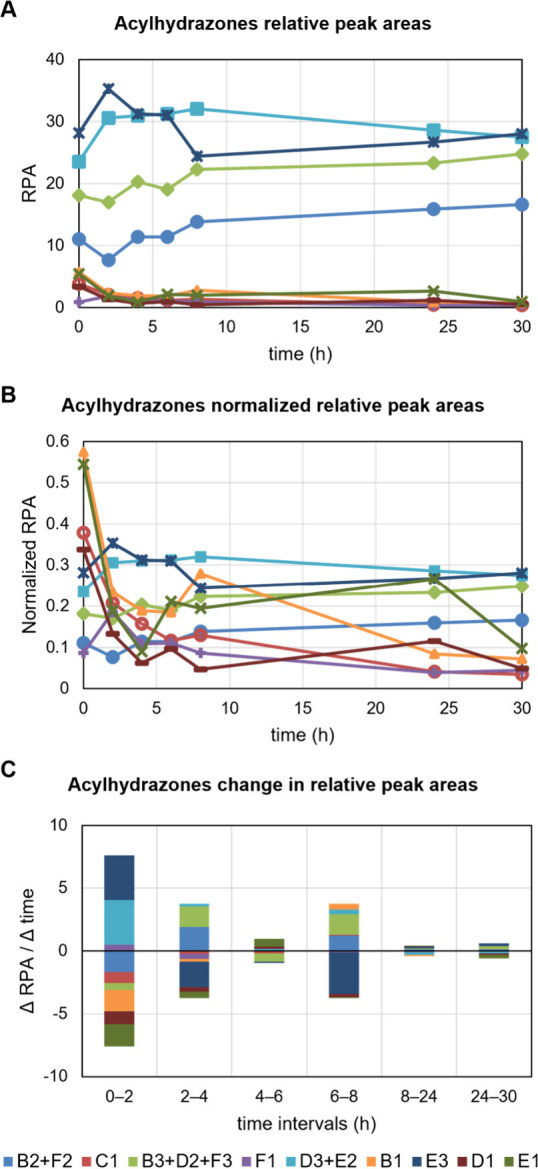
Graphical determination of equilibrium in blank DCL-1. A) Evolution of relative peak areas (RPA) of formed acylhydrazones over time. B) Normalized RPAs over time. C) Change of RPA in each time interval.

**Figure 3:**
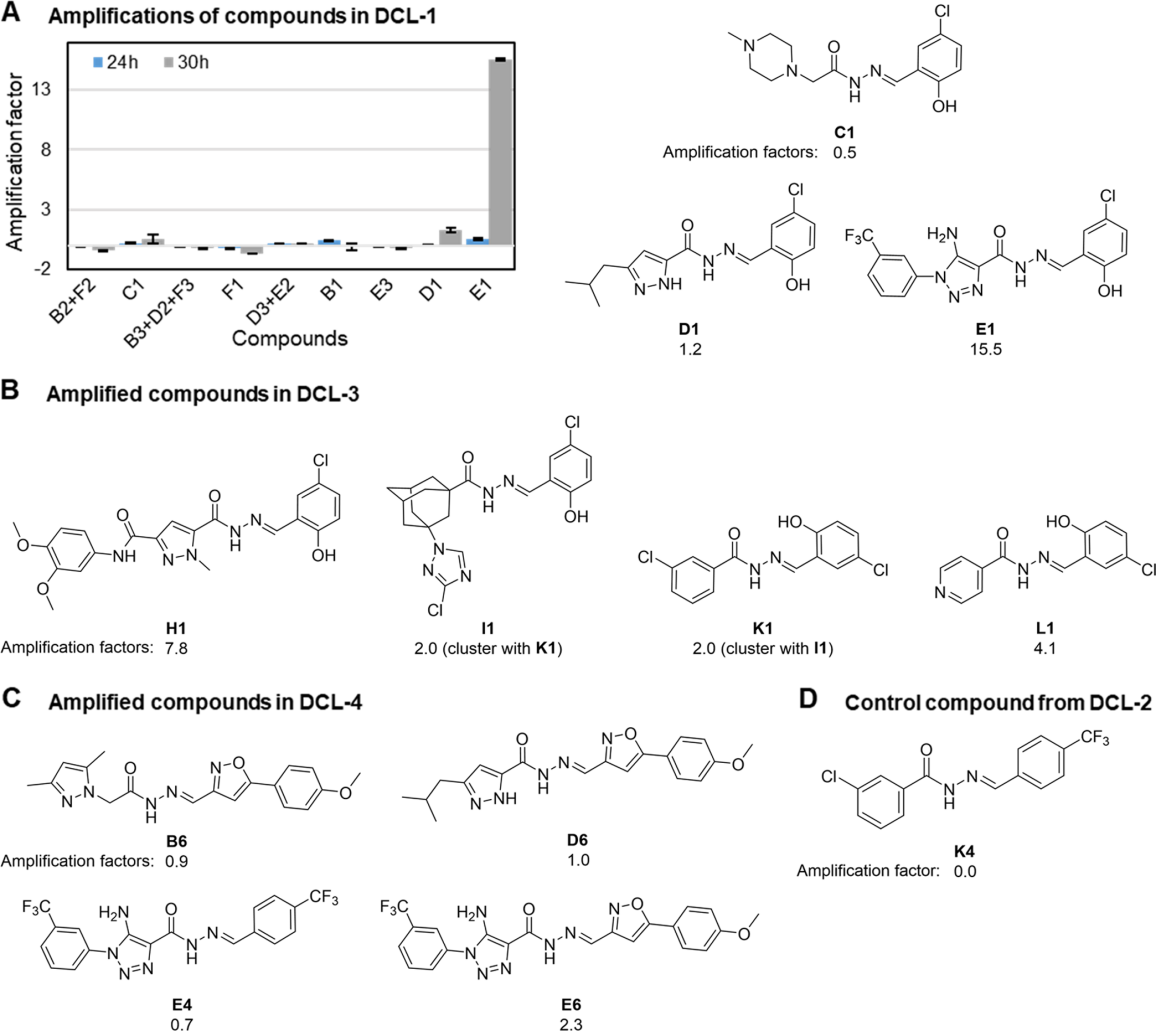
Structures of selected compounds for biological evaluation. A) Amplification of acylhydrazones in the protein-templated experiment of dynamic combinatorial library-1 (DCL-1) at 24 and 30 h, and the structures of selected preliminary hits. B) Amplified compounds in DCL-3. C) Amplified compounds in DCL-4. D) Selected control compound from DCL-2.

The tdDCC hits and thus potential *Mtb*IspE binders are amplified in the PT experiment compared to the blank experiment. The first step in the analysis of DCLs is the determination of the equilibrium since the amplification factors need to be calculated when the concentration of products is stable. To determine the evolution of the possible products, it is important to measure the characteristic UV absorbance of the acylhydrazones linker (310 nm) during the HPLC-MS/MS run. The resulting UV peaks are assigned to each product using the MS data (Figures S3–S6). The sum of all peak areas is normalized to 100% in order to obtain the respective relative peak area (RPA) for each product. The RPA values represent relative concentrations, determined at each time point of the blank experiment until the values stabilize. Plotting the RPAs over time shows the evolution of the products: in DCL-1 the four curves of the most abundant compounds are visibly flattening from 8 h onward (Figure 2A). However, the rest of the compounds are out of range, making a visual inspection difficult. Given that we do not want to neglect the importance of low-abundant compounds on the equilibrium, we normalized the RPA values using decimal scaling, confirming that the library starts stabilizing from 8 h onward (Figure 2B). To determine when the slope reaches zero, we calculated the change in RPA for each time interval and represented it in a stacked bar chart (Figure 2C), showing that the compounds’ concentration was most stable in the time interval 8–24 h and remained largely stable thereafter. The other three libraries behaved in the same way, reaching equilibrium within 24 h and also maintained stable concentrations beyond (Figures S7–S9). To determine the tdDCC hits, we compared the RPAs of the PT experiments to the blanks at 24 h and set a threshold of at least 50% increase in compound formation. In total, 11 acylhydrazones passed our criteria, with amplification factors ranging from 0.5 to 15.5, qualifying them for resynthesis and further evaluation (Figure 3). During the analysis of chromatograms, challenges arose concerning product identification. Some compound peaks were not neatly separated, forcing us to consider them in clusters, others presented two peaks, likely due to isomers, and were considered separately in our evaluation. Lastly, some compounds were detected by MS, but their respective UV signal interfered with the injection peak or was too low for integration (<1%), both in the blank and PT experiments. Consequently, we excluded these compounds from the analysis (Figure S3–S6).

In DCL-1 at 24 h, one potential hit, **E1**, reported a small amplification factor of 0.5 (Figure 3A). The protein concentration used for the PT experiments was rather low with 25 mol % *Mtb*IspE, a higher concentration should intensify amplification factors, therefore we decided to use 40 mol % from DCL-2 onward.^6^ Nevertheless, we decided to also determine the product amplifications at 30 h for DCL-1 since the equilibrium remained stable (Figure 2). Interestingly, at 30 h, the amplification of **E1** grew extremely, and two more products, **C1** and **D1**, passed our amplification threshold, indicating that the PT experiment’s equilibrium is not in line with the blank experiment (Figure 3A). It is noteworthy that no product containing hydrazide **A** could be identified in the chromatograms of DCL-1. We believe that **A**’s methylene is rather acidic due to the strongly electron-withdrawing nitrile and carbonyl groups. When adding NaOH for sample preparation, compounds containing **A** were likely deprotonated, followed by degradation or formation of undesired byproducts.

In the case of DCL-2, despite employing a higher protein concentration compared to DCL-1, there was no significant amplification of any product at 24 h or the subsequent time point, 35 h. The product amplifications remained largely unchanged in both time points, indicating that the PT experiments also had reached equilibrium. We selected compound **K4** as a control since our protein seemingly did not have any influence on its formation (Figure S10).

DCL-3 yielded four potential hits at 24 h, **L1**, **H1**, **I1** and **K1**, however, the latter two products were clustered together due to their significant peak overlap (Figure S5). Regarding **H1**, a complication arose as it appeared as a single peak in one PT duplicate, while in the other PT sample and the blank, it appeared as two overlapping peaks (Figure S5). This discrepancy may be due to **H1** existing as different isomers. However, the lone peak was insufficient to compensate for the missing area of the two peaks in the duplicate, resulting in a significant error in **H1**’s RPA. Despite the differences in RPA, both PT duplicates showcased substantial amplification compared to the blank, with amplification factors of 11.4 and 4.2, firmly establishing **H1** as a tdDCC hit (Figure 3B and Figure S5).

Given the reactivity issue for hydrazide **A** in DCL-1, we opted for its substitution in cluster 1 with a structurally very different hydrazide, **M** (Figure 1). One product, **M5**, could not be detected despite these modifications. Nevertheless, we identified four potential hits, namely, **B6**, **D6**, **E4** and **E6**, each exhibiting relatively modest amplification factors ranging from 0.7 to 2.3 at 24 h (Figure 3C).

To validate these 11 preliminary hits and assess their biological relevance, we synthesized them in a one-step reaction. The corresponding hydrazides and aldehydes were refluxed in methanol overnight, affording the acylhydrazones in moderate to excellent yields (Supporting Information). From a literature search, we found that compound **L1** was previously identified to have antifungal properties.^20^

#### Structure–Amplification Relationship of tdDCC Hits

Regarding the composition of the tdDCC hits (Figure 3), there is one building block that clearly stood out, aldehyde **1**. It is found in seven of the 11 potential hits, notably, the most amplified compounds (**E1**, **H1**, **L1**) all incorporate aldehyde **1**. This observation suggests that the arrangement of the aromatic scaffold with a *para*-substituted OH and Cl fit and interacted well in an IspE pocket. In addition, the structure of aldehyde **6** may also be interesting, since it is present in three potential hits **B6**, **D6** and **E6**. Aldehydes **2**, **3** and **5** are not included in any tdDCC hit. The most amplified aldehydes **1** and **6** both contain an electron-rich phenyl ring due to electron-donating substituents hydroxy and methoxy, which both can also be hydrogen-bond acceptors. In contrast, the aldehydes that were not amplified do all contain comparatively electron-deficient aromatic rings. The most prevalent hydrazide component found in the hits is **E**, which appears in **E1**, **E4** and **E6**. Hydrazide **E** contains an electron-deficient phenyl ring that is linked to an electron-rich heterocycle. Hydrazides **F**, **G**, **J** and **M** are not included in any tdDCC hit, possibly **F** and **M** are too bulky, and **J** is too electron-deficient. The limited presence of amplified compounds containing **C**, **G** and **I** indicates that aliphatic groups are not well-tolerated by IspE. Interestingly, the by far most amplified hit, **E1**, contains the two most prevalent building blocks of their kind, suggesting that their structural and electronic features align with complementary sites of the enzyme. These motifs along with their hydrogen-bond donor/acceptor geometry could be promising starting points for the development of new *Mtb*IspE inhibitors. It cannot be ruled out that the acylhydrazone linker may also contribute to favorable interactions, especially hydrogen bonds, and should be kept in mind.

#### *In Silico* Elucidation of tdDCC Hits’ Binding Mode

Docking simulations provided valuable insights that further rationalize the structure–amplification relationship within the active site of *Mtb*IspE. We generated ten binding poses for each compound across the four DCLs to analyze trends in the binding modes of the compounds. The defined binding site for the docking encompassed both natural substrates’ binding pockets as defined in the published crystal structures of *Mtb*IspE (3PYE for CDP-ME and 3PYG for ADP).^21^ Importantly, we did not set any binding restrain to minimize bias, and we removed poses that were unrealistic due to bad torsion, or inter- and intramolecular clashes. The analysis of these compound poses revealed preferred binding tendencies for two of the three most common motifs in the tdDCC hits (fragment **1** and **E)**, whereas fragment **6** did not show a particular binding tendency. Aldehyde **1** is by far the most frequently appearing fragment in the hits’ structures and the docking results showed two pockets where the characteristic phenol moiety in **1** primarily bound. One pocket was the substrate binding site of CDP-ME, where the phenol bound slightly deeper within the pocket compared to the substrate’s cytidine moiety (Figure S11). The other pocket was an unoccupied area adjacent to the ADP binding site. The poses in the CDP-ME binding pocket showed a clear orientation directing the hydroxy substituent toward amino acid Ser182 with distances of 2.6–3 Å suggesting the possibility of an H-bond. Interestingly, these poses belonged to hit compounds, whereas the poses adjacent to the ADP binding site belonged to both protein-amplified and not amplified compounds, and did not present such a clear orientation. Similarly, the hits containing hydrazide **E**, had the trifluoromethyl-substituted phenyl positioned deep inside the CDP-ME binding site (Figure S12). In the case of **E1**, containing both of the fragments with the same binding tendency, motif **1** bound to a third unoccupied and very narrow pocket. This unique binding mode was the same for all poses of **E1** (Figure S13) and gives a possible explanation for its exceptionally high amplification compared to the rest of the hits. Lastly, these findings suggest a link between the CDP-ME binding site and enhanced amplification.

#### Acylhydrazones’ Low Solubility Hinders Biological Evaluation

Prior to biological evaluation of the tdDCC hits, we tested their solubility in a standard buffer as well as in *M. tuberculosis* 7H9 growth medium. Our results revealed a major issue, most compounds’ solubility lies between 20 and 30 μM in PBS with 5% DMSO, representing a challenge for our established hit-verification methods. Merely product **C1** reaches a good solubility close to 1 mM, **L1** and **B6** show moderate solubilities. Regrettably, with 3.3 μM the least soluble compound is **E1**, making our most amplified and promising compound unsuitable for biological evaluation. We assessed the solubility in *Mtb* growth medium given that it is a complex, nutrient-rich environment with a distinct solubility profile. However, the trend continued, only seven hits are soluble at 8 μM or above, the remaining five compounds, including **E1**, were not eligible for antitubercular activity testing (Table 1).

**Table 1:** Solubility Determination and Biological Evaluation of tdDCC Hits on *Mycobacterium tuberculosis*, HepG2, *Escherichia coli* and IspE

DCL	cmp	solubility 5% DMSO in PBS (μM)	solubility in 7H9 (μM)	*Mtb*IspE binding at 20 μM	*Mtb*IspE Δ*T*_m_ at 20 μM (°C)	*Mtb*H37Rv MIC_90_ (μM)	*Ec*TolC %inh. At 50 μM	*EcK12* %inh. At 50 μM	HepG2 CC_50_ (μM)
**1**	**C1**	>800	16	Yes	<0.3	no inh. at max. sol.	17.1 ± 0.1	<10	>50
	**D1**	22 ± 9	insoluble	No	<0.3	n.d.	28 ± 11	11 ± 4	7.9 ± 0.8
	**E1**	3.3 ± 1	insoluble	n.d.^a^	n.d.	n.d.	<10	n.d.	>50
**2**	**K4**	21 ± 6	8	1/3	+0.7	no inh. at max. sol.	38 ± 4	12 ± 4	>50
**3**	**H1**	28 ± 6	32	No	<0.3	no inh. at max. sol.	<10	n.d.	32 ± 5
	**I1**	8.0 ± 4	64	n.d.	n.d.	no inh. at max. sol.	33 ± 3	<10	n.d.
	**K1**	25 ± 4	insoluble	Yes	+0.7	n.d.	39 ± 8	<10	7 ± 3
	**L1**	108 ± 3	16	No	<0.3	no inh. at max. sol.	52 ± 3	18 ± 6	∼50
**4**	**B6**	65 ± 35	16	2/3	<0.3	no inh. at max. sol.	<10	n.d.	>50
	**D6**	20 ± 10	16	No	<0.3	no inh. at max. sol.	39 ± 6	<10	>50
	**E4**	12 ± 6	insoluble	Yes	n.d.	n.d.	<10	n.d.	>50
	**E6**	29 ± 10	insoluble	No	+0.7	n.d.	<10	n.d.	>50

a n.d.: not determined.

#### Evaluation of tdDCC Hits *Mtb*IspE Binding

The compounds’ low solubility prevents the determination of binding affinities (*K*_D_). Nevertheless, qualitative binding assessments were conducted for all compounds, except **E1** and **I1**, using MicroScale Thermophoresis (MST) at 20 μM in triplicate. **C1**, **K1**, and **E4** consistently demonstrated binding across all measurements. Compound **B6** exhibited inconsistent binding, as it bound in two out of three measurements, while our negative control, **K4**, showed binding in only one measurement. These two compounds’ irreproducible binding may be due to nonspecific interactions. Given that compound **L1** did not bind at 20 μM but has a higher solubility, it was further tested in a saturation MST experiment which also concluded no binding to IspE.

As an orthogonal method to determine qualitative binding of the hits, we performed thermal shift assays. The protein needs to reach a certain level of saturation in order to exhibit a change in melting temperature (Δ*T*_m_), therefore compounds with a solubility lower than 20 μM (**E4**, **E1**, **I1**) could not be tested. Minimal Δ*T*_m_ values were obtained and have to be interpreted carefully, since they are close to the inherent error of the method. **K1**, **K4** and **E6** showed Δ*T*_m_ = +0.7 °C, providing some assurance in the binding of **K1**, which shows a consistent response in both methods.

#### Antibacterial Activity against *M. tuberculosis* and *E. coli*

*M. tuberculosis* is known to be notoriously hard to target,^22^ and once again our compounds‘ poor solubility posed a significant obstacle to their antibacterial evaluation. Nevertheless, our final goal is to obtain antitubercular compounds. Therefore, we assessed the antitubercular activity of our hits at the concentration of their maximum solubility in 7H9 growth medium (Table 1). Regretfully, no compound presented inhibitory activity against *Mtb*H37Rv (Table 1). This lack of inhibitory effects may be attributed to permeability and efflux issues inherent to the pathogen. It is noteworthy that assay conditions were constrained, only one compound could be tested at high concentrations (64 μM) and almost half of our compounds could not be tested at all (Table 1).

The identity of *Mtb*IspE and its *E. coli* ortholog are high,^21^ therefore we decided to test the TolC efflux pump deficient *E. coli* strain (*Ec*TolC) to obtain further insights into our compounds’ antibacterial profile. To assess possible efflux issues of our compounds, we tested the *Ec*TolC active compounds against the *E. coli* wild-type strain K12. All compounds, regardless of their solubilities, were tested at 50 μM against the *E. coli* strains. Seven of our hits displayed moderate inhibition of *Ec*TolC, with more than 10% inhibition, including **K1** that was shown to bind to IspE. Notably, compound **L1** demonstrated the most promising result with 52% inhibition, which may be due to its higher solubility compared to most other active compounds. **L1** is also the only compound showing an appreciable effect on the growth of *E. coli* K12 wild type, indicating that the structures are prone to efflux. Additionally, a toxicity assessment conducted on HepG2 cells revealed that the majority of the compounds are nontoxic. Nevertheless, compounds **D1** and **K1** exhibited notable cytotoxicity, with IC_50_ values below 10 μM. These findings provide valuable insights into the inhibitory potential and safety profile of the compounds tested, emphasizing the need for further exploration of these potential antibacterial starting points.

## Conclusions

We have successfully demonstrated the compatibility of *Mtb*IspE with acylhydrazone-based tdDCC conditions, providing a solid foundation for further exploration in drug-discovery efforts using pathogenic targets for hit-identification. In addition, we optimized the analysis of the tdDCC equilibrium for the determination of hit compounds. We identified the majority of the 72 possible *N*-acylhydrazones using HPLC-MS/MS and *in silico* evaluation of these components gave insights into the possible binding modes of our privileged scaffolds **1** and **E**. The most challenging aspect in our work was the hit validation due to the poor solubility of our 11 tdDCC hits. Despite this challenge, our study successfully verified one compound, **K1**, as targeting IspE and it also showed moderate activity on *Ec*TolC. Compound **L1** turned out to be the most potent inhibitor of *E. coli* growth. These positive outcomes position **K1** and **L1** as starting points for further optimization. Since the acylhydrazone moiety is likely to be the major contributor to poor solubility and thus is a critical point of improvement, we propose its replacement with bioisosteres such as oxazoles or amides.^23^ For future endeavors, however, we suggest a more careful building-block selection, including considerations such as logS values. Furthermore, contemplating alternative reactions to create dynamic combinatorial libraries with a more soluble linker should be considered as well. We urge for continuous refinement and exploration in our goal to develop anti-infective agents.

## Experimental Section

### Characterization of Products

#### (E)-2-(3,5-Dimethyl-1H-pyrazol-1-yl)-*N*′-((5-(4-methoxyphenyl)isoxazol-3-yl)methylene)acetohydrazide (**B6**)

^1^H NMR indicates the presence of two isomers, possibly *trans* and *cis* conformers of the amide. We did not assign the peaks to a specific conformer and will refer to them as *A* and *B* (*A*:*B* = 2:1).^1^H NMR (400 MHz, DMSO-*d*_*6*_) δ = 11.99 (br s, 1H, *A*&*B*), 8.33 (s, 1H, *B*), 8.09 (s, 1H, *A*), 7.91 (br d, *J* = 8.6 Hz, 2H, *B*), 7.87 (br d, *J* = 8.2 Hz, 2H, *A*), 7.34 (s, 1H, *A*), 7.21 (s, 1H, *B*), 7.11 (br d, *J* = 8.2 Hz, 2H, *A*), 7.07 (br s, 2H, *B*), 5.84 (s, 1H, *A*&*B*), 5.26 (s, 1H, *A*), 4.80 (s, 1H, *B*), 3.83 (s, 3H, *A*&*B*), 2.20 (s, 1H, *B*), 2.16 (s, 3H, *A*), 2.08 (s, 3H, *A*&*B*) ppm.^13^C NMR (101 MHz, DMSO-*d*_*6*_) δ = 169.5, 169.0, 161.1, 160.7, 146.0, 140.1, 133.6, 127.4, 119.1, 114.7, 104.8, 95.9, 55.4, 49.3, 13.3, 10.6 ppm.

#### (E)-*N*′-(5-Chloro-2-hydroxybenzylidene)-2-(4-methylpiperazin-1-yl)acetohydrazide (**C1**)

^1^H NMR (400 MHz, CHLOROFORM-*d*): δ = 10.11 (br s, 1H), 8.41 (s, 1H), 7.21–7.25 (m, 1H), 7.18 (d, *J* = 2.3 Hz, 1H), 6.93 (d, *J* = 8.6 Hz, 1H), 3.46–3.49 (m, 1H), 3.20 (s, 2H), 2.60–2.68 (m, 4H), 2.51 (br s, 4H), 2.32 (s, 3H) ppm. ^13^C NMR (101 MHz, CHLOROFORM-*d*): δ = 166.0, 157.0, 149.7, 131.6, 129.9, 123.9, 118.7, 118.4, 60.9, 55.0, 53.6, 45.9 ppm.

#### (E)-*N*′-(5-Chloro-2-hydroxybenzylidene)-3-isobutyl-1H-pyrazole-5-carbohydrazide (**D1**)

^1^H NMR (400 MHz, DMSO-*d*_*6*_) δ = 13.14 (br s, 1H), 12.05 (s, 1H), 11.39 (s, 1H), 8.64 (s, 1H), 7.56 (br s, 1H), 7.30 (dd, *J* = 8.6, 2.5 Hz, 1H), 6.94 (d, *J* = 8.6 Hz, 1H), 6.54 (s, 1H), 2.53 (br d, *J* = 7.0 Hz, 2H), 1.91 (spt, *J* = 6.6 Hz, 1H), 0.89 (d, *J* = 6.6 Hz, 6H) ppm. ^13^C NMR (101 MHz, DMSO-*d*_*6*_) δ = 158.4, 156.0, 145.8, 145.2, 143.8, 130.5, 127.9, 122.8, 120.7, 118.2, 104.7, 33.8, 28.2, 22.0 ppm.

#### (E)-3-Isobutyl-*N*′-((5-(4-methoxyphenyl)isoxazol-3-yl)methylene)-1H-pyrazole-5-carbohydrazide (**D6**)

^1^H NMR (400 MHz, DMSO-*d*_*6*_) δ = 13.17 (s, 1H), 12.08 (s, 1H), 8.62 (s, 1H), 7.92 (d, *J* = 8.7 Hz, 2H), 7.24 (s, 1H), 7.09 (d, *J* = 8.7 Hz, 2H), 6.56 (s, 1H), 3.84 (s, 3H), 2.54 (br d, *J* = 7.0 Hz, 2H), 1.92 (spt, *J* = 6.6 Hz, 1H), 0.90 (d, *J* = 6.6 Hz, 6H) ppm. ^13^C NMR (101 MHz, DMSO-*d*_*6*_) δ = 169.6, 161.2, 161.0, 158.6, 145.3, 144.0, 137.0, 127.5, 119.2, 114.7, 104.9, 95.8, 55.4, 33.8, 28.2, 22.0 ppm.

#### (E)-5-Amino-*N*′-(5-chloro-2-hydroxybenzylidene)-1-(3-(trifluoromethyl)phenyl)-1H-1,2,3-triazole-4-carbohydrazide (**E1**)

^1^H NMR (500 MHz, DMSO-*d*_*6*_) δ = 12.43 (br s, 1H), 11.48 (br s, 1H), 8.66 (s, 1H), 8.00 (s, 1H), 7.92–7.97 (m, 2H), 7.87 (t, *J* = 7.8 Hz, 1H), 7.57 (br s, 1H), 7.31 (dd, *J* = 8.1, 2.3 Hz, 1H), 6.95 (d, *J* = 8.1 Hz, 1H), 6.83 (br s, 2H) ppm. ^13^C NMR (126 MHz, DMSO-*d*_*6*_) δ = 158.3, 156.0, 146.0, 145.7, 135.2, 131.1, 130.4, 128.7, 128.1, 126.0, 124.7, 122.8, 121.5, 120.6, 120.1, 118.2 ppm. ^19^F NMR (470 MHz, DMSO-*d*_*6*_) δ = −61.13 ppm.

#### (E)-5-Amino-*N*′-(4-(trifluoromethyl)benzylidene)-1-(3-(trifluoromethyl)phenyl)-1H-1,2,3-triazole-4-carbohydrazide (**E4**)

^1^H NMR, ^19^F NMR, and HPLC-MS indicate the presence of two isomers, possibly *trans* and *cis* conformers of the amide. We did not assign the peaks to a specific conformer and will refer to them as *A* and *B* (*A*:*B* = 1:1). ^1^H NMR (500 MHz, DMSO-*d*_*6*_) δ = 12.30 (br s, 1H, *A*), 12.17 (s, 1H, *B*), 8.88 (s, 1H, *A*), 8.59 (br s, 2H, *A*), 8.13 (br s, 1H, *A*), 8.00 (s, 1H, *B*), 7.87–7.98 (m, 7H, *A* & *B*), 7.77–7.86 (m, 6H, *A* & *B*), 7.52 (br t, *J* = 7.6 Hz, 1H, *B*), 7.23 (br d, *J* = 7.6 Hz, 1H, *B*), 6.78 ppm (s, 2H, *B*). ^13^C NMR (126 MHz, DMSO-*d*_*6*_) δ = 158.6, 146.2, 146.0, 141.9, 138.6, 135.2, 131.2, 130.0, 129.6, 128.7, 127.6, 127.5, 125.8, 123.1, 121.5, 120.4, 120.2, 112.1 ppm. ^19^F NMR (470 MHz, DMSO-*d*_6_) δ = −61.11 (s, 3F, *A*), −61.13 (s, 3F, *B*), −61.17 (s, 3F, *A*), −61.24 (s, 3F, *B*) ppm.

#### (E)-5-Amino-*N*′-((5-(4-methoxyphenyl)isoxazol-3-yl)methylene)-1-(3-(trifluoromethyl)phenyl)-1H-1,2,3-triazole-4-carbohydrazide (**E6**)

^1^H NMR (500 MHz, DMSO-*d*_6_): δ = 12.41 (s, 1H), 8.63 (s, 1H), 8.01 (s, 1H), 7.94–7.98 (m, 2H), 7.93 (d, *J* = 8.5 Hz, 2H), 7.88 (t, *J* = 8.1 Hz, 1H), 7.22 (s, 1H), 7.10 (d, *J* = 8.5 Hz, 2H), 6.81 (s, 2H), 3.84 (s, 3H) ppm. ^13^C NMR (126 MHz, DMSO-*d*_*6*_): δ = 169.6, 161.2, 161.0, 158.6, 146.2, 136.6, 135.2, 131.2, 130.3, 128.8, 127.6, 126.1, 121.59, 121.57, 120.2, 119.2, 114.7, 95.7, 55.4 ppm. ^19^F NMR (470 MHz, DMSO-*d*_6_): δ = −61.12 ppm.

#### (E)-5-(2-(5-Chloro-2-hydroxybenzylidene)hydrazine-1-carbonyl)-*N*-(3,4-dimethoxyphenyl)-1-methyl-1H-pyrazole-3-carboxamide (**H1**)

^1^H NMR (500 MHz, DMSO-*d*_*6*_) δ = 12.28 (br s, 1H), 11.07 (br s, 1H), 10.06 (s, 1H), 8.62 (s, 1H), 7.69 (d, *J* = 2.4 Hz, 1H), 7.52 (s, 1H), 7.51 (d, *J* = 2.1 Hz, 1H), 7.43 (dd, *J* = 8.7, 2.1 Hz, 1H), 7.33 (dd, *J* = 8.7, 2.4 Hz, 1H), 6.96 (d, *J* = 8.7 Hz, 1H), 6.91 (d, *J* = 8.7 Hz, 1H), 4.23 (s, 3H), 3.75 (s, 3H), 3.73 (s, 3H) ppm. ^13^C NMR (126 MHz, DMSO-*d*_*6*_) δ = 158.9, 156.0, 155.2, 148.5, 146.1, 145.2, 145.0, 135.4, 132.2, 131.1, 127.2, 123.1, 120.8, 118.3, 112.2, 111.9, 108.8, 105.6, 55.7, 55.4, 39.8 ppm.

#### (1R,3S,5R,7S)-3-(3-Chloro-1H-1,2,4-triazol-1-yl)-*N*′-((Z)-5-chloro-2-hydroxybenzylidene)adamantane-1-carbohydrazide (**I1**)

^1^H NMR (500 MHz, DMSO-*d*_*6*_): δ = 11.35 (s, 1H), 11.28 (s, 1H), 8.70 (s, 1H), 8.52 (s, 1H), 7.59 (d, *J* = 2.6 Hz, 2H), 7.29 (dd, *J* = 8.8, 2.6 Hz, 2H), 6.92 (d, *J* = 8.8 Hz, 1H), 2.32 (br s, 1H), 2.25 (s, 1H), 2.13 (d, *J* = 12.0 Hz, 2H), 2.07 (d, *J* = 12.0 Hz, 2H), 1.93 (d, *J* = 11.6 Hz, 2H), 1.87 (d, *J* = 11.6 Hz, 2H), 1.70 (br s, 2H) ppm. ^13^C NMR (126 MHz, DMSO-*d*_6_): δ = 171.6, 156.0, 150.2, 145.2, 142.9, 130.6, 127.6, 122.9, 120.6, 118.2, 59.6, 42.4, 41.8, 40.4, 36.8, 34.2, 28.6 ppm.

#### (E)-3-Chloro-*N*′-(5-chloro-2-hydroxybenzylidene)benzohydrazide (**K1**)

^1^H NMR (400 MHz, DMSO-*d*_*6*_) δ = 12.24 (s, 1H), 11.19 (br s, 1H), 8.63 (s, 1H), 7.98 (t, *J* = 1.6 Hz, 1H), 7.90 (dt, *J* = 7.8, 1.6 Hz, 1H), 7.66–7.70 (m, 2H), 7.58 (t, *J* = 7.8 Hz, 1H), 7.32 (dd, *J* = 8.9, 2.7 Hz, 1H), 6.96 (d, *J* = 8.9 Hz, 1H) ppm. ^13^C NMR (101 MHz, DMSO-*d*_*6*_) δ = 161.6, 156.1, 146.1, 134.8, 133.4, 131.9, 131.0, 130.6, 127.4, 126.6, 123.1, 120.7, 118.3, 109.6 ppm.

#### (E)-3-Chloro-*N*′-(4-(trifluoromethyl)benzylidene)benzohydrazide (**K4**)

^1^H NMR (400 MHz, DMSO-*d*_*6*_) δ = 12.12 (br s, 1H), 8.52 (s, 1H), 7.93–7.99 (m, 3H), 7.89 (br d, *J* = 7.8 Hz, 1H), 7.81 (br d, *J* = 8.2 Hz, 2H), 7.68 (d, *J* = 7.8 Hz, 1H), 7.58 (t, *J* = 7.8 Hz, 1H) ppm. ^13^C NMR (126 MHz, DMSO-*d*_*6*_) δ = 161.9, 146.6, 138.2, 135.2, 133.3, 131.8, 130.6, 129.8, 127.8, 127.4, 126.6, 125.8, 124.1 ppm. ^19^F NMR (470 MHz, DMSO-*d*_*6*_) δ = −61.20 ppm.

#### (E)-*N*′-(5-Chloro-2-hydroxybenzylidene)isonicotinohydrazide (**L1**)

^1^H NMR (400 MHz, DMSO-*d*_*6*_) δ = 12.35 (s, 1H), 11.11 (s, 1H), 8.80 (d, *J* = 6.2 Hz, 2H), 8.66 (s, 1H), 7.84 (d, *J* = 6.2 Hz, 2H), 7.70 (d, *J* = 2.5 Hz, 1H), 7.33 (dd, *J* = 8.6, 2.5 Hz, 1H), 6.96 (d, *J* = 8.6 Hz, 1H) ppm. ^13^C NMR (101 MHz, DMSO-*d*_*6*_) δ = 161.5, 156.1, 150.4, 146.5, 139.9, 131.1, 127.2, 123.1, 121.5, 120.7, 118.3 ppm.

### General Procedure for tdDCC Experiments

To a well of a 24-well plate, containing Tris buffer (pH 7.0) were added hydrazides (300 μM each, in DMSO), aldehydes (100 μM each, in DMSO), aniline (20 mM, in DMSO), the protein *Mtb*IspE (25 or 40 μM in Tris buffer at pH 7.0), and an additional amount of DMSO to reach a final concentration of 5% in the DCL with 0.5 mL of end-volume. The DCL was allowed to mix on a plate shaker at room temperature and was frequently monitored via LCMS-MS. For analysis, 30 μL of the corresponding library was mixed with 42 μL acetonitrile and 3 μL of NaOH (1 M), the mixture was centrifuged, and the supernatant was used for the analysis. PT experiments were run in duplicates and the protein was omitted for the blank experiments.

## Supporting Information Available

Detailed materials and methods, analytical details of products, and ^1^H, ^13^C, and ^19^F-NMR spectra (PDF)
