# Supplementary material for: Target-Directed Dynamic Combinatorial Chemistry Affords Binders of Mycobacterium tuberculosis IspE
Source: ACS Omega. 2024 Aug 29;9(36):38160–8. doi: 10.1021/acsomega.4c05537 (PMC11391541; doi:10.1021/acsomega.4c05537)
Supplement: Supplementary file 1 — ao4c05537_si_001.pdf [file ao4c05537_si_001.pdf]

Supporting Information

Target-Directed Dynamic Combinatorial Chemistry Affords Binders of *Mycobacterium tuberculosis* IspE

Maria Braun-Cornejo,[1,2,3] Camilla Ornago,[4] Vidhisha Sonawane,[5] Jörg Haupenthal,[3] Andreas M. Kany,[3] Eleonora Diamanti,[3] Gwenaëlle Jézéquel,[3] Norbert Reiling,[5] Wulf Blankenfeldt,[4] Peter Maas,[1] and Anna K. H. Hirsch [2,3*]

[1] *Specs Compound Handling, B.V., Bleiswijkseweg 55, 2712 PB, Zoetermeer, The Netherlands.*

[2] *Saarland University, Department of Pharmacy, Campus Building E8.1, 66123 Saarbrücken, Germany.*

[3] *Helmholtz Institute for Pharmaceutical Research Saarland (HIPS) – Helmholtz Centre for Infection Research (HZI), Campus Building E8.1, 66123 Saarbrücken, Germany.*

[4] *Department Structure and Function of Proteins Helmholtz Centre for Infection Research Inhoffenstrasse 7, 38124 Braunschweig, Germany.*

[5] *RG Microbial Interface Biology, Research Center Borstel Leibniz Lung Center, Parkallee 1-40, Borstel, 23845, Sülfeld, Germany.*

*Corresponding author: Anna.Hirsch@helmholtz-hips.de

Table of Contents

Supplementary Methods 3
- General information 3
- Chemicals 3
- Experimental conditions of tdDCC 3
- Assessment of amplification factors 4
- General procedure for acylhydrazones synthesis 4
- Characterization of acylhydrazone products 4
- *Mtb*IspE expression and purification 8
- *Mtb*IspE stability study *via* thermal shift assay 8
- *In silico* elucidation of tdDCC hits' binding mode 9
- Kinetic turbidimetric solubility 9
- *Mtb*IspE binding study *via* MicroScale Thermophoresis 9
- *Mtb*IspE binding study *via* thermal shift assay 9
- Determination of *E.coli* activity 9
- Determination of *in vitro* anti-tubercular activity and solubility in 7H9 medium 10
- Cytotoxicity assay 10

Supplementary Figures 11
- Selection of previously published IspE inhibitors 11
- *Mtb*IspE stability study *via* thermal shift assay 11
- HPLC-MS/MS peak assignments and determination of equilibrium 12
- Amplification of *N*-acylhydrazones in the PT experiments 17

In silico elucidation of tdDCC hits' binding mode....18
NMR, HRMS, and LCMS spectra of acylhydrazone products....19
Compound **B6**....19
Compound **C1**....20
Compound **D1**....22
Compound **D6**....24
Compound **E1**....25
Compound **E4**....27
Compound **E6**....30
Compound **H1**....32
Compound **I1**....33
Compound **K1**....35
Compound **K4**....37
Compound **L1**....39
Supplementary references....40

Supplementary Methods

General information

High-resolution mass (HRMS) of final products was determined by HPLC-MS/MS using a Thermo Scientific Q Exactive Focus Orbitrap LC-MS/MS system. All compounds were analyzed for purity by LCMS on Acquity UPLC-SQD system from Waters with a gradient elution of Water (Formic acid 0.1%)/Acetonitrile on an HSS-T3 column (2.1 x 50 mm, Waters), 1.8 µm, at 30°C, PDA detection between 240-320 nm, and MS detection by simultaneous ES+/ES- ionization in a mass range of 150-800. The flow is set to 0.9 ml/min, and the gradient time is 1.5 min. NMR spectra were recorded on an Agilent 400 MHz or a Bruker Avance Neo 500 MHz. Chemical shifts (δ) are reported in ppm relative to residual solvent signals. The following abbreviations are used to describe peak patterns when appropriate: s (singlet), d (doublet), t (triplet), q (quartet), quint (quintet), sex (sextet), sept (septuplet), m (multiplet), br (broad). Coupling constants (*J*) are reported in Hertz (Hz). Reactions were monitored with thin layer chromatography (TLC) on silica gel-coated aluminum (silica gel F254, SiliCycle).
The periodic progress and analysis of tdDCC were monitored by HPLC-MS/MS (ThermoScientific Dionex Ultimate 3000 UHPLC System coupled to a ThermoScientific Q Exactive Focus with an electrospray ion source) using an Acquity Waters Column (BEH, C8 1.7 µm, 2.1 × 150 mm, Waters, Germany) at a flow rate of 0.250 mL/min with detection set at 210, 254, 290, and 310 nm, and the mass spectrum recorded in a positive mode in the range of 100−700 m/z. The solvent system was 0.1% formic acid in H_2O (Solvent-A) and 0.1% formic acid in MeCN (Solvent-B). The gradient program began with 5% of Solvent-B for 1 min and was then increased to 95% of Solvent-B over 17 min and held for 2 min, followed by a decrease of Solvent-B to 5% over 0.1 min, where it was held for 2 min.

Chemicals

All reagents and solvents were purchased from Sigma-Aldrich, Specs, Fluorochem, or Acros Organics, were reagent grade, and used without purification unless indicated otherwise. All reactions were conducted under nitrogen atmosphere using oven-dried glassware. Purifications by flash chromatography were done using silica gel (Screening Devices 60-200 µm). All new compounds were fully characterised by ^{1}H and ^{13}C NMR and HRMS techniques. The purity of the final products was determined by HPLC-MS and found to be >95%.

Experimental conditions of tdDCC

DCL-1:
This experiment library consists of three aldehydes (**1**−**3**) and six hydrazides (**A**−**F**). The composition of DCL-1 is as below:

Entry	*Blank*		*PT-I*		*PT-II*	
	amount	final conc. in DCL	amount	final conc. in DCL	amount	final conc. in DCL
Tris buffer	475 µL	-	436.3 µL	-	436.3 µL	-
Hydrazide (100 mM)	6 × 1.5 µL (9 µL)	(6 × 300 µM)	6 × 1.5 µL (9 µL)	(6 × 300 µM)	6 × 1.5 µL (9 µL)	(6 × 300 µM)
Aldehyde (100 mM)	3 × 0.5 µL (1.5 µL)	(3 × 100 µM)	3 × 0.5 µL (1.5 µL)	(3 × 100 µM)	3 × 0.5 µL (1.5 µL)	(3 × 100 µM)
Aniline (1 M)	10 µL	20 mM	10 µL	20 mM	10 µL	20 mM
DMSO	4.5 µL	5%	4.5 µL	5%	4.5 µL	5%
MtbIspE (324 µM)	0	-	38.7 µL	25 µM	38.7 µL	25 µM

DCL-2: This experiment library consists of three aldehydes (**4**−**6**) and six hydrazides (**G**−**L**).
DCL-3: This experiment library consists of three aldehydes (**4**−**6**) and six hydrazides (**A**−**F**).
DCL-4: This experiment library consists of three aldehydes (**1**−**3**) and six hydrazides (**B**−**F**, and **M**).

The composition of DCL-2,-3, and-4 is as below:

Entry	*Blank*		*PT-I*		*PT-II*	
	amount	final conc. in DCL	amount	final conc. in DCL	amount	final conc. in DCL
Tris buffer	475 μL	-	413.2 μL	-	413.2 μL	-
Hydrazide (100 mM)	6 × 1.5 μL (9 μL)	(6 × 300 μM)	6 × 1.5 μL (9 μL)	(6 × 300 μM)	6 × 1.5 μL (9 μL)	(6 × 300 μM)
Aldehyde (100 mM)	3 × 0.5 μL (1.5 μL)	(3 × 100 μM)	3 × 0.5 μL (1.5 μL)	(3 × 100 μM)	3 × 0.5 μL (1.5 μL)	(3 × 100 μM)
Aniline (1 M)	10 μL	20 mM	10 μL	20 mM	10 μL	20 mM
DMSO	4.5 μL	5%	4.5 μL	5%	4.5 μL	5%
MtbIspE (324 μM)	0	-	61.8 μL	40 μM	61.8 μL	40 μM

Assessment of amplification factors

The amplification factor for each product of the DCL was assessed at the time of equilibrium of the blank experiment. The relative peak area (RPA) was determined for each experiment; blank and protein-templated (PT) duplicates, using ACD/Labs (Figure S2–S5, p. 12). All products of the DCL were integrated and their sum set to 100% in order to obtain the respective RPA for each product. The amplification factor was calculated for each PT duplicate individually as $\left(\frac{RPA_{PT} - RPA_{blank}}{RPA_{blank}}\right)$.[2] The mean of the amplification factors and its standard deviation were reported for each product in form of bar charts with error bars, for DCL-1 in the main text (Figure 3A) and for DCL-2, -3, and -4 in Figure S9 (p. 17).

General procedure for acylhydrazones synthesis

General procedure for acylhydrazone formation (GP-3):
To an oven-dried round-bottom flask, under nitrogen atmosphere, the corresponding hydrazide (1.0 eq) and aldehyde (1.0–1.8 eq) were suspended in MeOH (0.02–0.2 M) and refluxed for 1–5 h; its progress was followed on TLC. After the reaction was completed, the solvent was removed under reduced pressure to afford the corresponding acylhydrazone product in 85 – 99% isolated yields.

General procedure for small scale acylhydrazone formation (GP-4):
To an oven-dried test tube with a magnetic stirrer, under nitrogen atmosphere the corresponding hydrazide (1.0 eq) and aldehyde (1.0–1.1 eq) were suspended in MeOH (0.02–0.12 M). The reaction mixture was refluxed for 2–5 h, using Radleys Parallel Synthesiser™. The reaction progress was followed by TLC. Once the reaction was completed, the mixture was cooled to 0 °C and the precipitate filtered over a 20 μm filter and washed with cold MeOH. The residual solvent was removed under reduced pressure to afford the corresponding acylhydrazone product in 28 – 99% isolated yields.

Characterization of acylhydrazone products

(*E*)-2-(3,5-diMethyl-1*H*-pyrazol-1-yl)-*N*'-((5-(4-methoxyphenyl)isoxazol-3-yl)methylene)acetohydrazide (**B6**):

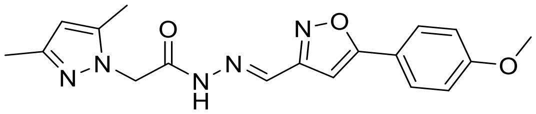

Compound **B6** was synthesised according to GP-4, hydrazide **B** (12.8 mg, 0.076 mmol) and aldehyde **6** (15.7 mg, 0.077 mmol) stirred in MeOH (1.5 mL) for 5 h to afford compound **B6** as white airy powder (18.9 mg, 0.054 mmol, 70%). ^{1}H NMR indicates the presence of two isomers, possibly *trans* and *cis* conformers of the amide. We did not assign the peaks to a specific conformer and will refer to them as *A* and *B* (*A*:*B* = 2:1).
^{1}H NMR (400 MHz, DMSO-d_6) δ = 11.99 (br s, 1H, *A*&*B*), 8.33 (s, 1H, *B*), 8.09 (s, 1H, *A*), 7.91 (br d, *J* = 8.6 Hz, 2H, *B*), 7.87 (br d, *J* = 8.2 Hz, 2H, *A*), 7.34 (s, 1H, *A*), 7.21 (s, 1H, *B*), 7.11 (br d, *J* = 8.2 Hz, 2H, *A*), 7.07 (br s,

2H, *B*), 5.84 (s, 1H, *A*&*B*), 5.26 (s, 1H, *A*), 4.80 (s, 1H, *B*), 3.83 (s, 3H, *A*&*B*), 2.20 (s, 1H, *B*), 2.16 (s, 3H, *A*), 2.08 (s, 3H, *A*&*B*) ppm.
^{13}C NMR (101 MHz, DMSO-d_6) δ = 169.5, 169.0, 161.1, 160.7, 146.0, 140.1, 133.6, 127.4, 119.1, 114.7, 104.8, 95.9, 55.4, 49.3, 13.3, 10.6 ppm.
HRMS (ESI$^+$): *m/z* calcd. for $C_{18}H_{20}N_5O_3^+$ ([*M*+H]$^+$) 354.1561, measured 354.1543.

(*E*)-*N*'-(5-Chloro-2-hydroxybenzylidene)-2-(4-methylpiperazin-1-yl)acetohydrazide (**C1**):

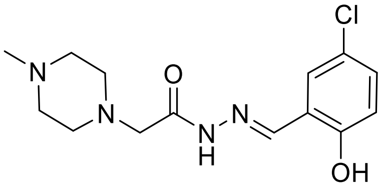

Compound **C1** was synthesised according to GP-4, with adaptations. Hydrazide **C** (21.0 mg, 0.122 mmol) and aldehyde **1** (19.1 mg, 0.122 mmol) stirred in MeOH (1 mL) for 2 h. The solvent was removed under reduced pressure to afford compound **C1** as yellow oil (37.9 mg, 0.122 mmol, >99%).
^{1}H NMR (400 MHz, CHLOROFORM-*d*): δ = 10.11 (br s, 1H), 8.41 (s, 1H), 7.21-7.25 (m, 1H), 7.18 (d, *J* = 2.3 Hz, 1H), 6.93 (d, *J* = 8.6 Hz, 1H), 3.46-3.49 (m, 1H), 3.20 (s, 2H), 2.60-2.68 (m, 4H), 2.51 (br s, 4H), 2.32 (s, 3H) ppm.
^{13}C NMR (101 MHz, CHLOROFORM-*d*): δ = 166.0, 157.0, 149.7, 131.6, 129.9, 123.9, 118.7, 118.4, 60.9, 55.0, 53.6, 45.9 ppm.
HRMS (ESI$^+$): *m/z* calcd. for $C_{14}H_{20}ClN_4O_2^+$ ([*M*+H]$^+$) 311.1269, measured 311.1254.

(*E*)-*N*'-(5-Chloro-2-hydroxybenzylidene)-3-isobutyl-1*H*-pyrazole-5-carbohydrazide (**D1**):

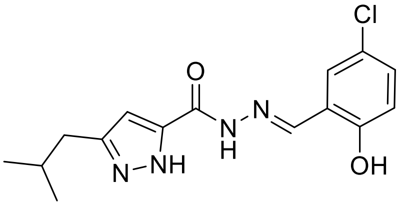

Compound **D1** was synthesised according to GP-3, hydrazide **D** (51.7 mg, 0.284 mmol) and aldehyde **1** (46.5 mg, 0.297 mmol) stirred in MeOH (5 mL) for 2.5 h to afford compound **D1** as pale yellow solid (88.8 mg, 0.277 mmol, 98%).
^{1}H NMR (400 MHz, DMSO-d_6)*δ* = 13.14 (br s, 1H), 12.05 (s, 1H), 11.39 (s, 1H), 8.64 (s, 1H), 7.56 (br s, 1H), 7.30 (dd, *J* = 8.6, 2.5 Hz, 1H), 6.94 (d, *J* = 8.6 Hz, 1H), 6.54 (s, 1H), 2.53 (br d, *J* = 7.0 Hz, 2H), 1.91 (spt, *J* = 6.6 Hz, 1H), 0.89 (d, *J* = 6.6 Hz, 6H) ppm.
^{13}C NMR (101 MHz, DMSO-d_6) *δ* = 158.4, 156.0, 145.8, 145.2, 143.8, 130.5, 127.9, 122.8, 120.7, 118.2, 104.7, 33.8, 28.2, 22.0 ppm.
HRMS (ESI$^+$): *m/z* calcd. for $C_{15}H_{18}ClN_4O_2^+$ ([*M*+H]$^+$) 321.1113, measured 321.1105.

(*E*)-3-Isobutyl-*N*'-((5-(4-methoxyphenyl)isoxazol-3-yl)methylene)-1*H*-pyrazole-5-carbohydrazide (**D6**):

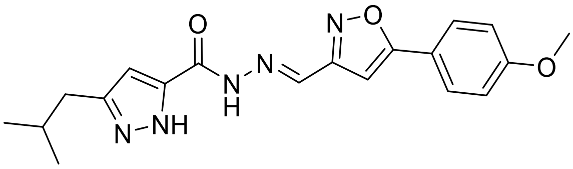

Compound **D6** was synthesised according to GP-3, hydrazide **D** (47.0 mg, 0.258 mmol) and aldehyde **6** (53.1 mg, 0.261 mmol) stirred in MeOH (5 mL) for 2.5 h to afford compound **D6** as white powder (92.0 mg, 0.250mmol, 97%).
^{1}H NMR (400 MHz, DMSO-d_6) *δ* = 13.17 (s, 1H), 12.08 (s, 1H), 8.62 (s, 1H), 7.92 (d, *J* = 8.7 Hz, 2H), 7.24 (s, 1H), 7.09 (d, *J* = 8.7 Hz, 2H), 6.56 (s, 1H), 3.84 (s, 3H), 2.54 (br d, *J* = 7.0 Hz, 2H), 1.92 (spt, *J* = 6.6 Hz, 1H), 0.90 (d, *J* = 6.6 Hz, 6H) ppm.
^{13}C NMR (101 MHz, DMSO-d_6) *δ* = 169.6, 161.2, 161.0, 158.6, 145.3, 144.0, 137.0, 127.5, 119.2, 114.7, 104.9, 95.8, 55.4, 33.8, 28.2, 22.0 ppm.
HRMS (ESI$^+$): *m/z* calcd. for $C_{19}H_{22}N_5O_3^+$ ([*M*+H]$^+$) 368.1717, measured 368.1701.

(E)-5-Amino-N'-(5-chloro-2-hydroxybenzylidene)-1-(3-(trifluoromethyl)phenyl)-1H-1,2,3-triazole-4-carbohydrazide (**E1**):

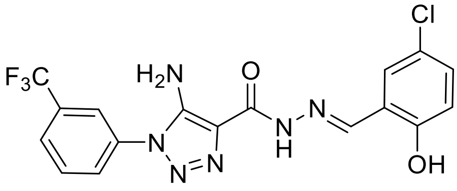

Compound **E1** was synthesised according to GP-4, hydrazide **E** (10.0 mg, 0.035 mmol) and aldehyde **1** (6.1 mg, 0.039 mmol) stirred at reflux in MeOH (1.5 mL) for 5 h and at rt overnight to afford compound **E1** as off-white solid (11.4 mg, 0.027mmol, 77%).

^{1}H NMR (500 MHz, DMSO-d_6) δ = 12.43 (br s, 1H), 11.48 (br s, 1H), 8.66 (s, 1H), 8.00 (s, 1H), 7.92-7.97 (m, 2H), 7.87 (t, J = 7.8 Hz, 1H), 7.57 (br s, 1H), 7.31 (dd, J = 8.1, 2.3 Hz, 1H), 6.95 (d, J = 8.1 Hz, 1H), 6.83 (br s, 2H) ppm.

^{13}C NMR (126 MHz, DMSO-d_6) δ = 158.3, 156.0, 146.0, 145.7, 135.2, 131.1, 130.4, 128.7, 128.1, 126.0, 124.7, 122.8, 121.5, 120.6, 120.1, 118.2 ppm.

^{19}F NMR (470 MHz, DMSO-d_6) δ = -61.13 ppm.

HRMS (ESI$^+$): m/z calcd. for $C_{17}H_{13}ClF_3N_6O_2^+$ ([M+H]$^+$) 425.0735, measured 425.0726.

(E)-5-Amino-N'-(4-(trifluoromethyl)benzylidene)-1-(3-(trifluoromethyl)phenyl)-1H-1,2,3-triazole-4-carbohydrazide (**E4**):

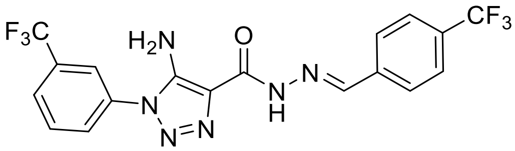

Compound **E4** was synthesised according to GP-4, hydrazide **E** (10.0 mg, 0.035 mmol) and aldehyde **4** (6.3 mg, 0.036 mmol) stirred at reflux in MeOH (1.5 mL) for 5h and at rt overnight to afford compound **E4** as off-white solid (4.3 mg, 0.010 mmol, 28%). ^{1}H NMR, ^{19}F NMR, and HPLC-MS indicate the presence of two isomers, possibly *trans* and *cis* conformers of the amide. We did not assign the peaks to a specific conformer and will refer to them as A and B (A:B = 1:1).

^{1}H NMR (500 MHz, DMSO-d_6) δ = 12.30 (br s, 1H, A), 12.17 (s, 1H, B), 8.88 (s, 1H, A), 8.59 (br s, 2H, A), 8.13 (br s, 1H, A), 8.00 (s, 1H, B), 7.87-7.98 (m, 7H, A & B), 7.77-7.86 (m, 6H, A & B), 7.52 (br t, J = 7.6 Hz, 1H, B), 7.23 (br d, J = 7.6 Hz, 1H, B), 6.78 ppm (s, 2H, B).

^{13}C NMR (126 MHz, DMSO-d_6) δ = 158.6, 146.2, 146.0, 141.9, 138.6, 135.2, 131.2, 130.0, 129.6, 128.7, 127.6, 127.5, 125.8, 123.1, 121.5, 120.4, 120.2, 112.1 ppm.

^{19}F NMR (470 MHz, DMSO-d$_6$) δ = -61.11 (s, 3F, A), -61.13 (s, 3F, B), -61.17 (s, 3F, A), -61.24 (s, 3F, B) ppm.

HRMS (ESI$^+$): m/z calcd. for $C_{18}H_{13}F_6N_6O^+$ ([M+H]$^+$) 443.1050, measured 443.1022.

(E)-5-Amino-N'-((5-(4-methoxyphenyl)isoxazol-3-yl)methylene)-1-(3-(trifluoromethyl)phenyl)-1H-1,2,3-triazole-4-carbohydrazide (**E6**):

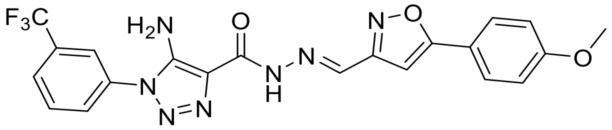

Compound **E6** was synthesised according to GP-4, hydrazide **E** (10.0 mg, 0.035 mmol) and aldehyde **6** (7.1 mg, 0.035 mmol) stirred at reflux in MeOH (1.5 mL) for 5 h and at rt overnight to afford compound **E6** as off-white solid (6.9 mg, 0.015 mmol, 42%).

^{1}H NMR (500 MHz, DMSO-d$_6$): δ = 12.41 (s, 1H), 8.63 (s, 1H), 8.01 (s, 1H), 7.94-7.98 (m, 2H), 7.93 (d, J = 8.5 Hz, 2H), 7.88 (t, J = 8.1 Hz, 1H), 7.22 (s, 1H), 7.10 (d, J = 8.5 Hz, 2H), 6.81 (s, 2H), 3.84 (s, 3H) ppm.

^{13}C NMR (126 MHz, DMSO-d_6): δ = 169.6, 161.2, 161.0, 158.6, 146.2, 136.6, 135.2, 131.2, 130.3, 128.8, 127.6, 126.1, 121.59, 121.57, 120.2, 119.2, 114.7, 95.7, 55.4 ppm.

^{19}F NMR (470 MHz, DMSO-d$_6$): δ = -61.12 ppm.

HRMS (ESI$^+$): m/z calcd. for $C_{21}H_{17}F_3N_7O_3^+$ [M+H]$^+$ 472.1339, measured 472.1325.

(E)-5-(2-(5-Chloro-2-hydroxybenzylidene)hydrazine-1-carbonyl)-N-(3,4-dimethoxyphenyl)-1-methyl-1H-pyrazole-3-carboxamide (**H1**):

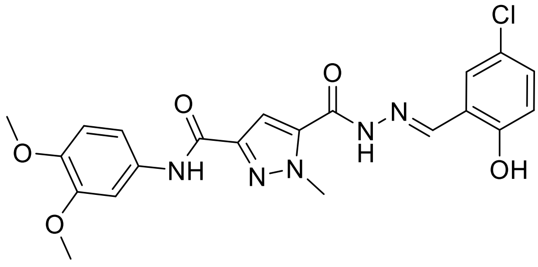

Compound **H1** was synthesised according to GP-3, hydrazide **H** (27.8 mg, 0.087 mmol) and aldehyde **1** (24.0 mg, 0.153 mmol) stirred in MeOH (4 mL) for 3 h to afford compound **H1** as pale yellow solid (37.3 mg, 0.086 mmol, 94%).

^{1}H NMR (500 MHz, DMSO-d_6) δ = 12.28 (br s, 1H), 11.07 (br s, 1H), 10.06 (s, 1H), 8.62 (s, 1H), 7.69 (d, J = 2.4 Hz, 1H), 7.52 (s, 1H), 7.51 (d, J = 2.1 Hz, 1H), 7.43 (dd, J = 8.7, 2.1 Hz, 1H), 7.33 (dd, J = 8.7, 2.4 Hz, 1H), 6.96 (d, J = 8.7 Hz, 1H), 6.91 (d, J = 8.7 Hz, 1H), 4.23 (s, 3H), 3.75 (s, 3H), 3.73 (s, 3H) ppm.

^{13}C NMR (126 MHz, DMSO-d_6) δ = 158.9, 156.0, 155.2, 148.5, 146.1, 145.2, 145.0, 135.4, 132.2, 131.1, 127.2, 123.1, 120.8, 118.3, 112.2, 111.9, 108.8, 105.6, 55.7, 55.4, 39.8 ppm.

HRMS (ESI$^+$): m/z calcd. for $C_{21}H_{21}ClN_5O_5^+$ [M+H]$^+$ 458.1226, measured 458.1198.

(1r,3s,5R,7S)-3-(3-Chloro-1H-1,2,4-triazol-1-yl)-N'-((Z)-5-chloro-2-hydroxybenzylidene)adamantane-1-carbohydrazide (**I1**):

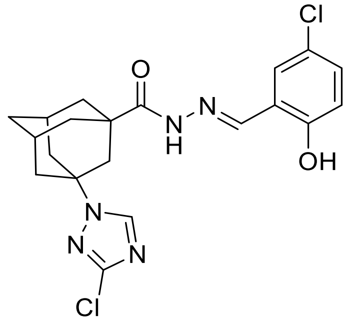

Compound **I1** was synthesised according to GP-4, hydrazide **I** (14.5 mg, 0.049 mmol) and aldehyde **1** (7.9 mg, 0.050 mmol) stirred in MeOH (1 mL) for 2 h to afford compound **I1** as off-white solid (6.5 mg, 0.015mmol, 31%).

^{1}H NMR (500 MHz, DMSO-d_6): δ = 11.35 (s, 1H), 11.28 (s, 1H), 8.70 (s, 1H), 8.52 (s, 1H), 7.59 (d, J = 2.6 Hz, 2H), 7.29 (dd, J = 8.8, 2.6 Hz, 2H), 6.92 (d, J = 8.8 Hz, 1H), 2.32 (br s, 1H), 2.25 (s, 1H), 2.13 (d, J = 12.0 Hz, 2H), 2.07 (d, J = 12.0 Hz, 2H), 1.93 (d, J = 11.6 Hz, 2H), 1.87 (d, J = 11.6 Hz, 2H), 1.70 (br s, 2H) ppm.

^{13}C NMR (126 MHz, DMSO-d$_6$): δ = 171.6, 156.0, 150.2, 145.2, 142.9, 130.6, 127.6, 122.9, 120.6, 118.2, 59.6, 42.4, 41.8, 40.4, 36.8, 34.2, 28.6 ppm.

HRMS (ESI$^+$): m/z calcd. for $C_{20}H_{22}Cl_2N_5O_2^+$ ([M+H]$^+$) 434.1145, measured 434.1135.

(E)-3-Chloro-N'-(5-chloro-2-hydroxybenzylidene)benzohydrazide (**K1**):

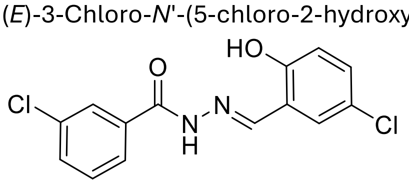

Compound **K1** was synthesised according to GP-3, hydrazide **K** (85.0 mg, 0.498 mmol) and aldehyde **1** (80.0 mg, 0.511 mmol) stirred in MeOH (5 mL) for 2h to afford compound **K1** as off-white solid (154.0 mg, 0.498 mmol, >99%). HPLC-MS indicates the presence of two isomers, possibly *trans* and *cis* conformers of the amide. We did not assign the peaks to a specific conformer and will refer to them as A and B (A:B = 93:3).

^{1}H NMR (400 MHz, DMSO-d_6) δ = 12.24 (s, 1H), 11.19 (br s, 1H), 8.63 (s, 1H), 7.98 (t, J = 1.6 Hz, 1H), 7.90 (dt, J = 7.8, 1.6 Hz, 1H), 7.66-7.70 (m, 2H), 7.58 (t, J = 7.8 Hz, 1H), 7.32 (dd, J = 8.9, 2.7 Hz, 1H), 6.96 (d, J = 8.9 Hz, 1H) ppm.

^{13}C NMR (101 MHz, DMSO-d_6) δ = 161.6, 156.1, 146.1, 134.8, 133.4, 131.9, 131.0, 130.6, 127.4, 126.6, 123.1, 120.7, 118.3, 109.6 ppm.

HRMS (ESI$^+$): m/z calcd. for $C_{14}H_{11}Cl_2N_2O_2^+$ [M+H]$^+$ 309.0192, measured 309.0186.

(E)-3-Chloro-N'-(4-(trifluoromethyl)benzylidene)benzohydrazide (**K4**):

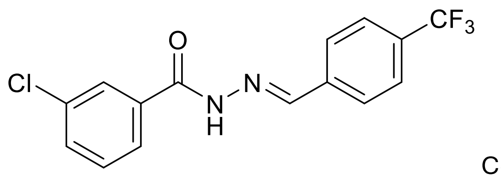

Compound **K4** was synthesised according to GP-3, with some adaptations. Hydrazide **K** (100.0 mg, 0.586 mmol) and aldehyde **4** (103.0 mg, 0.592 mmol, 1 mL MeOH) stirred in MeOH (3 mL) for 1 h. The crude was purified by flash column chromatography (EtOAc:PE, product at 1:1) to afford **K4** as a white powder (162.0 mg, 0.496 mmol, 85%).

^{1}H NMR (400 MHz, DMSO-d_6) δ = 12.12 (br s, 1H), 8.52 (s, 1H), 7.93-7.99 (m, 3H), 7.89 (br d, J = 7.8 Hz, 1H), 7.81 (br d, J = 8.2 Hz, 2H), 7.68 (d, J = 7.8 Hz, 1H), 7.58 (t, J = 7.8 Hz, 1H) ppm.

^{13}C NMR (126 MHz, DMSO-d_6) δ = 161.9, 146.6, 138.2, 135.2, 133.3, 131.8, 130.6, 129.8, 127.8, 127.4, 126.6, 125.8, 124.1 ppm.

^{19}F NMR (470 MHz, DMSO-d_6) δ = -61.20 ppm.

HRMS (ESI$^+$): *m/z* calcd. for $C_{15}H_{11}ClF_3N_2O^+$ [M+H]$^+$ 327.0507, measured 327.0493.

(E)-N'-(5-Chloro-2-hydroxybenzylidene)isonicotinohydrazide (**L1**)

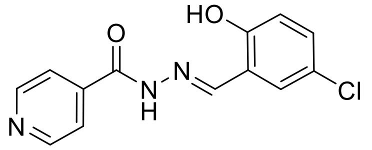

Compound **L1** was synthesised according to GP-3, with some adaptations. Hydrazide **L** (53.2 mg, 0.388 mmol) and aldehyde **1** (61.3 mg, 0.392 mmol) were stirred at reflux in MeOH (2 mL) for 5 h and at rt overnight. Compound **L1** was afforded as pale yellow solid (105.2 mg, 0.382mmol, 98%).

^{1}H NMR (400 MHz, DMSO-d_6) δ = 12.35 (s, 1H), 11.11 (s, 1H), 8.80 (d, J = 6.2 Hz, 2H), 8.66 (s, 1H), 7.84 (d, J = 6.2 Hz, 2H), 7.70 (d, J = 2.5 Hz, 1H), 7.33 (dd, J = 8.6, 2.5 Hz, 1H), 6.96 (d, J = 8.6 Hz, 1H) ppm.

^{13}C NMR (101 MHz, DMSO-d_6) δ = 161.5, 156.1, 150.4, 146.5, 139.9, 131.1, 127.2, 123.1, 121.5, 120.7, 118.3 ppm.

HRMS (ESI$^+$): *m/z* calcd. for $C_{13}H_{11}ClN_3O_2^+$ [M+H]$^+$ 276.0534, measured 276.0526.

*Mtb*IspE expression and purification

The synthetic gene encoding for *Mtb*IspE was purchased from GenScript, GmbH cloned inside the plasmid pvp008, downstream a *N*-terminal StrepII tag followed by a TEV site. The plasmid was transformed into RB-competent *E. coli* Arctic express (DE3). Transformed *E. coli* cells were grown in LB medium at 37 °C until OD_{600} reached 0.6; after that, protein expression was induced adding 0.5 mM IPTG and the protein was expressed at 16°C for 48 hours. Cells were harvested by centrifugation at 5000 rpm for 20 minutes. The pellet was resuspended in wash buffer (50 mM HEPES pH 7, 100 mM NaCl, 5 mM $MgCl_2$, 5 mM DTT) and sonicated for cell disruption. The lysate was cleared by centrifugation for 45 minutes at 16000 rpm at 4 °C. The supernatant was syringe-filtered and loaded onto a self-packed StreptActin-HC column equilibrated in wash buffer. *Mtb*IspE was eluted with a single step 40 mL gradient at 100% elution buffer (50 mM HEPES pH 7, 100 mM NaCl, 5 mM $MgCl_2$, 5 mM DTT, 5 mM desthiobiotin). Protein purity was verified via SDS-PAGE. The purification yield was 2 mg/L.

*Mtb*IspE stability study *via* thermal shift assay

The stability of *Mtb*IspE in the DCL buffer conditions was studied for 10 days by measuring its melting temperature (T_m) using thermal shift assay (TSA). *Mtb*IspE incubated at a final concentration of 0.2 mg/mL in Tris buffer at pH 7.0 with 5%,-and 10% DMSO at room temperature and samples were measured in duplicate at t = 0, 1, 2, 6, 8 & 10 days (10% DMSO sampling stopped after day 6). The experiments were performed in a 96-well PCR plate (Thermoscientific). The final volume per well was 20 µL, consisting of 18 µL of *Mtb*IspE sample (as mentioned above), and 2 µL of GloMelt dye (diluted 20x with H_2O). The plate was centrifuged for 1 minute at room temperature at 1200 rpm. The T_m of the protein was measured using a Real-time PCR machine (Step one plus, Applied Biosystem). The conditions of the experiment were adjusted using Step One 2.3 software. The starting temperature, the ending temperature and the heating rate were set as 21 °C, 95 °C and 0.5 °C / min, respectively. The melting curves were analysed using Protein

Thermal Shift 1.3 software. T_m of *Mtb*IspE under these conditions was found to range from 50.4 °C to 51.3 °C, with one duplicate of day 6 (5% DMSO sample) discarded due to anomalies in the measurement. As expected *Mtb*IspE is more stable and has higher T_m at 5% DMSO than 10% DMSO. The T_m of *Mtb*IspE with 5% DMSO steadily declines from day 6 (Figure S1)

In silico elucidation of tdDCC hits' binding mode

Docking studies were performed using a pre-release of SeeSAR 13.1 (BioSolveIT, GmbH, Sankt Augustin, Germany), provided for beta-test by the company. Two published *Mtb*IspE crystal structures in complex with ADP and CDP-ME (PDB accession code: 3PYF and 3PYE respectively) were superimposed and the 47 amino-acid in the binding region of both ADP and CDP-ME were used to define active site on 3PYF structure. All 72 possible product combinations (DCL-1, -2,-3, and-4) were docked on the active site defined as described above generating a maximum of 10 poses per molecule using standard docking parameters of SeeSAR software. The 720 poses generated were clustered by fragment and filtered basing on calculated torsion quality and molecular clashes. The binding pose of each fragment in the different molecules was visually analysed to determine whether a tendency in predicted binding pose of such fragments was found (Figure S10 –S12,p. 11)

Kinetic turbidimetric solubility

The desired compounds were sequentially diluted in DMSO in a 96-well plate. 7.5 µL of each well were transferred into another 96-well plate and mixed with 142.5 µL of PBS. Plates were shaken for 5 min at 600 rpm at room temperature (r.t.), and the absorbance at 620 nm was measured. Absorbance values were normalized by blank subtraction and plotted using GraphPad Prism 8.4.2 (GraphPad Software, San Diego, CA, USA).. Solubility (S) was quantified as the concentration leading to an absorbance value of 0.005 via determining the 'First X' value of the AUC function using a threshold of 0.005."

*Mtb*IspE binding study *via* MicroScale Thermophoresis

*Mtb*IspE buffer was exchanged to 50 mM HEPES pH 7, 100 mM NaCl, 5 mM $MgCl_2$, 5 mM TCEP before protein labelling. The enzyme was labelled with Cy5 following the protocol indicated by NanoTemper. Labelled *Mtb*IspE was diluted to 400 nM and used mixed 1:1 with 40 µM of each ligand. MicroScale Thermophoresis experiment was performed with a Monolith N.115 (Nanotemper Technologies, GmbH, Munich, Germany) using Standard Capillaries (Nanotemper Technologies, GmbH, Munich, Germany). Each binding event was evaluated at a final concentration of 200 nM *Mtb*IspE and 20 µM of each ligand. All the measurements were performed in triplicates. Data analysis was automatically performed by Monolith NT.115 Control and Analysis software (Nanotemper Technologies, GmbH, Munich, Germany).

*Mtb*IspE binding study *via* thermal shift assay

The shifts in T_m of *Mtb*IspE induced by the compounds were determined *via* TSA. *Mtb*IspE (15 µM) was incubated with 20 µM of each compound for 30 min in 50 mM HEPES (pH 7.0, 100 mM NaCl, 5 mM $MgCl_2$, 5 mM DTT). After that, SYPRO ORANGE fluorescent dye (Thermo Fisher) was added at a final concentration of 5X. Melting curves of each sample was measured increasing the temperature from 10 °C to 95 °C with an increase of 1 °C/30 sec and recording fluorescence of SYPRO ORANGE after every temperature increase using CFX96 real-time system-C1000 Thermal Cycler (Bio-Rad). Melting temperature (Tm) of each sample were calculated using CFX manager 2.0 software (Bio-Rad). Data analysis and visualization was performed using Excel and GraphPad 9.8.

Determination of *E.coli* activity

Growth inhibition values were determined in 96-well plates (Sarstedt, Nümbrecht, Germany) against *Escherichia coli* K12 and *E. coli ΔtolC*. As bacteria start OD_{600} 0.03 was used in a total volume of 200 µL in lysogeny broth (LB) medium containing the compounds dissolved in DMSO (DMSO concentration in the experiment: 1%) at a concentration of 50 µM. The OD values were measured using a CLARIOstar platereader (BMG labtech, Ortenberg, Germany) after inoculation and after incubation for 18 h at 37 °C with 50 rpm. Based on these values, percent inhibition values were calculated in relation to the DMSO control.

Determination of *in vitro* anti-tubercular activity and solubility in 7H9 medium

Anti-tubercular tests were performed as previously described.[1] In brief, 7H9 complete medium (BD Difco; Becton Dickinson, Maryland,USA) supplemented with 10% OADC (BD), 0.2% glycerol, and 0.05% Tween80 as previously described,[3] was used to culture *Mycobacterium tuberculosis* (*Mtb*) strain H37Rv (ATCC 25618) carrying a mCherry-expressing plasmid (pCherry10).[4] Cultures were harvested at mid-log phase and frozen in aliquots at – 80 °C. Prior to testing aliquots were thawed followed by centrifugation and the pellet was resuspended in 7H9 medium with 10% OADC (without glycerol and Tween80). This was further thoroughly resuspended by passing it through a syringe with a 26-gauge needle to avoid clumping of the bacteria. 2×105 CFU (colony forming units) were then cultured in a total volume of 100 µl culture medium (triplicates) to test the non-precipitating compounds for the anti-tubercular activity at the concentrations indicated. For these assays, 96-well flat clear bottom black polystyrene microplates (Corning® CellBIND®, Merck, New York, USA) were used. Each plate had Rifampicin (at 1 µg/ml and 0.1 µg/ml) (National Reference Center, Borstel) as a reference compound. Plates were sealed with an air-permeable membrane (Porvair Sciences, Wrexham, UK) in a 37 °C incubator with mild agitation (TiMix5, Edmund Bühler, Germany). The activity of compounds was determined after 7 days by measuring the bacterial growth as relative light units (RLU) from the fluorescence intensity obtained at an excitation wavelength of 575 nm and an emission wavelength of 635 nm in a microplate reader (Synergy 2, BioTek Instruments, Vermont, USA). Two independent experiments (each in triplicates) were performed, and all values were normalized to untreated control sample (100%) in each experiment.

Cytotoxicity assay

To obtain information regarding the toxicity of our compounds, their impact on the viability of human cells was investigated. HepG2 cells (2x104 cells per well) were seeded in 96-well, flat-bottomed culture plates in 100 µL culture medium (DMEM containing 10% fetal calve serum, 1% penicillin-streptomycin). Twenty-four hours after seeding the cells, medium was removed and replaced by medium containing test compounds in a final DMSO concentration of 1%. Compounds were tested in duplicates at a single concentration or, for CC50 determination, at 8 concentrations that were prepared via 2-fold serial dilutions in 1% DMSO/medium. Epirubicin and doxorubicin were used as positive controls in serial dilutions starting from 10 µM, and rifampicin was used as a negative control (at 100 µM). The living cell mass was determined 48 h after treatment with compounds by adding 0.1 volumes of 3-(4,5-dimethylthiazol-2-yl)-2,5-diphenyltetrazolium bromide (MTT) solution (5 mg/mL sterile PBS) (Sigma, St. Louis, MO) to the wells. After incubating the cells for 30 min at 37 °C (atmosphere containing 5% CO2), medium was removed and MTT crystals were dissolved in 75 µL of a solution containing 10% SDS and 0.5% acetic acid in DMSO. The optical density (OD) of the samples was determined photometrically at 570 nm in a PHERAstar Omega plate reader (BMG labtech, Ortenberg, Germany). To obtain percent viability for each sample, their ODs were related to those of DMSO controls. At least two independent measurements were performed for each compound. The calculation of CC50 was performed using the nonlinear regression function of GraphPad Prism 10 (GraphPad Software, San Diego, CA, USA).

Supplementary Figures

Selection of previously published IspE inhibitors

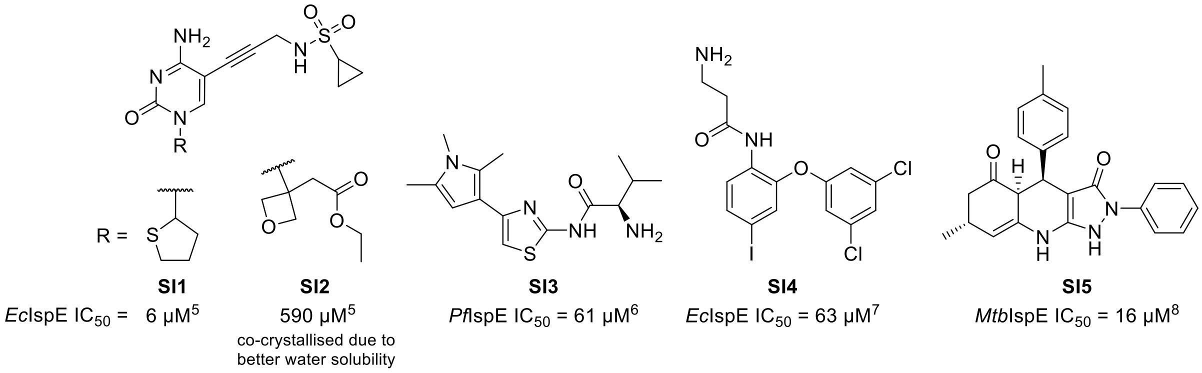

Figure S1. Representative structures of IspE inhibitor classes mentioned in the introduction of the main text.

*Mtb*IspE stability study *via* thermal shift assay

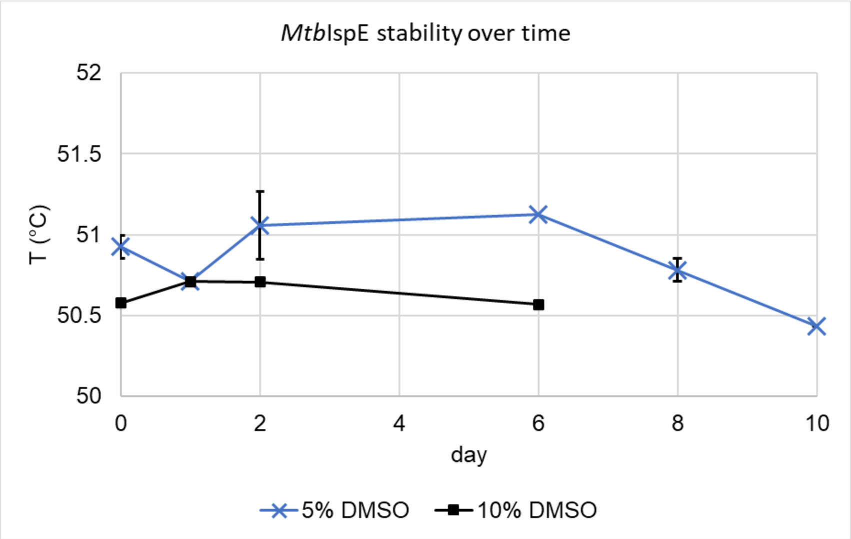

Figure S2. *Mtb*IspE melting temperature evolution over a period of 10 days at pH = 7.0 in Tris buffer with 5%, and 10% DMSO.

HPLC-MS/MS peak assignments and determination of equilibrium

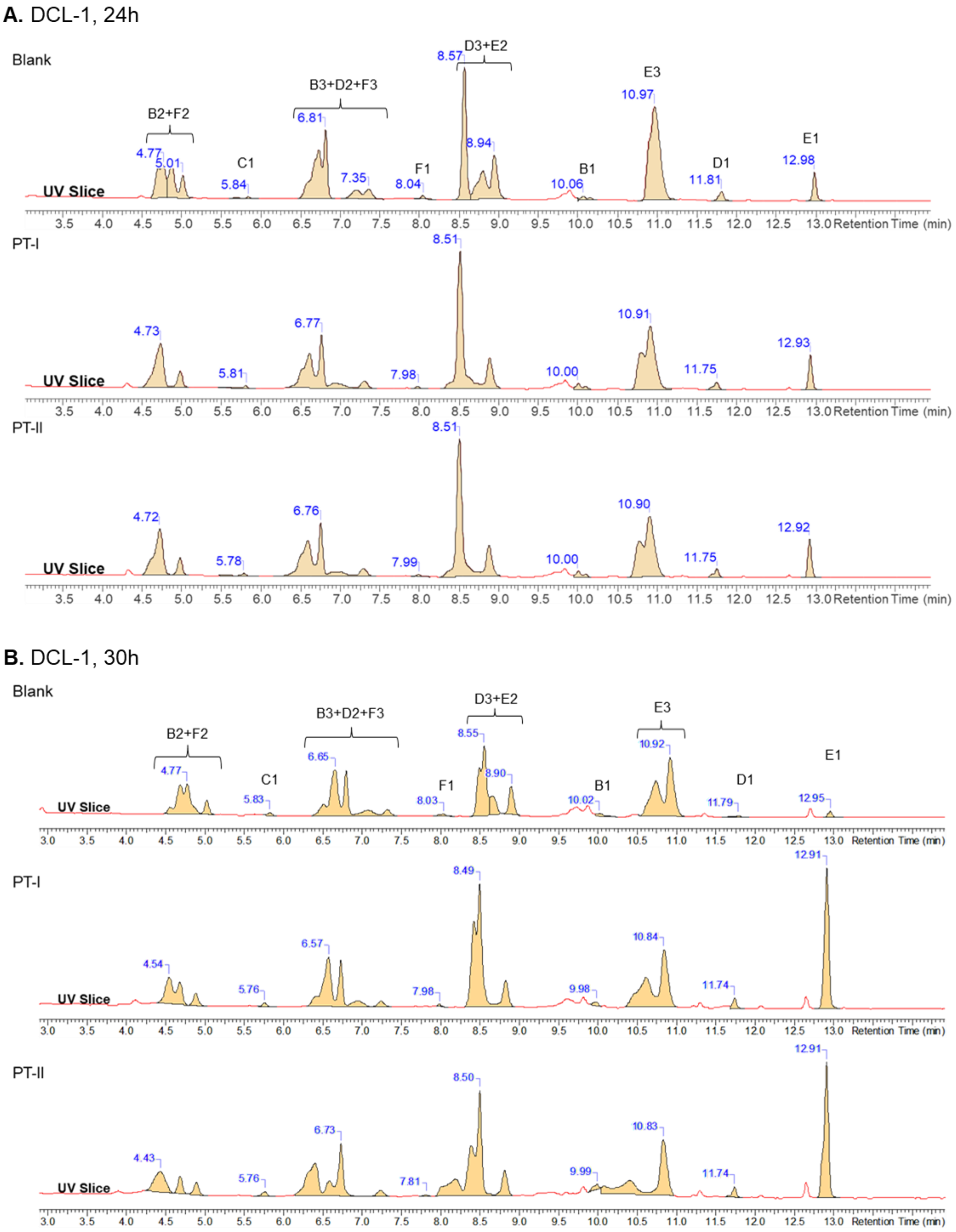

Figure S3. Product peak assignment for tdDCC analysis. HPLC UV absorbance at 310nm of DCL-1 at A) 24 h and B) 30 h of blank and protein-templated duplicates. The following products were not considered: **A1**–**A3** (not found), **C2** (interference with injection peak), **C3** (RPA <1%).

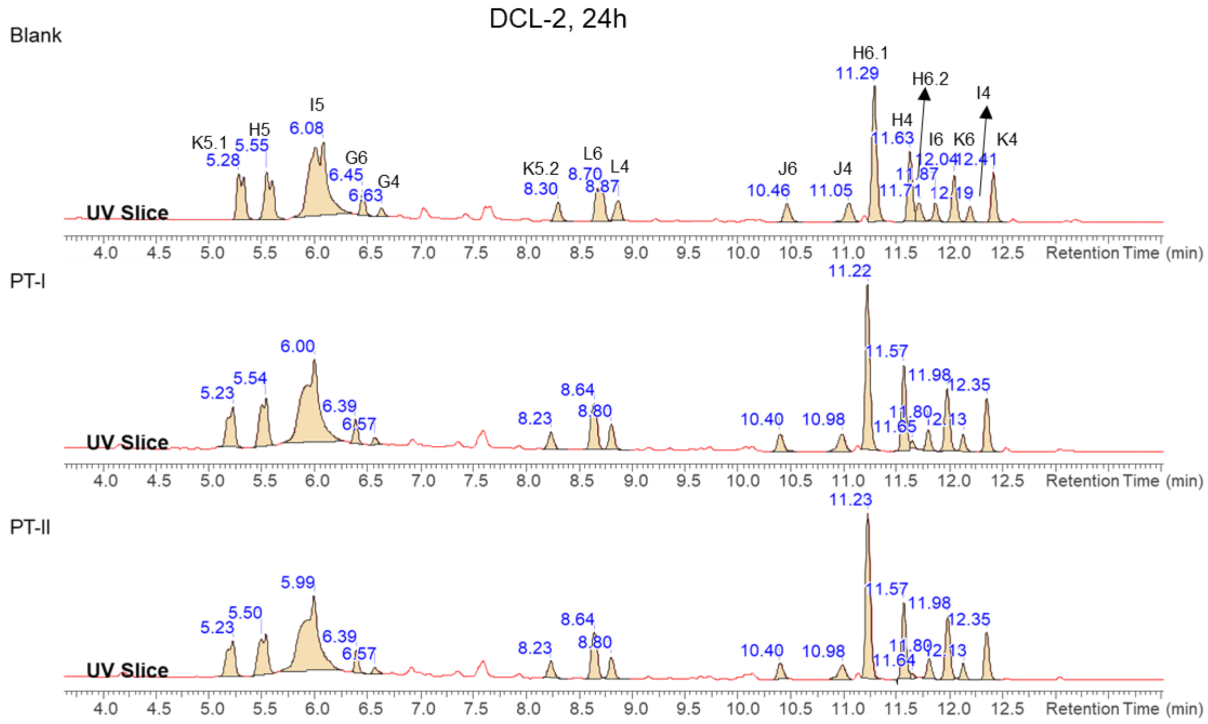

Figure S4. Product peak assignment for tdDCC analysis. HPLC UV absorbance at 310nm of DCL-2 at 24 h of blank and protein-templated duplicates. The following products were not considered: **G5** (interference with injection peak), **L5** and **J5** (RPA <1%).

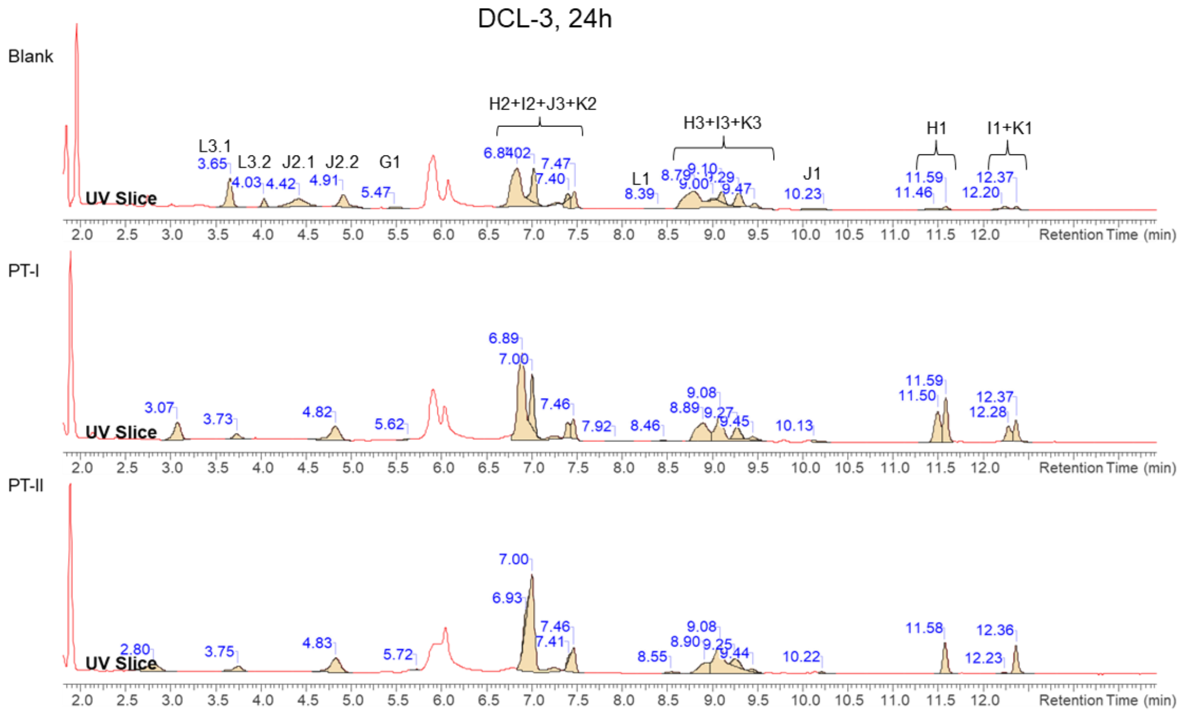

Figure S5. Product peak assignment for tdDCC analysis. HPLC UV absorbance at 310nm of DCL-3 at 24 h of blank and protein-templated duplicates. The following products were not considered: **G2, G3** and **L2** (interference with injection peak).

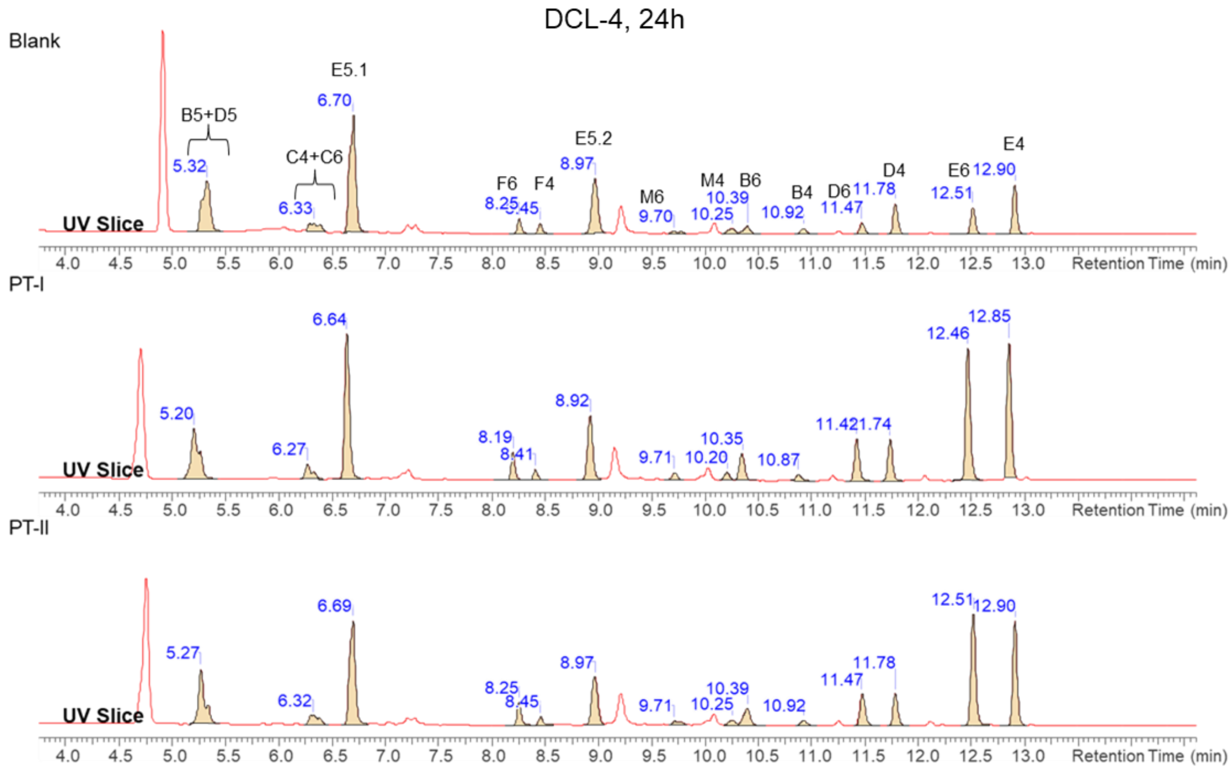

Figure S6. Product peak assignment for tdDCC analysis. HPLC UV absorbance at 310nm of DCL-4 at 24 h of blank and protein-templated duplicates. The following products were not considered: **C5** (interference with injection peak), **F5** (RPA <1%), and **M5** (not found).

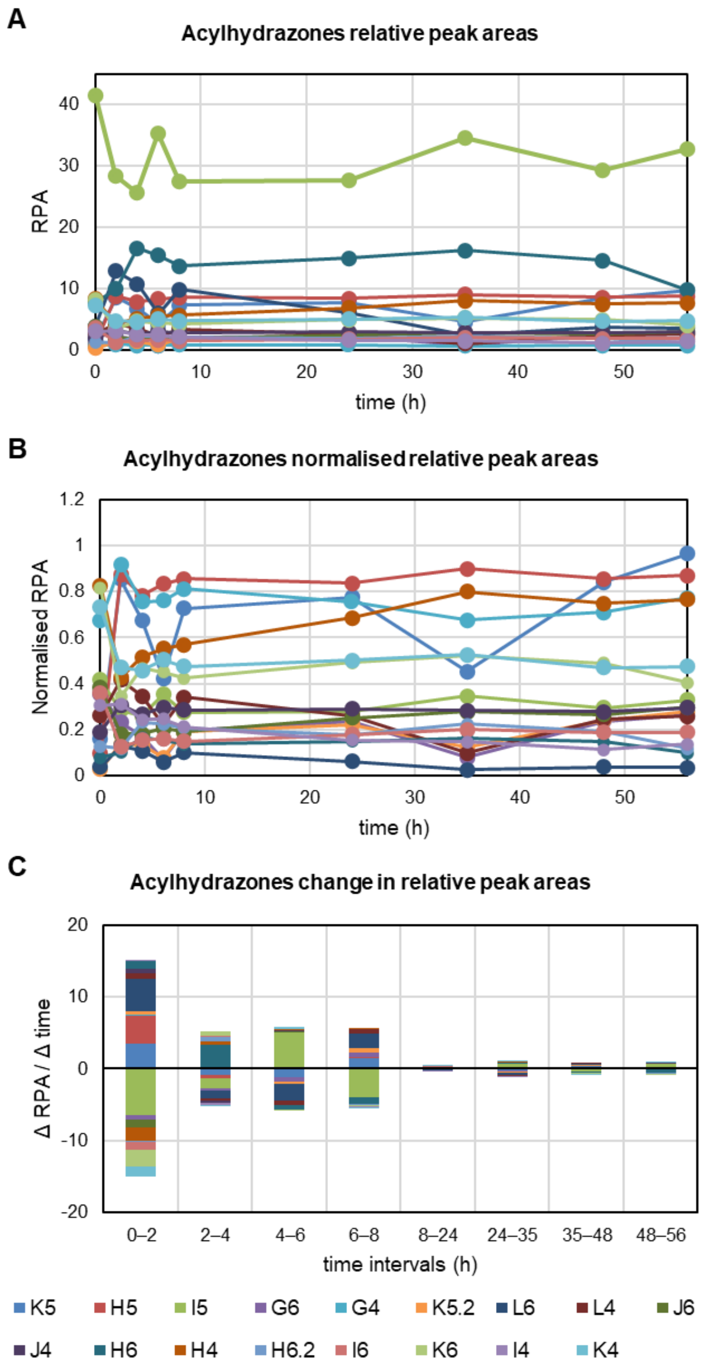

Figure S6. Graphical determination of equilibrium in blank DCL-2. A) Evolution of relative peak areas (RPA) of formed acylhydrazones over time. B) Normalised RPAs over time. C) Change of RPA in each time interval.

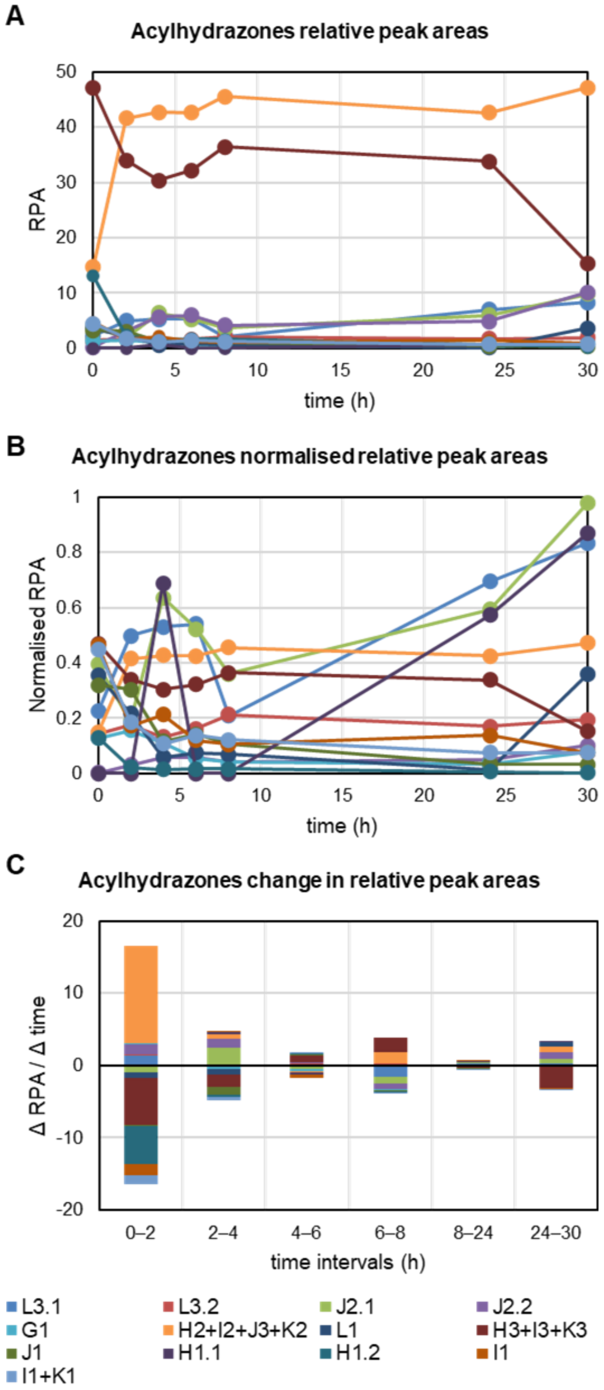

Figure S7. Graphical determination of equilibrium in blank DCL-3. A) Evolution of relative peak areas (RPA) of formed acylhydrazones over time. B) Normalised RPAs over time. C) Change of RPA in each time interval.

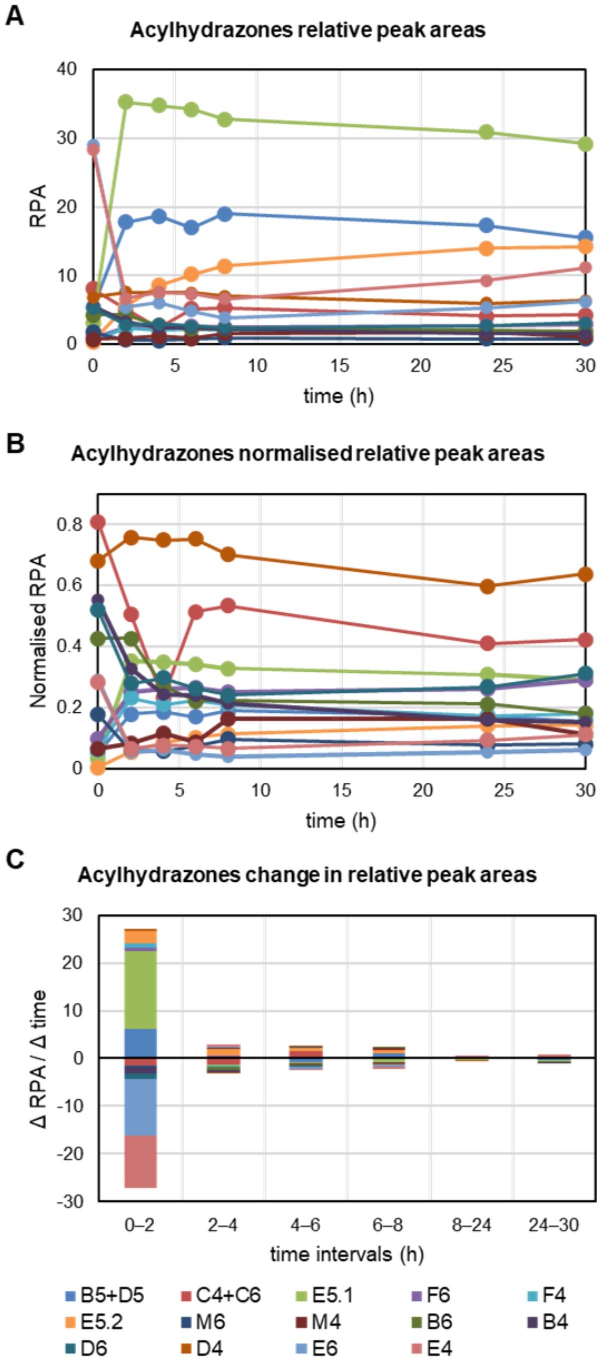

Figure S8. Graphical determination of equilibrium in blank DCL-4. A) Evolution of relative peak areas (RPA) of formed acylhydrazones over time. B) Normalised RPAs over time. C) Change of RPA in each time interval.

Amplification of *N*-acylhydrazones in the PT experiments

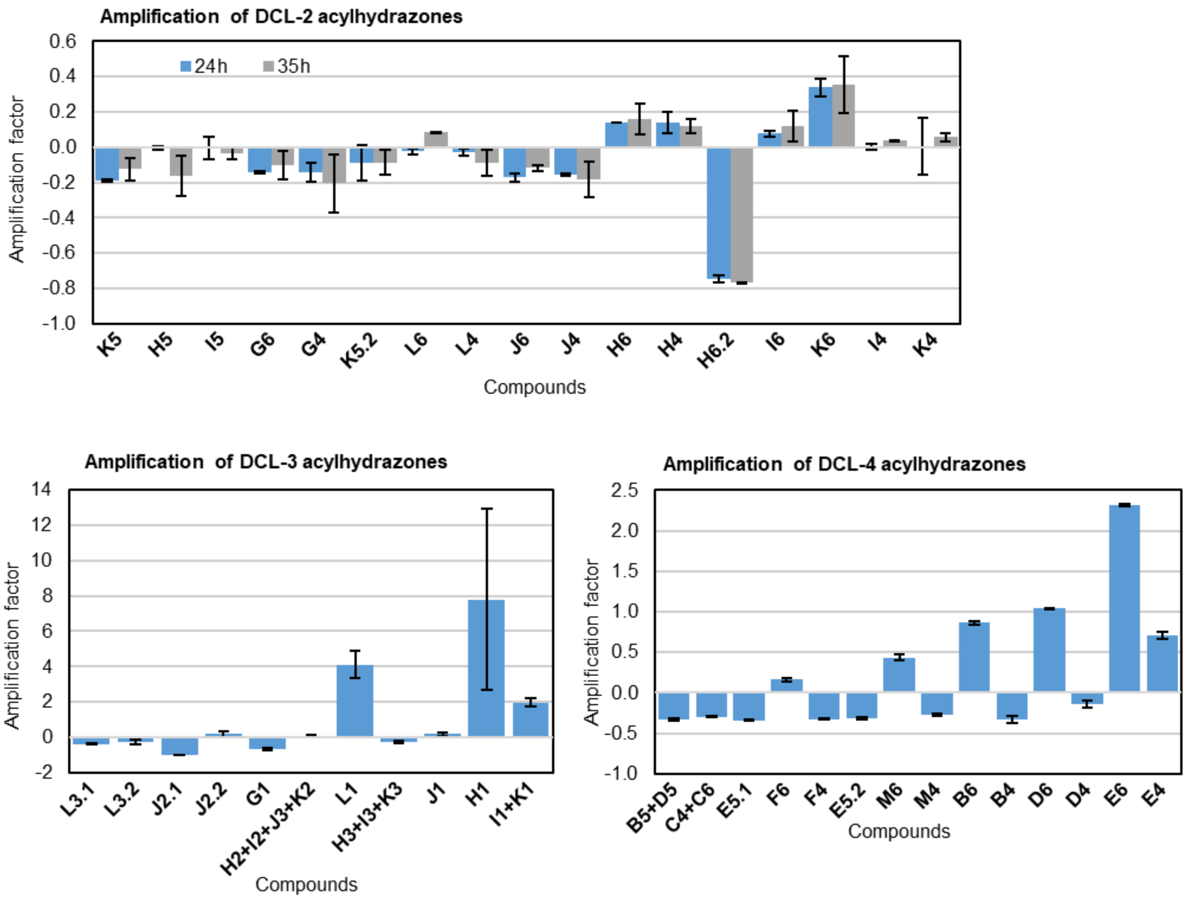

Figure S10. Amplifications of *N*-acylhydrazone products in protein-templated experiments DCL-2,-3, and-4 at 24 h. In the case of DCL-2, also at 35 h.

In silico elucidation of tdDCC hits' binding mode

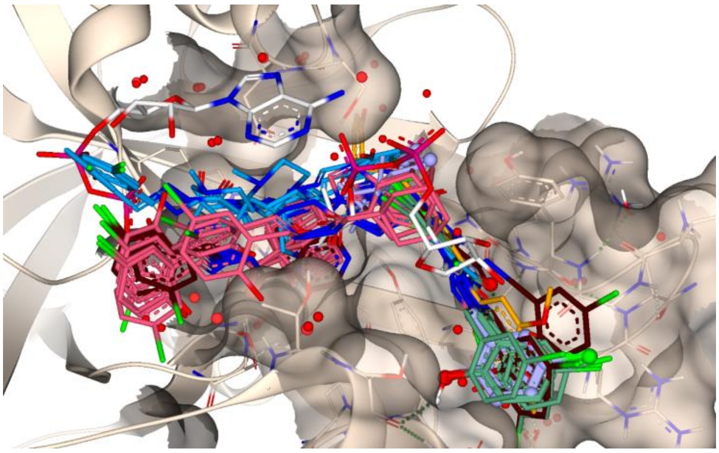

Figure S11. Binding poses of hit-structures containing fragment **1**, natural substrate CDP-ME and co-factor analog ADP. Molecules are represented with the following colours: **C1** in blue; **D1** in violet, **H1** in yellow, **I1** in pink, **K1** in green, **L1** in brown, CDP-ME and ADP in white.

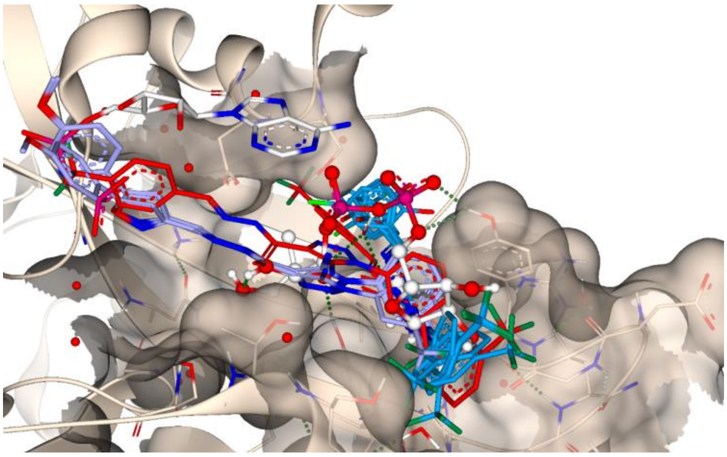

Figure S12. Binding poses of hit-structures containing fragment **E** and , natural substrate CDP-ME and co-factor analog ADP. Molecules are represented with the following colours: **E1** in blue, **E4** in red, **E6** in violet, CDP-ME and ADP in white.

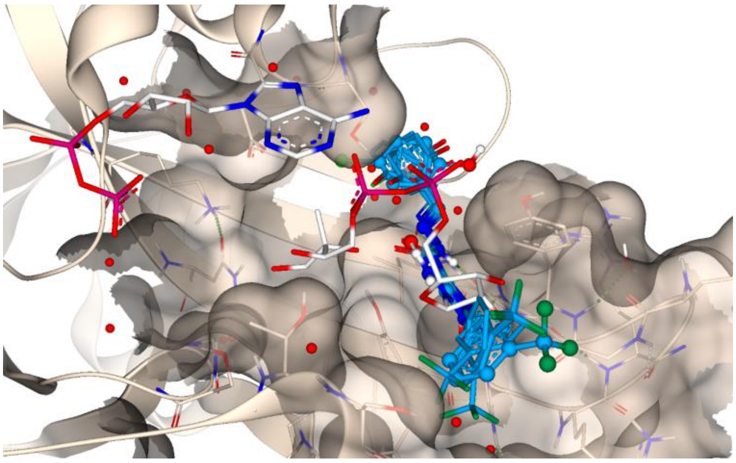

Figure S13. Binding poses of hit-structure **E1** and natural substrates. Molecules are represented with the following colours: **E1** in blue, CDP-ME and ADP in white.

NMR, HRMS, and LCMS spectra of acylhydrazone products

Compound **B6**

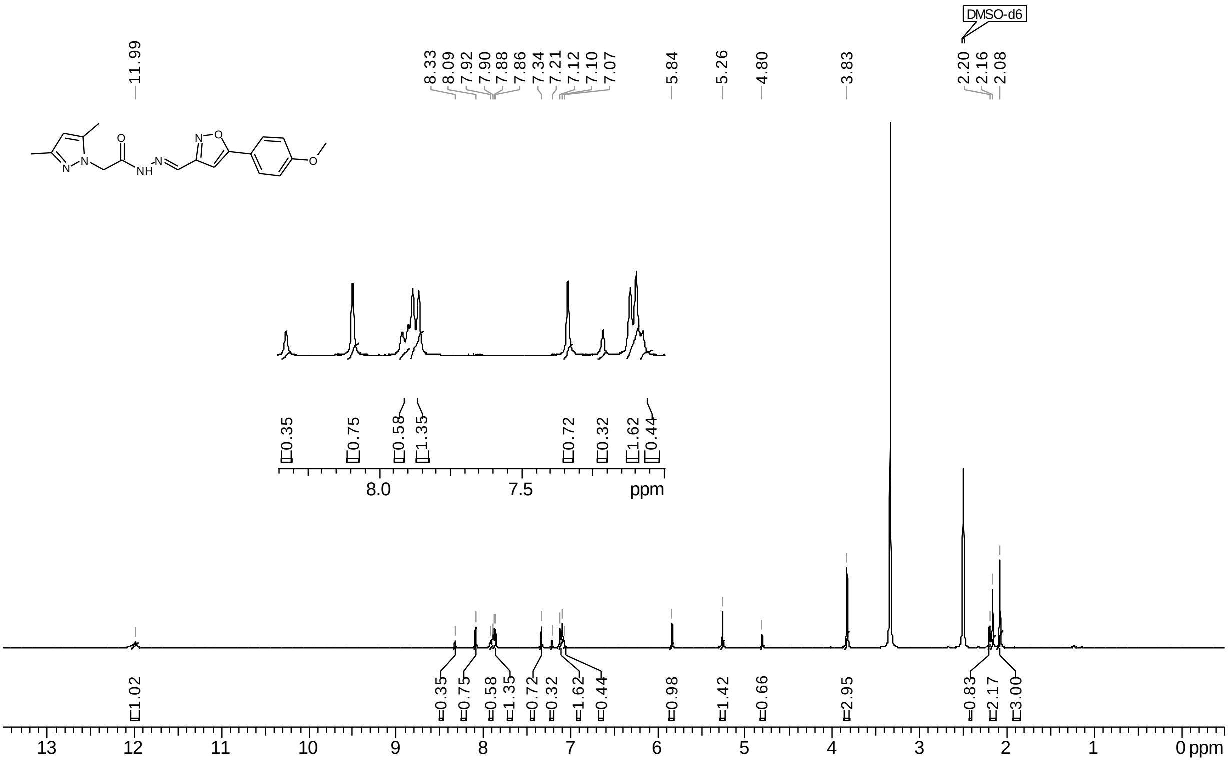

Figure S14. ^{1}H NMR spectrum of **B6**.

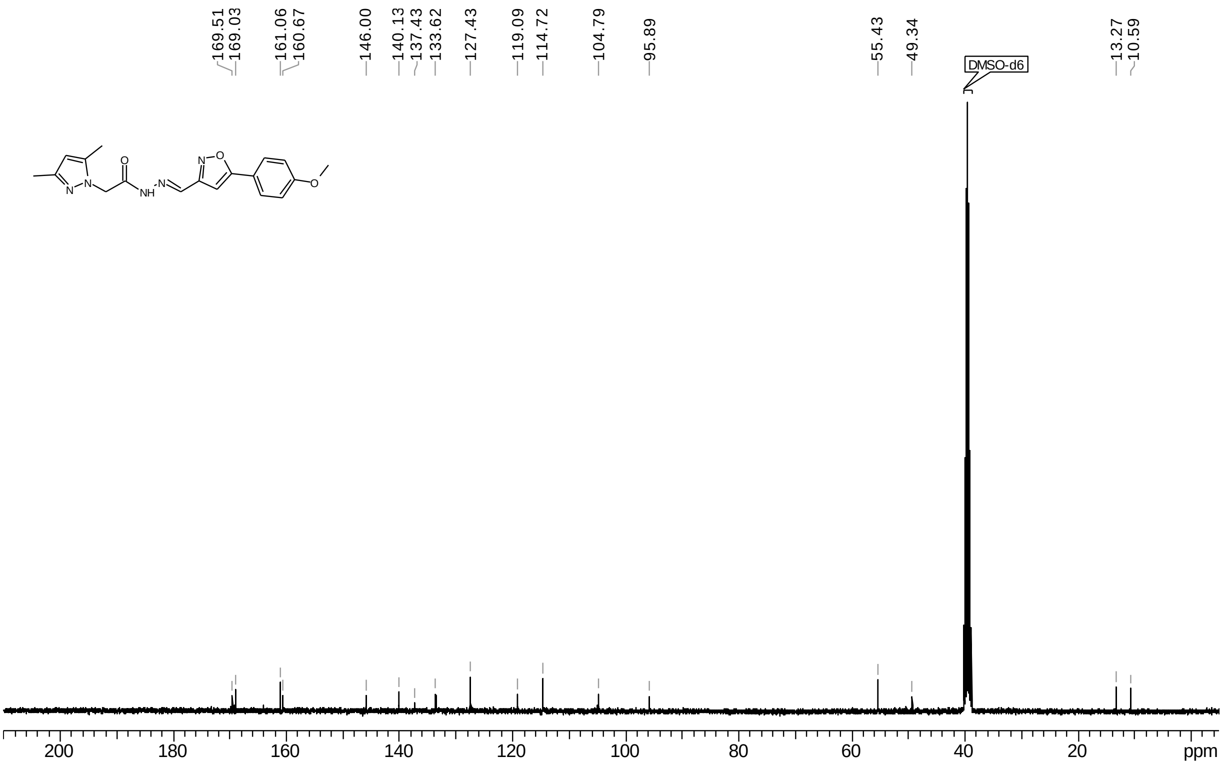

Figure S15. ^{13}C NMR spectrum of **B6**.

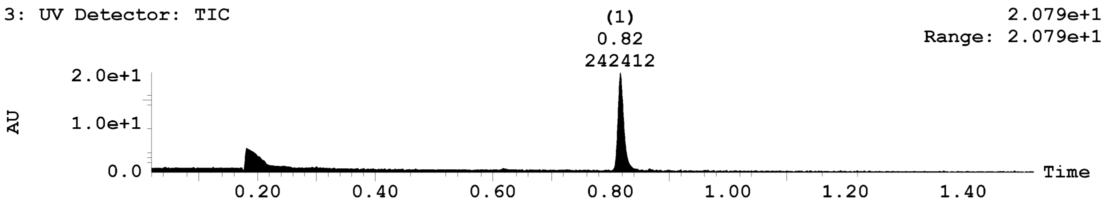

Peak Number	Time	Area %Total
1	0.82	100.00

Figure S16. LCMS purity analysis of **B6**.

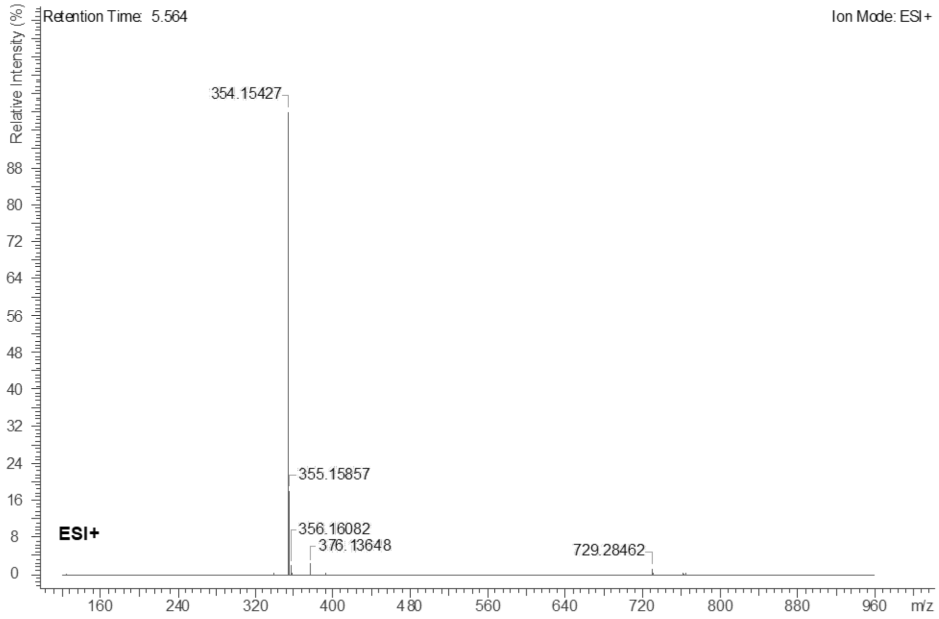

Figure S17. HRMS of **B6**.

Compound **C1**

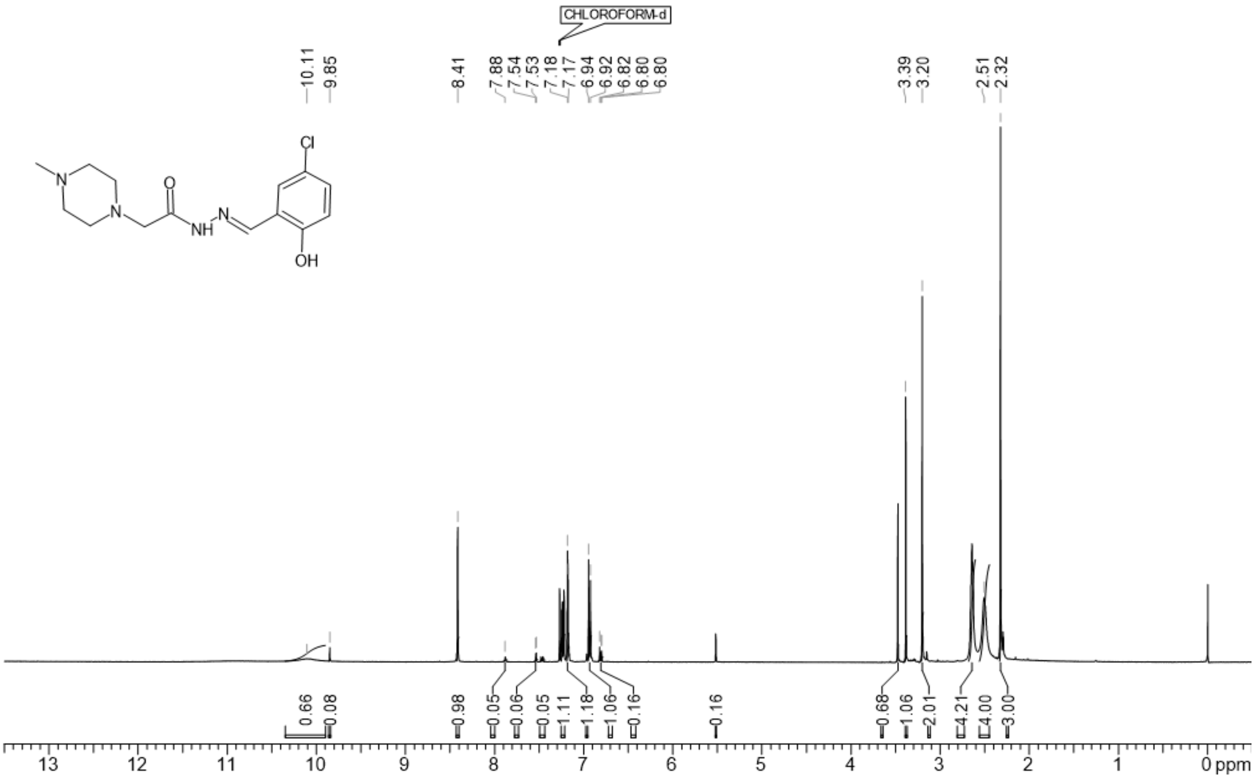

Figure S18. ^{1}H NMR spectrum of **C1**.

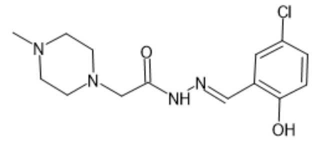

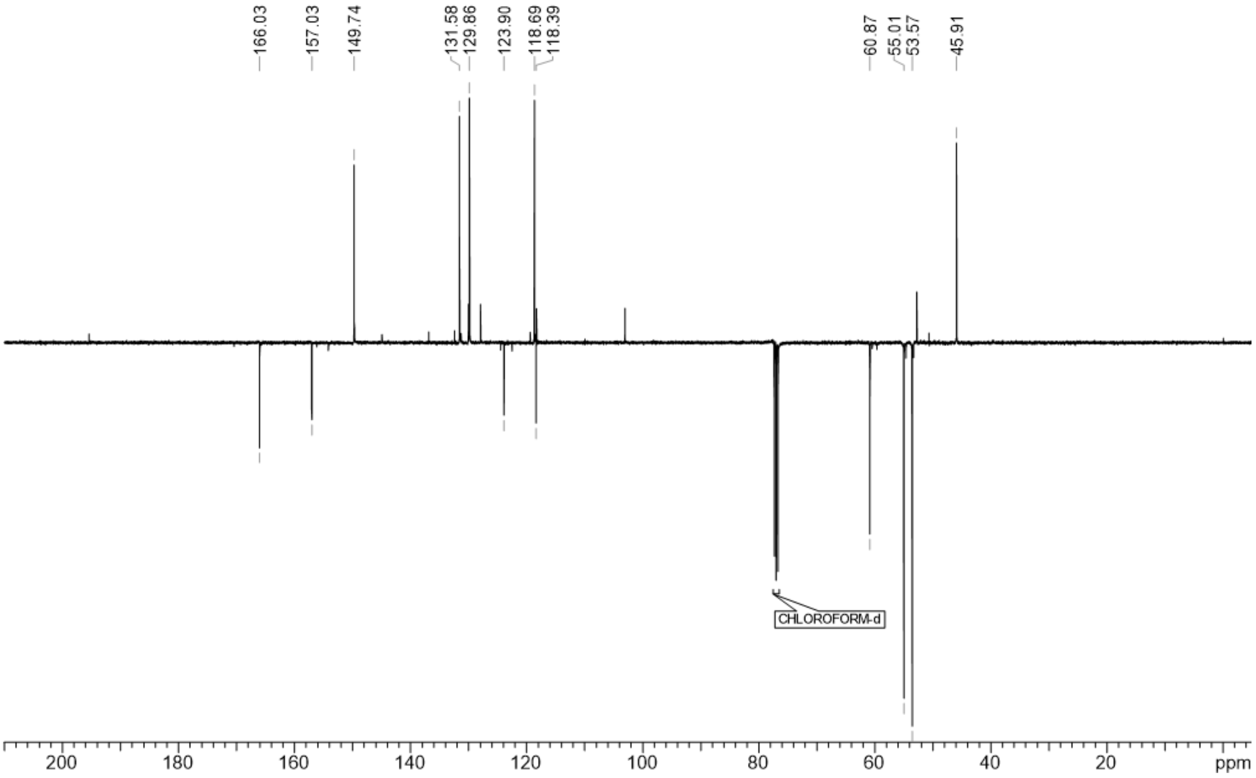

Figure S19. ^{13}C ATP-NMR spectrum of **C1**.

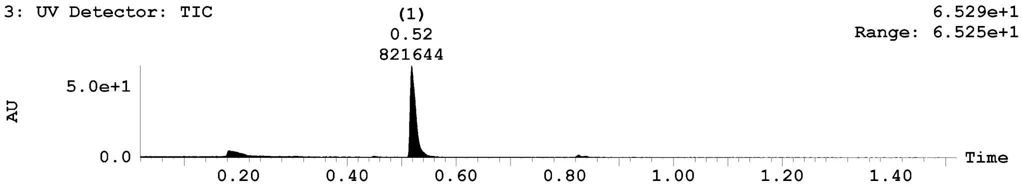

Peak Number	Time	Area %Total
1	0.52	100.00

Figure S20. LCMS purity analysis of **C1**.

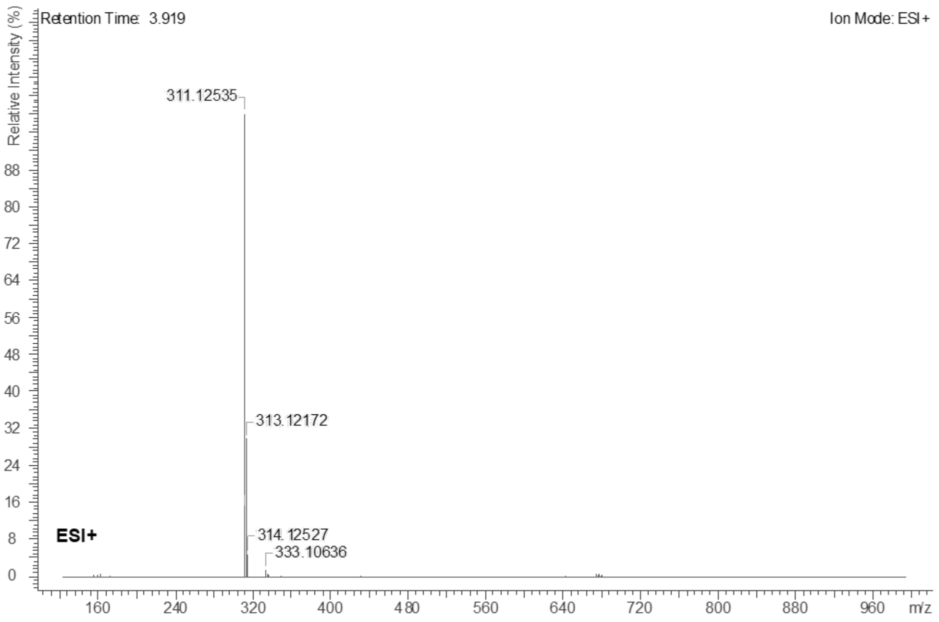

.

Figure S21. HRMS of **C1**.

Compound **D1**

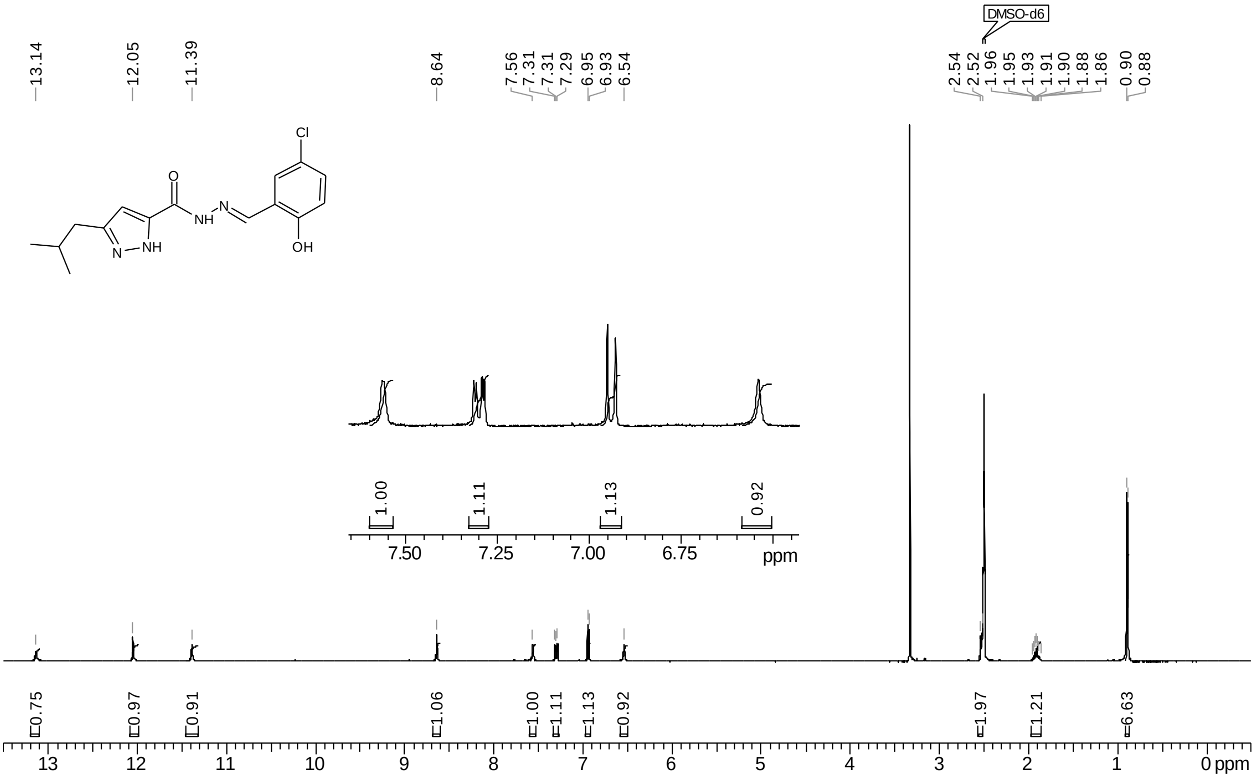

Figure S22. ^{1}H NMR spectrum of **D1**.

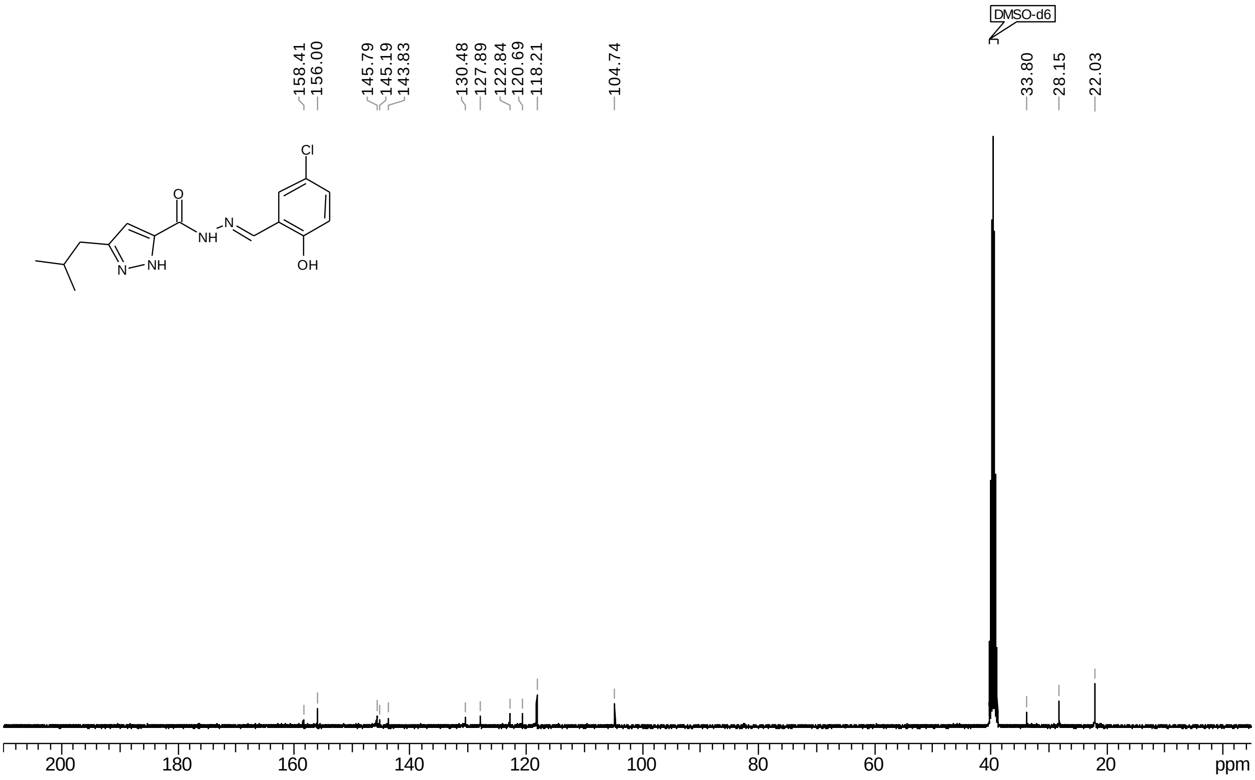

Figure S23. ^{13}C NMR spectrum of **D1**.

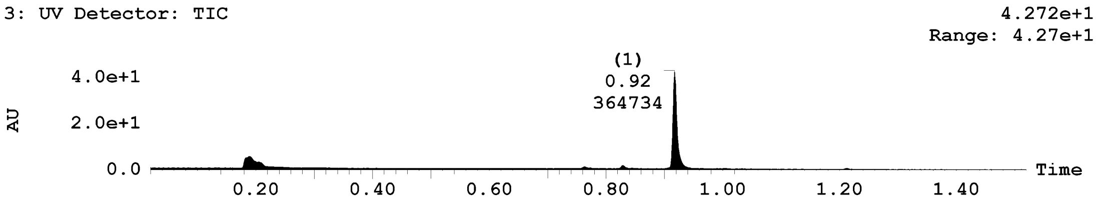

Peak Number	Time	Area %Total
1	0.92	100.00

Figure S24. LCMS purity analysis of **D1**.

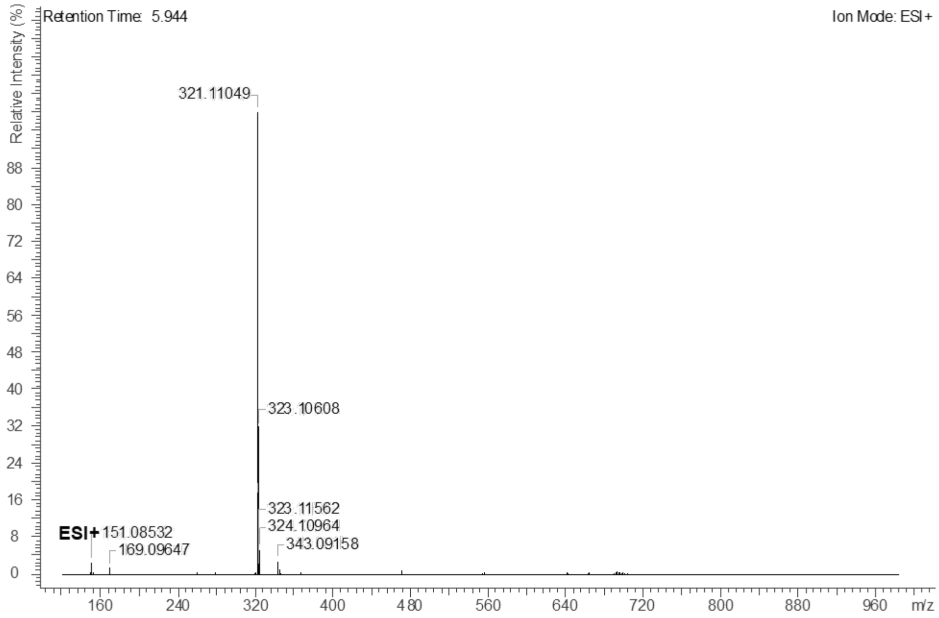

Figure S25. HRMS of **D1**.

Compound **D6**

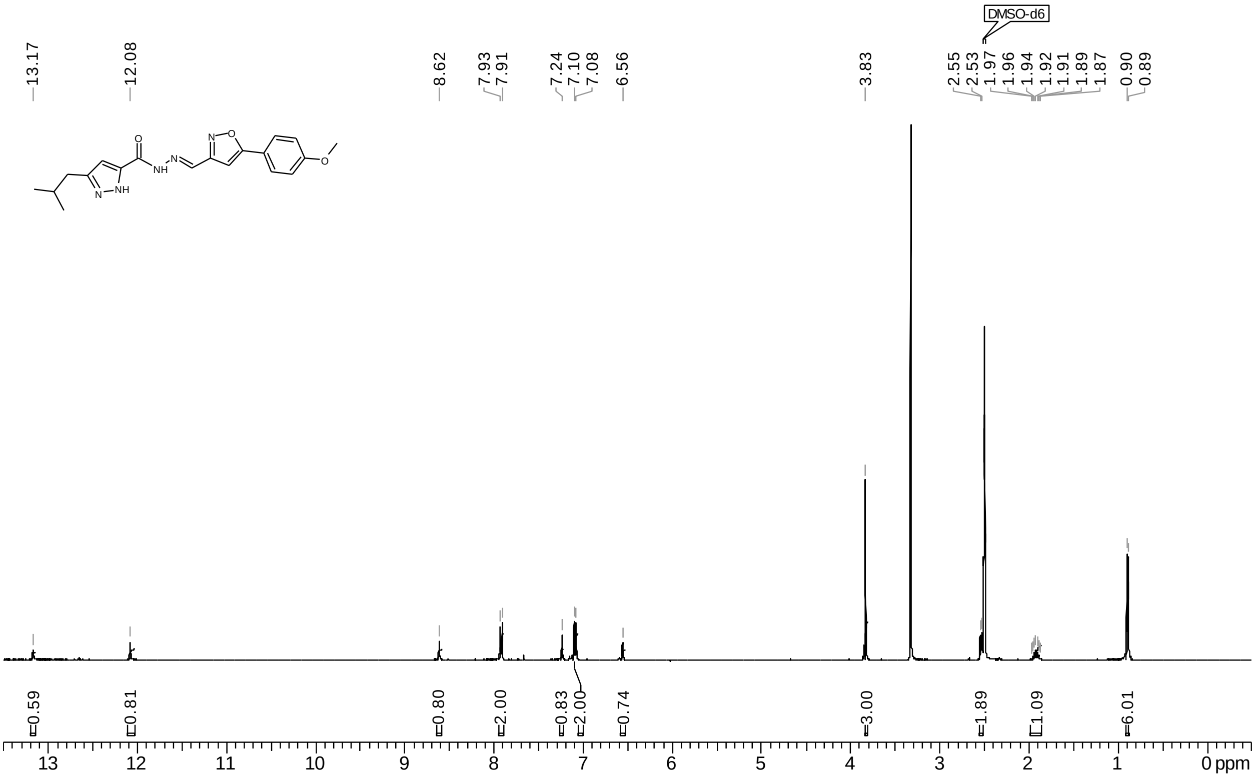

Figure S26. ^{1}H NMR spectrum of **D6**.

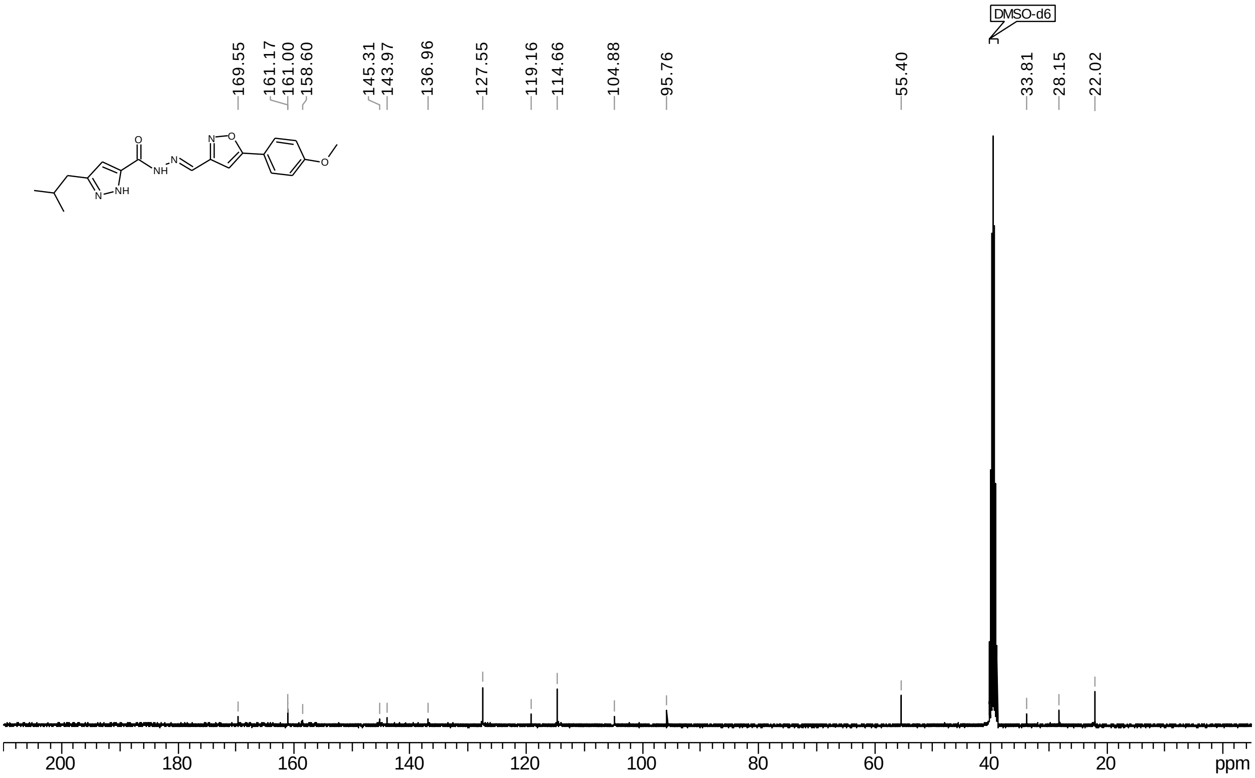

Figure S27. ^{13}C NMR spectrum of **D6**.

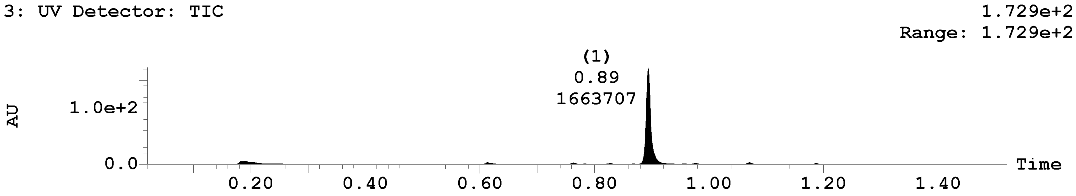

Peak Number	Time	Area %Total
1	0.89	100.00

Figure S28. LCMS purity analysis of **D6**.

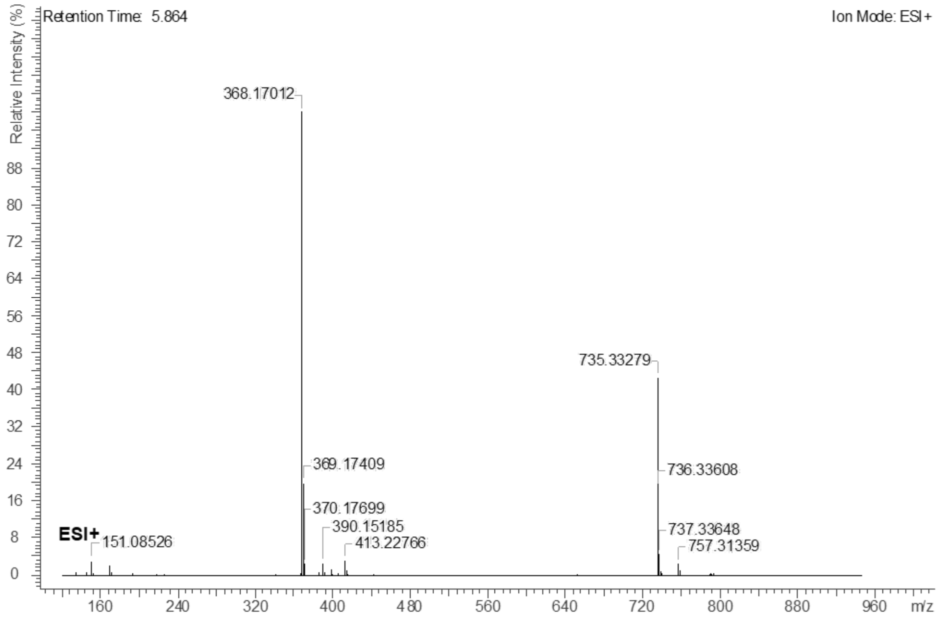

Figure S29. HRMS of **D6**.

Compound **E1**

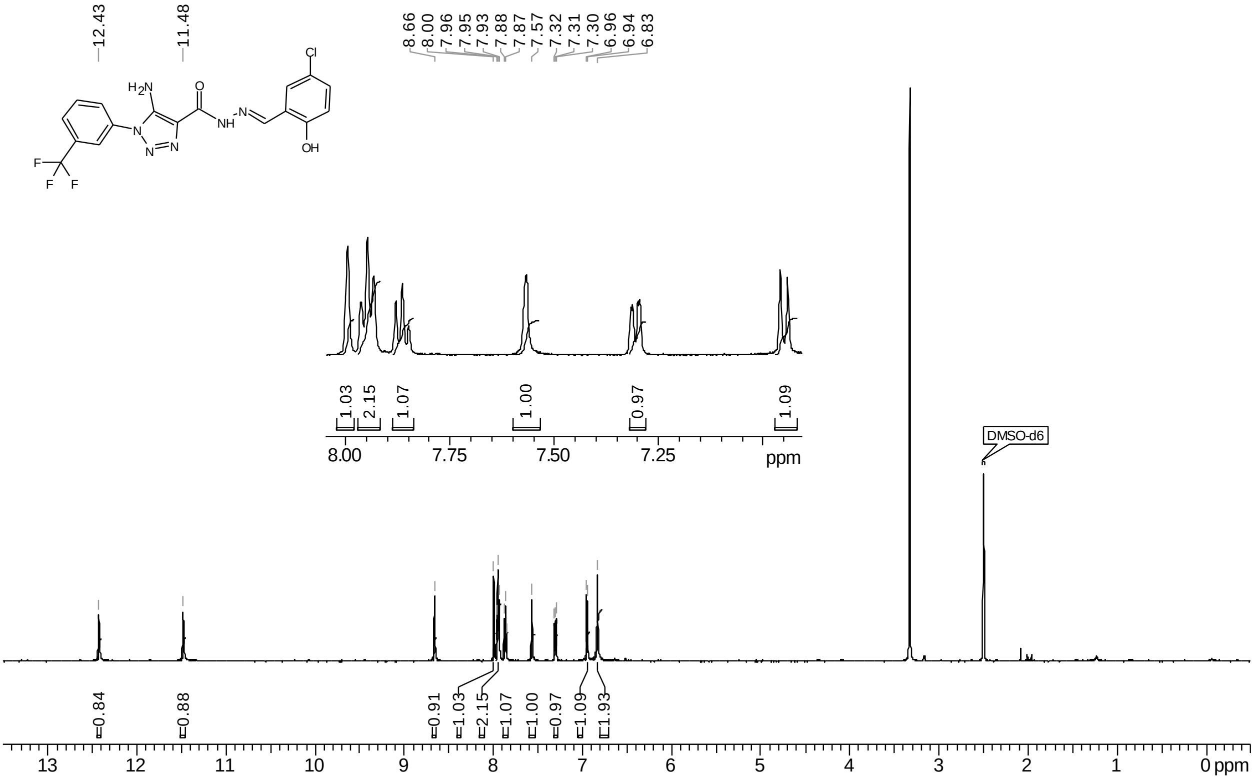

Figure S30. ^{1}H NMR spectrum of **E1**.

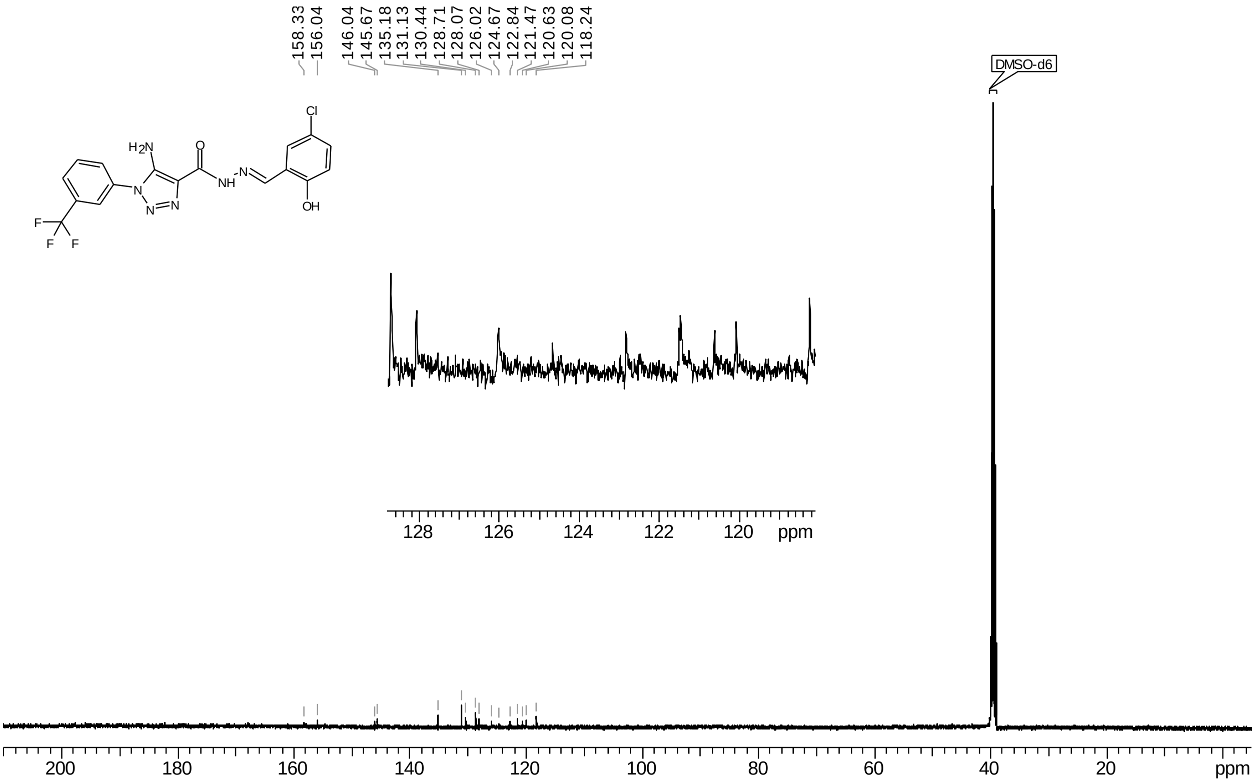

Figure S31 ^{13}C NMR spectrum of **E1**.

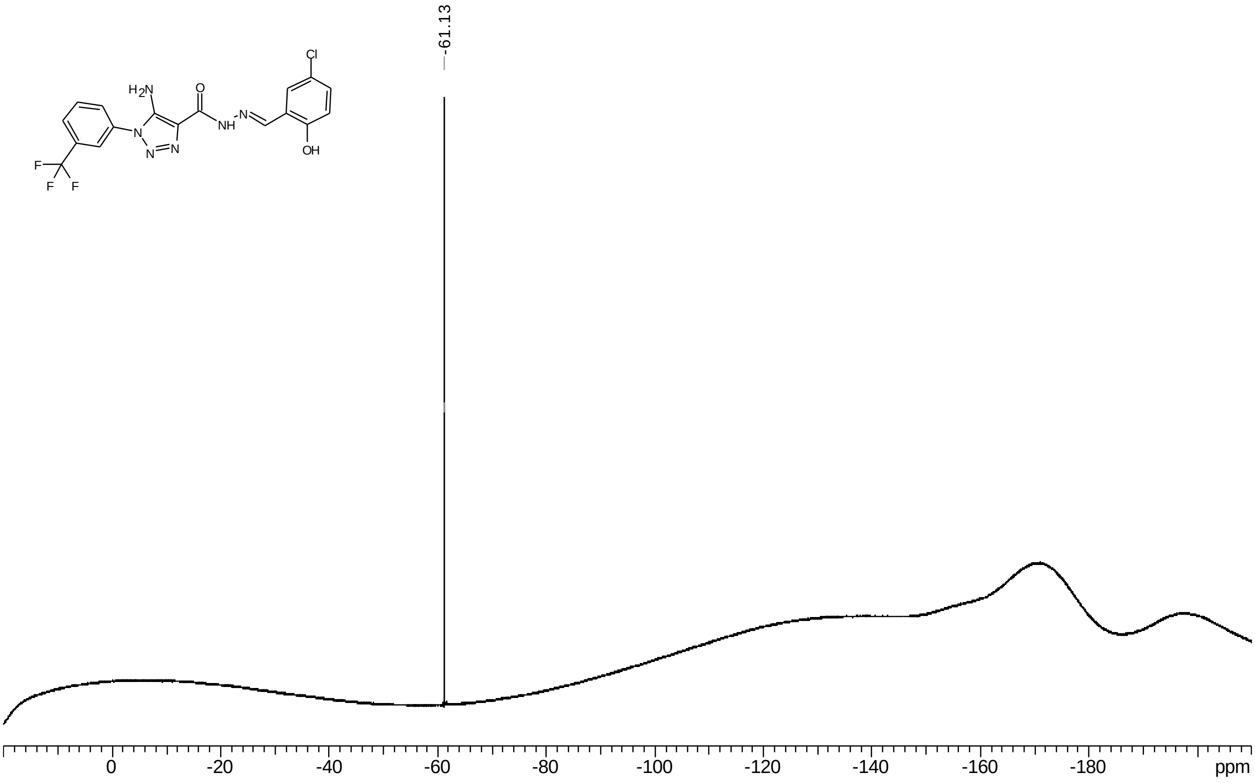

Figure S32. ^{19}F NMR spectrum of **E1**.

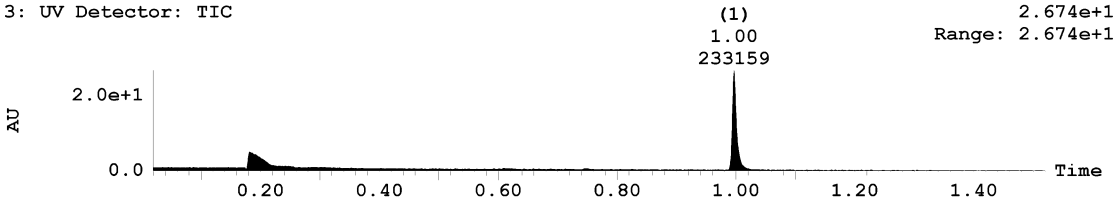

Peak Number	Time	Area %Total
1	1.00	100.00

Figure S33. LCMS purity analysis of **E1**.

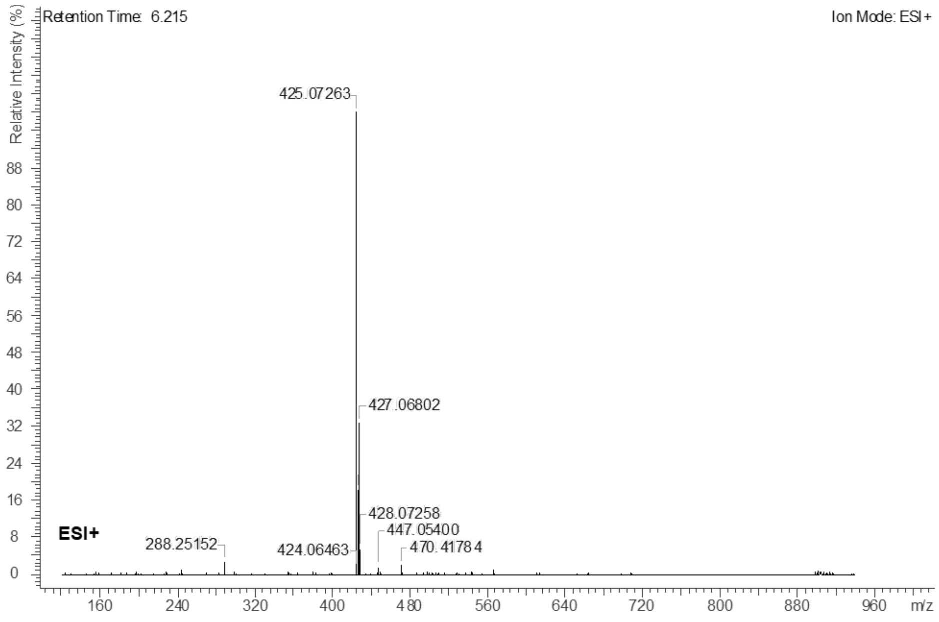

Figure S34 HRMS of **E1**.

Compound **E4**

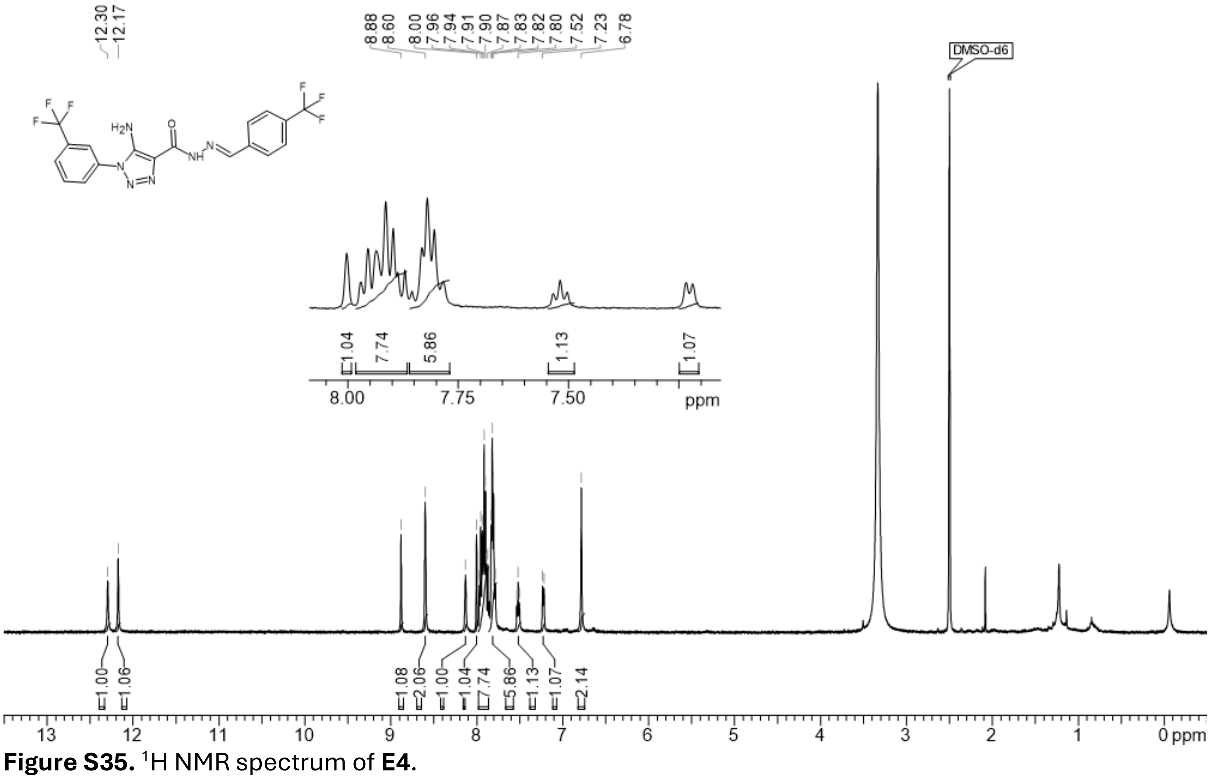

Figure S35. ^{1}H NMR spectrum of **E4**.

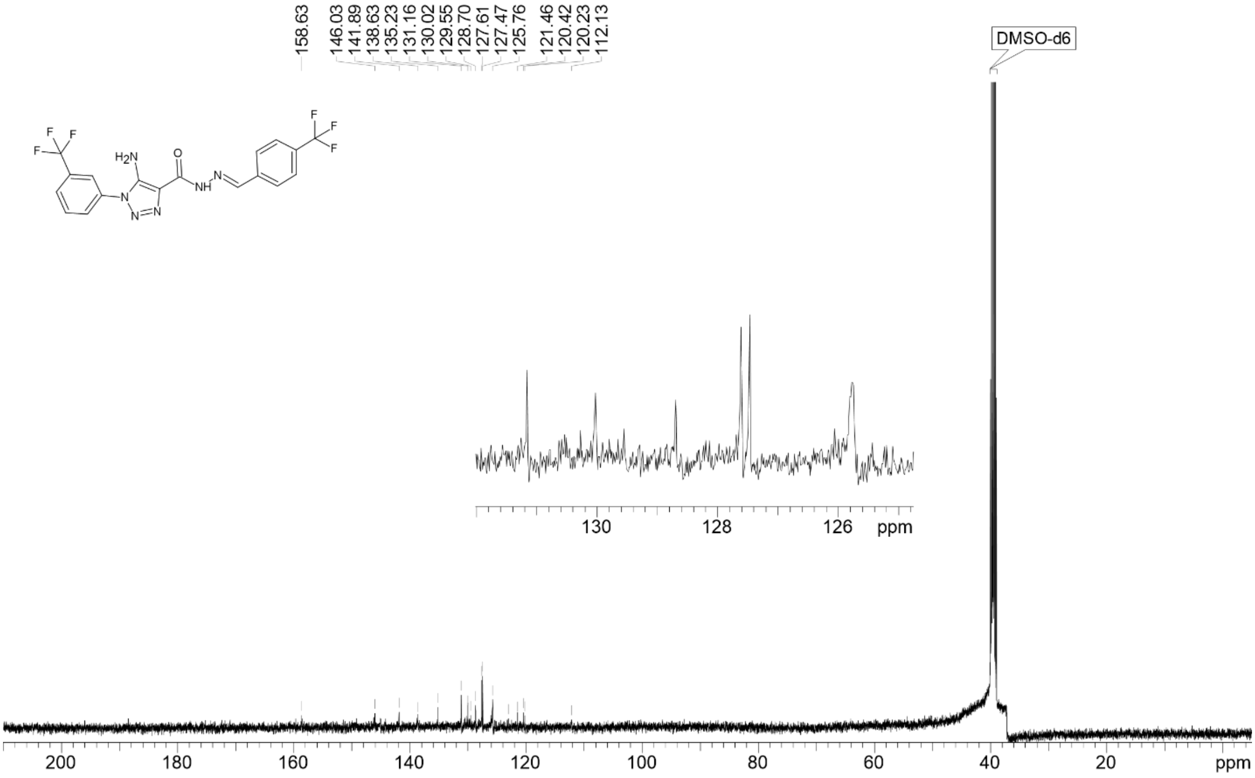

Figure S36. ^{13}C NMR spectrum of **E4**.

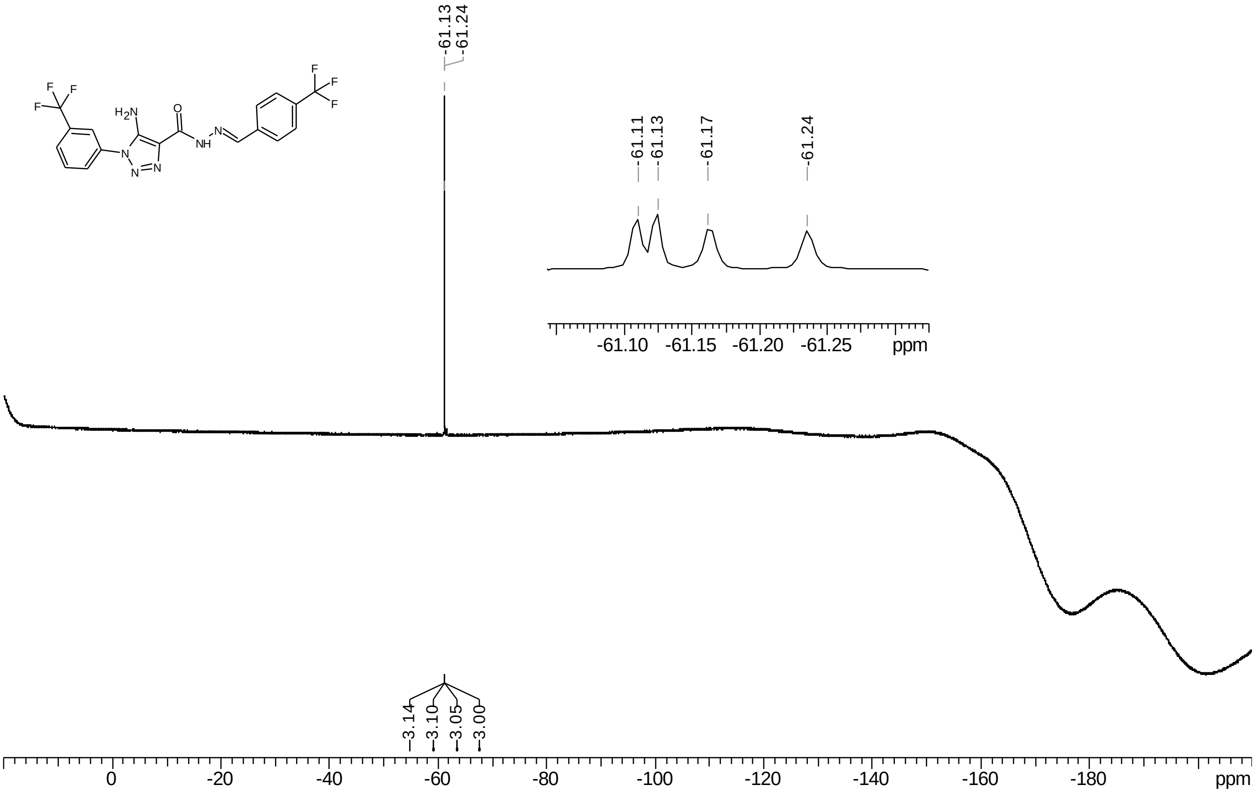

Figure S37. ^{19}F NMR spectrum of **E4**.

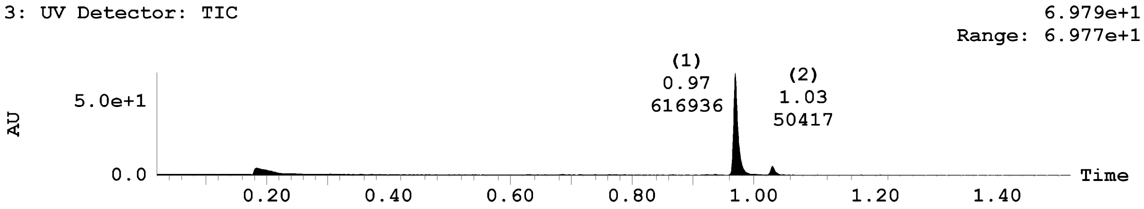

Peak Number	Time	Area %Total
1	0.97	92.45
2	1.03	7.55

Peak ID	Time	Mass Found	BPM
1	0.97	442.10	443.14

Peak ID	Time	Mass Found	BPM
2	1.03	442.10	443.14

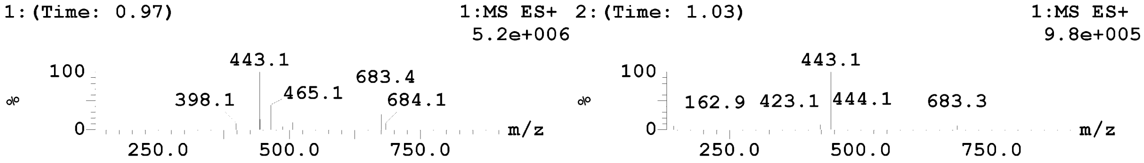

Peak ID	Time	Mass Found	BPM
1	0.97	442.10	441.28

Peak ID	Time	Mass Found	BPM
2	1.03	442.10	441.30

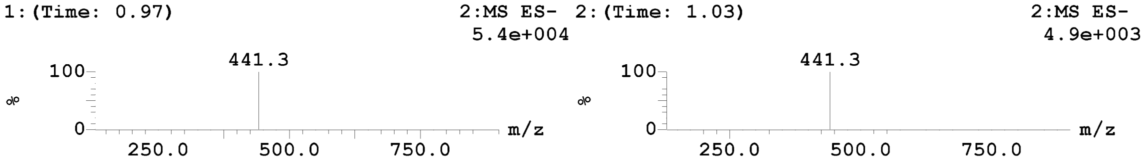

Figure S38. LCMS purity analysis of **E4**.

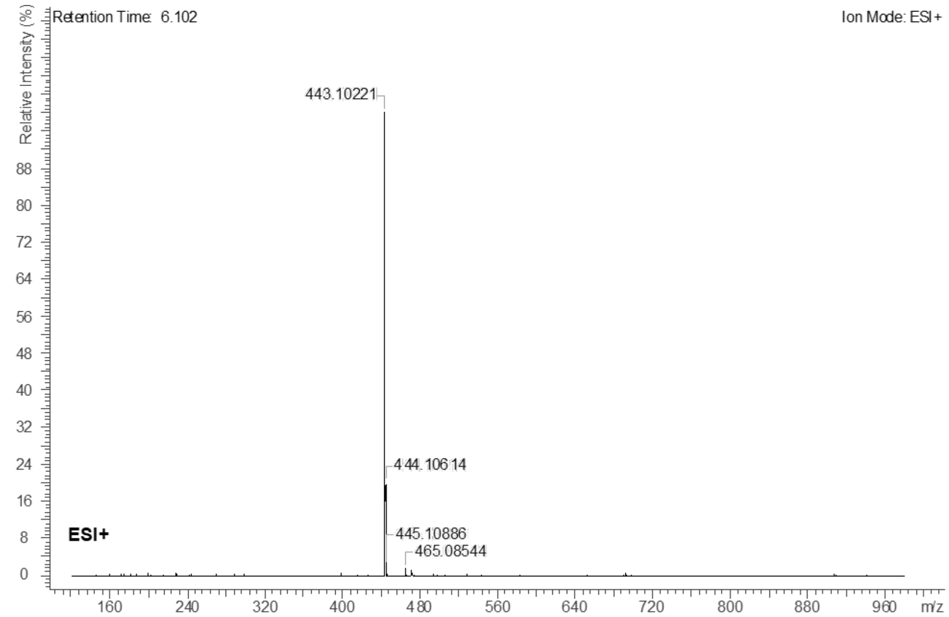

Figure S39. HRMS of **E4**.

Compound **E6**

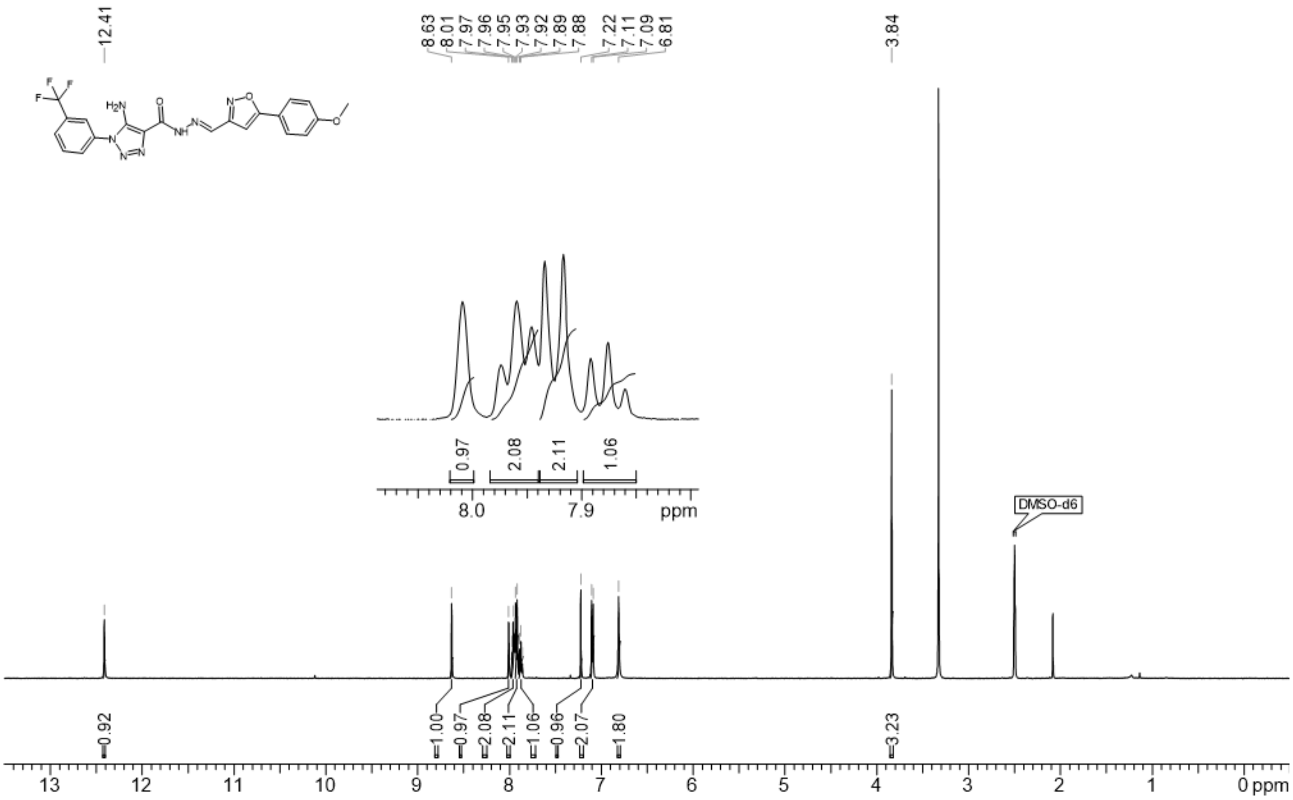

Figure S40. ^{1}H NMR spectrum of **E6**.

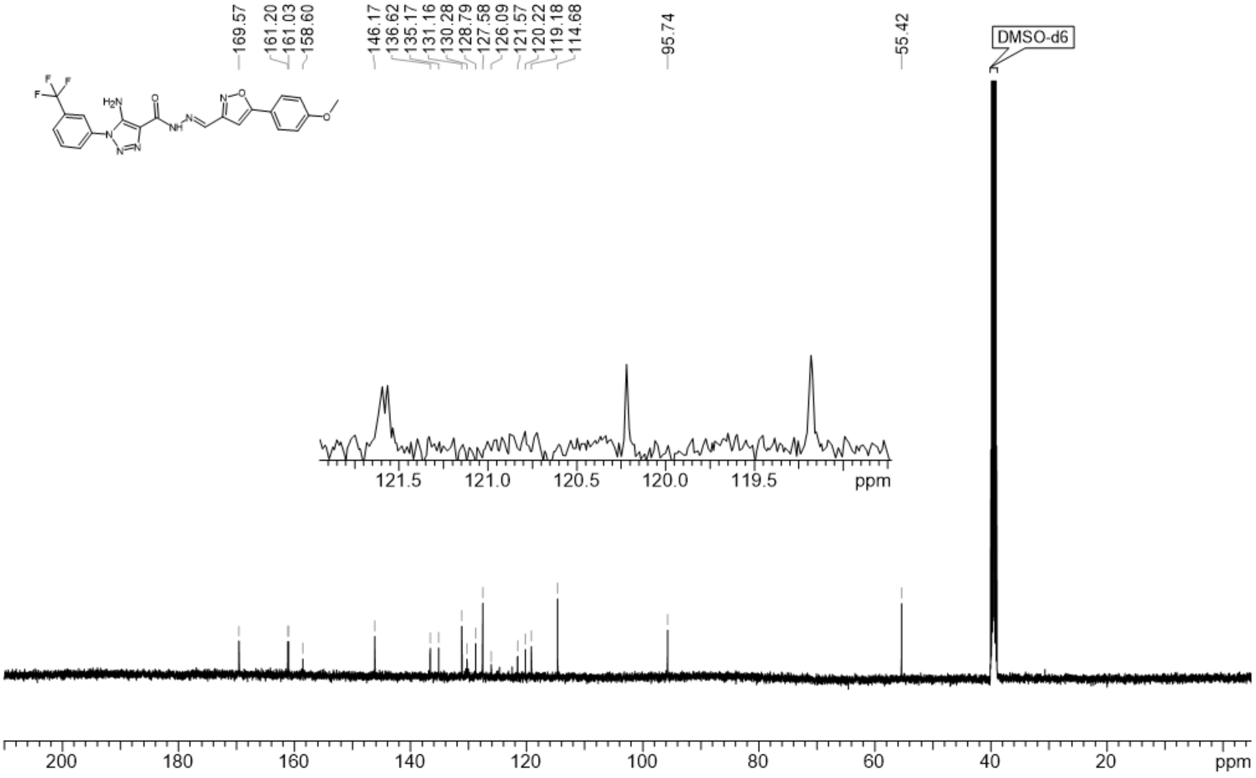

Figure S41. ^{13}C NMR spectrum of **E6**.

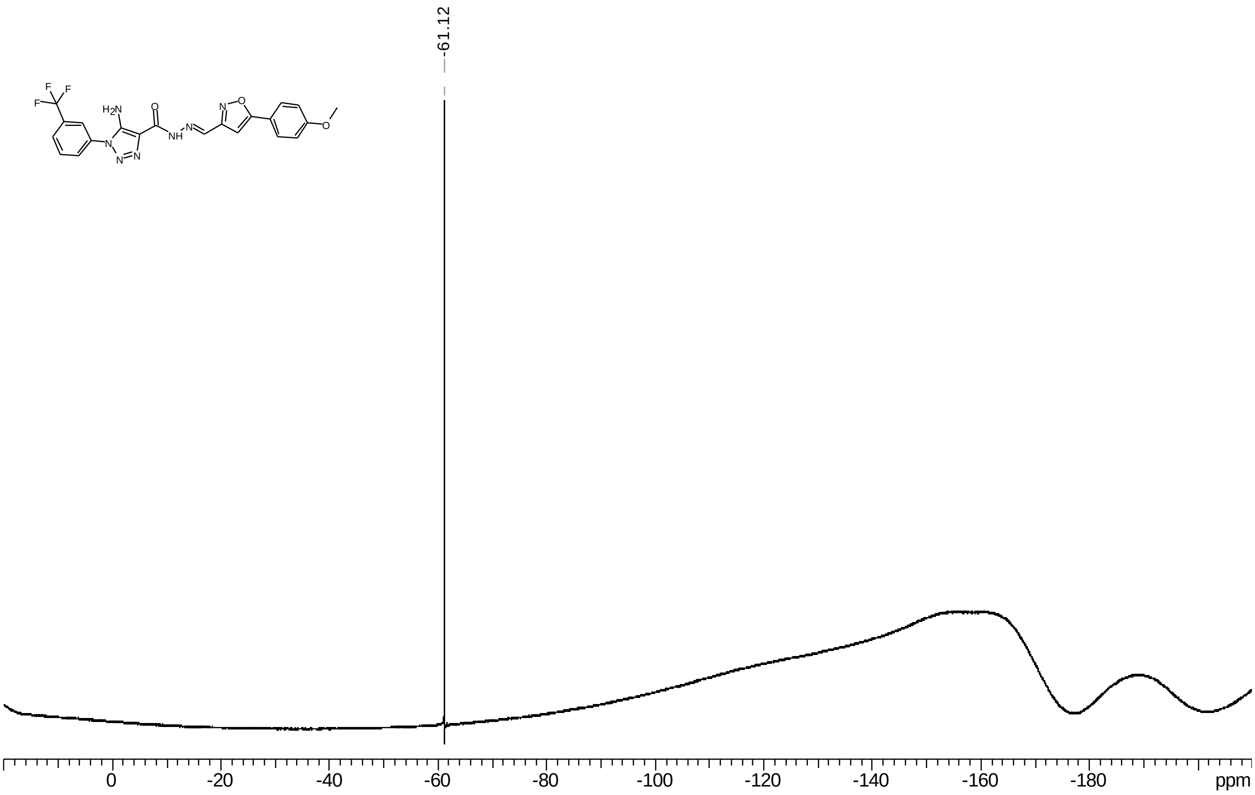

Figure S42. ^{19}F NMR spectrum of **E6**.

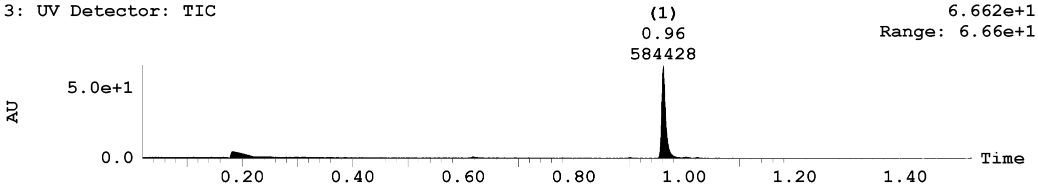

Peak Number	Time	Area %Total
1	0.96	100.00

Figure S43. LCMS purity analysis of **E6**.

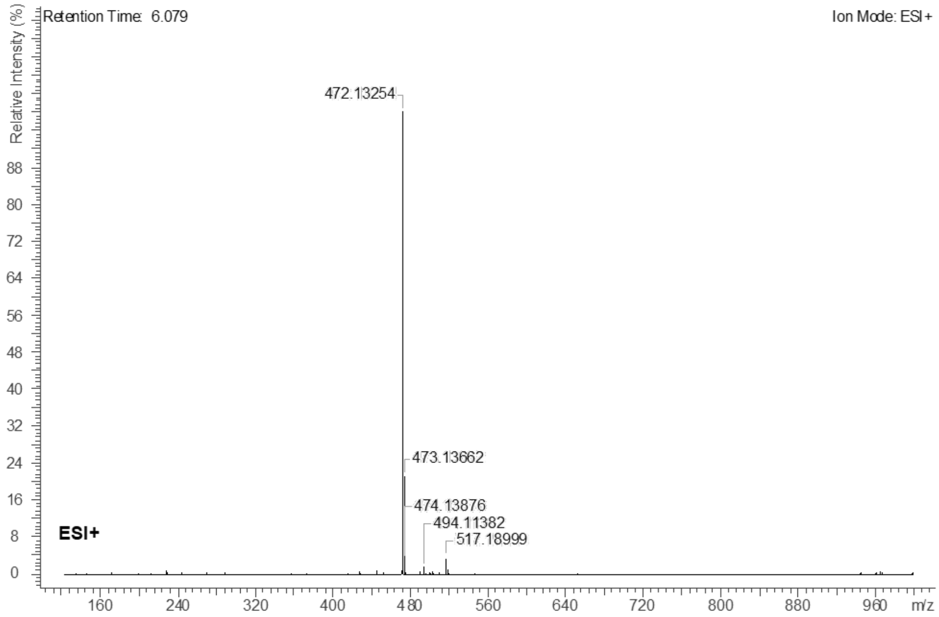

Figure S44. HRMS of **E6**.

Compound **H1**

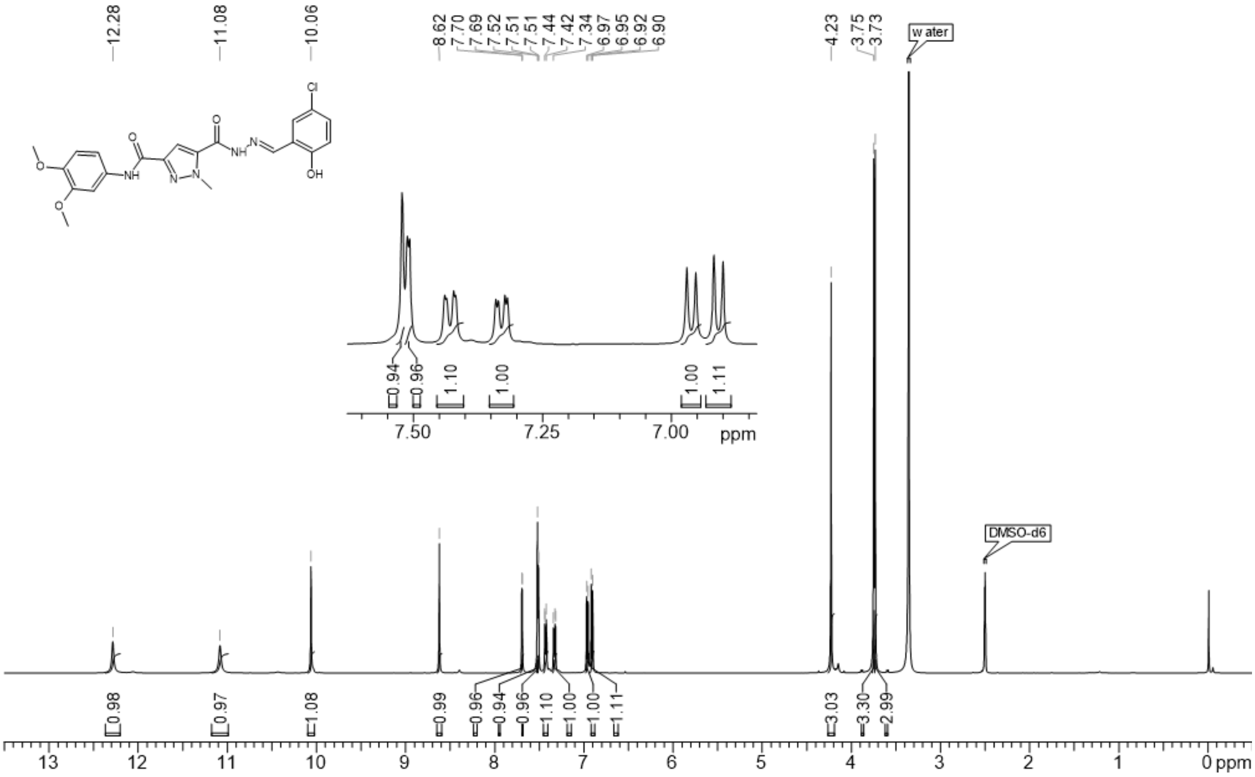

Figure S45. ^{1}H NMR spectrum of **H1**.

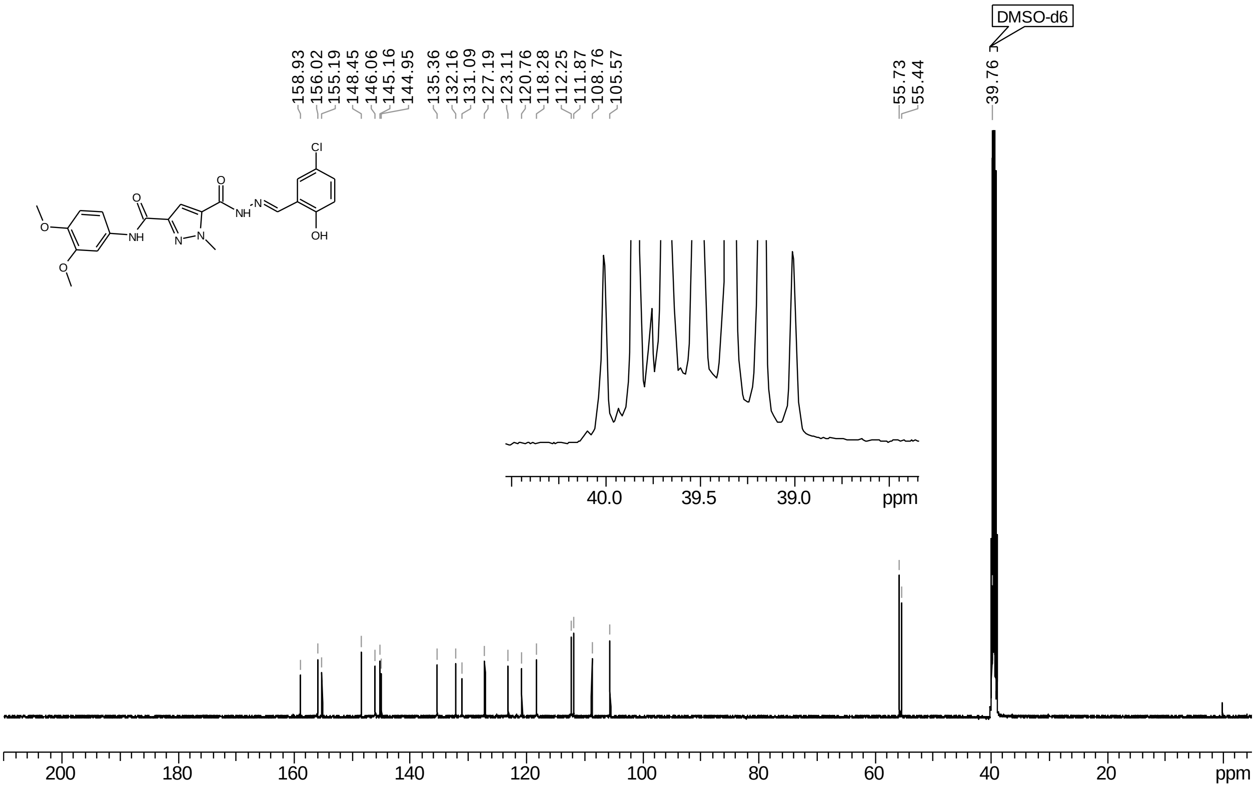

Figure S46. ^{13}C NMR spectrum of **H1**.

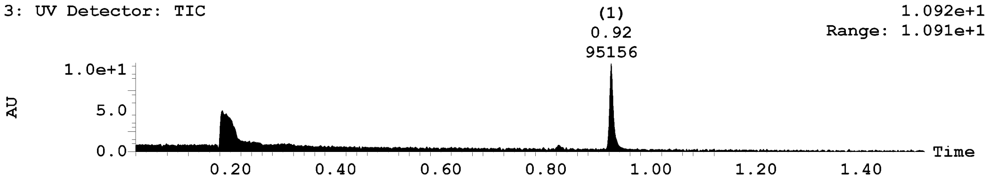

Peak Number	Time	Area %Total
1	0.92	100.00

Figure S47. LCMS purity analysis of **H1**.

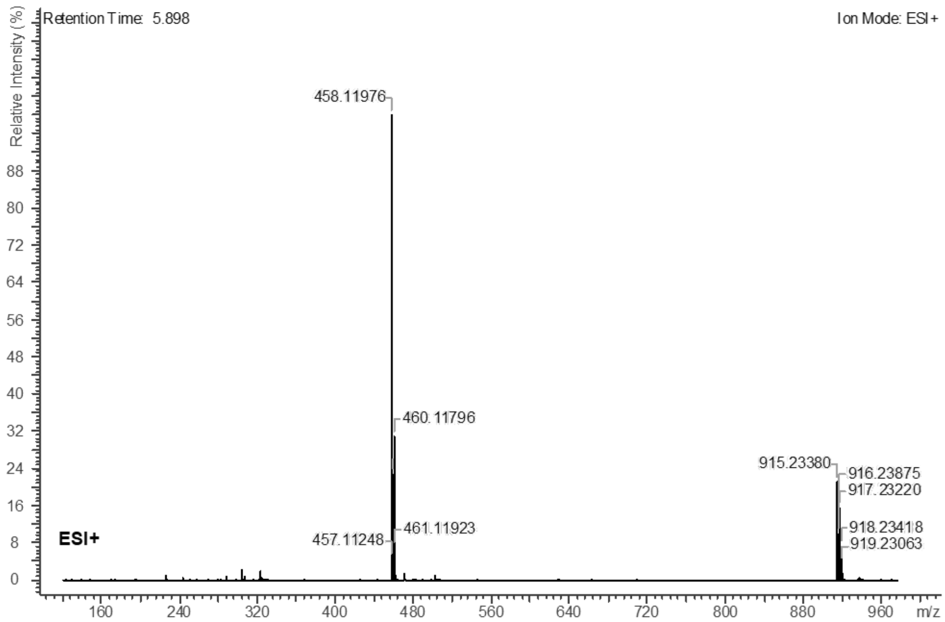

Figure S48. HRMS of **H1**.

Compound **I1**

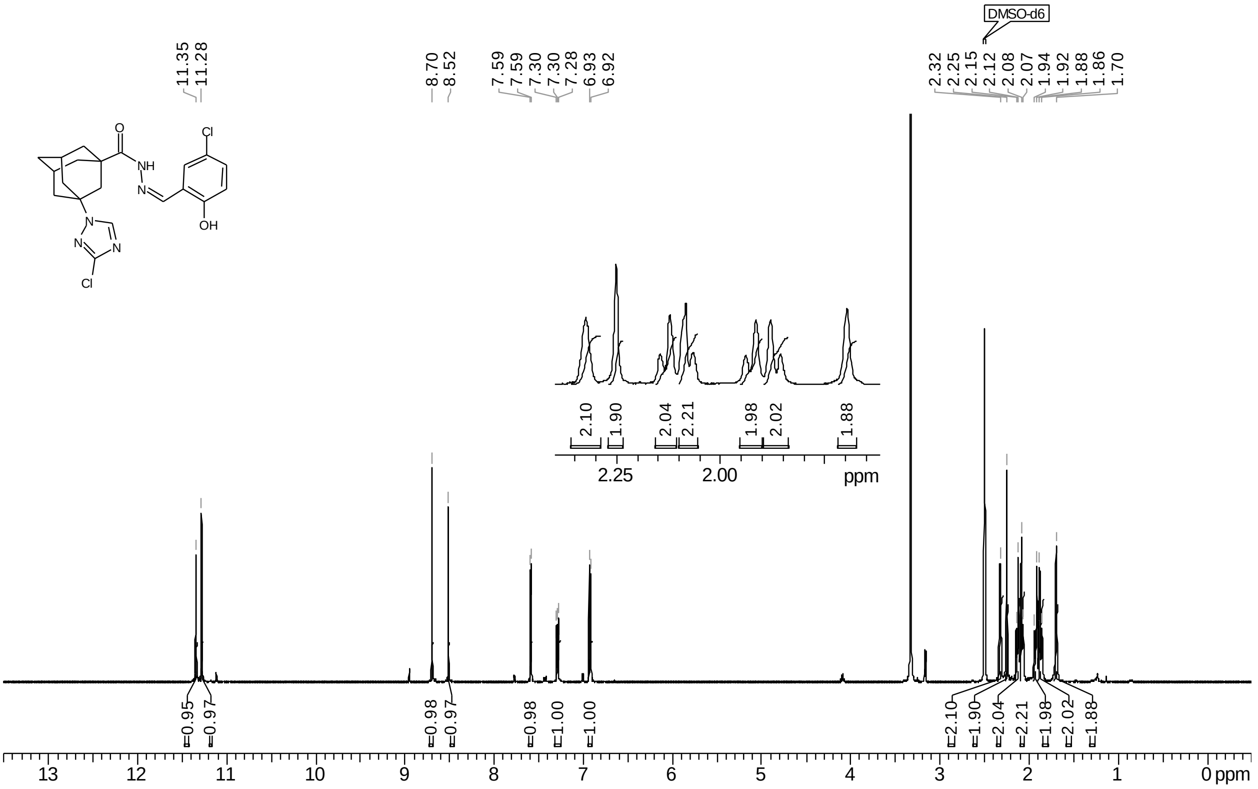

Figure S49. ^{1}H NMR spectrum of **I1**.

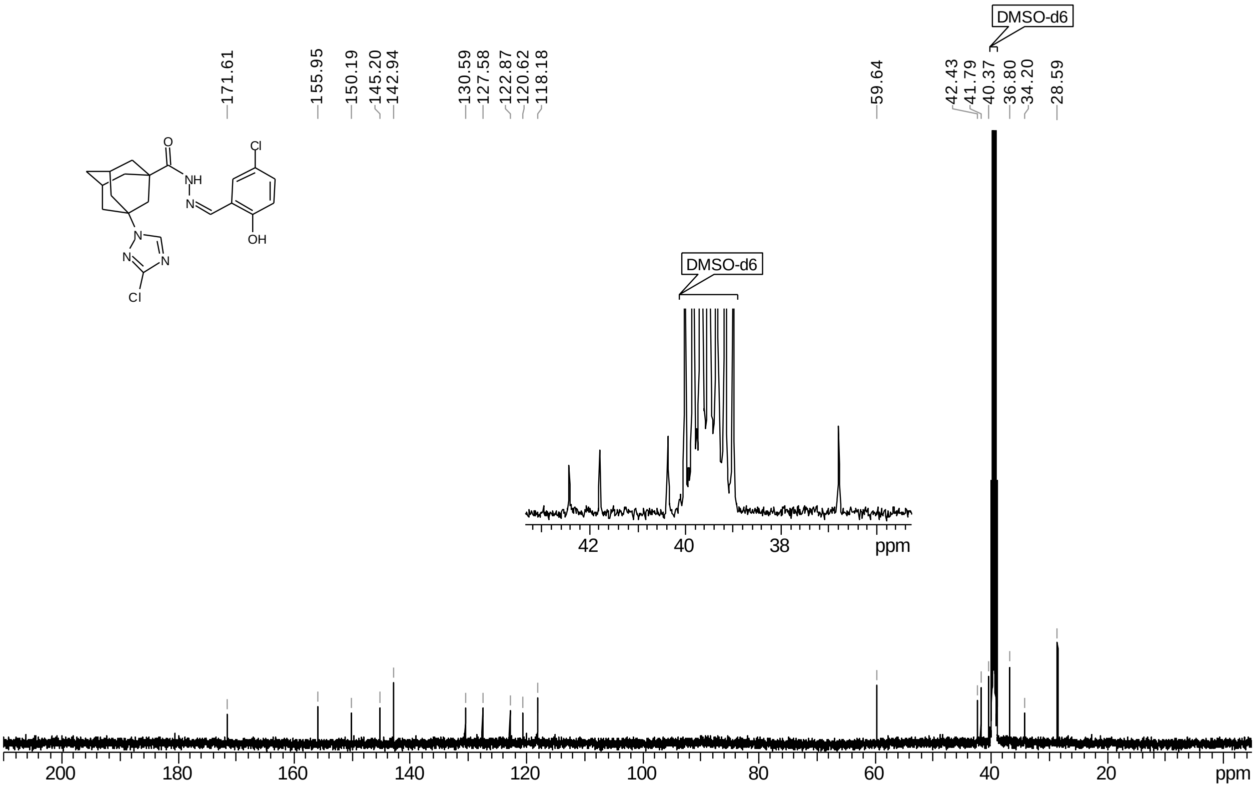

Figure S50. ^{13}C NMR spectrum of **I1**.

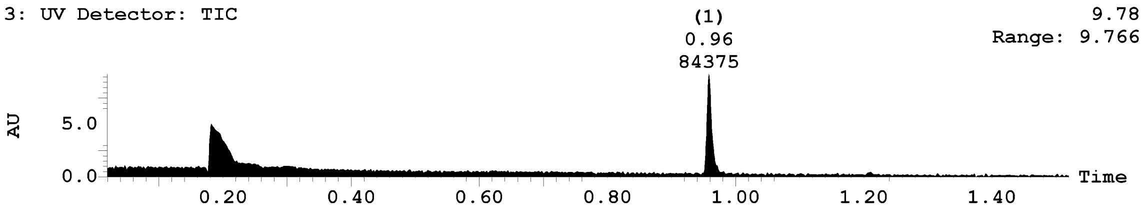

Peak Number	Time	Area %Total
1	0.96	100.00

Figure S51. LCMS purity analysis of **I1**.

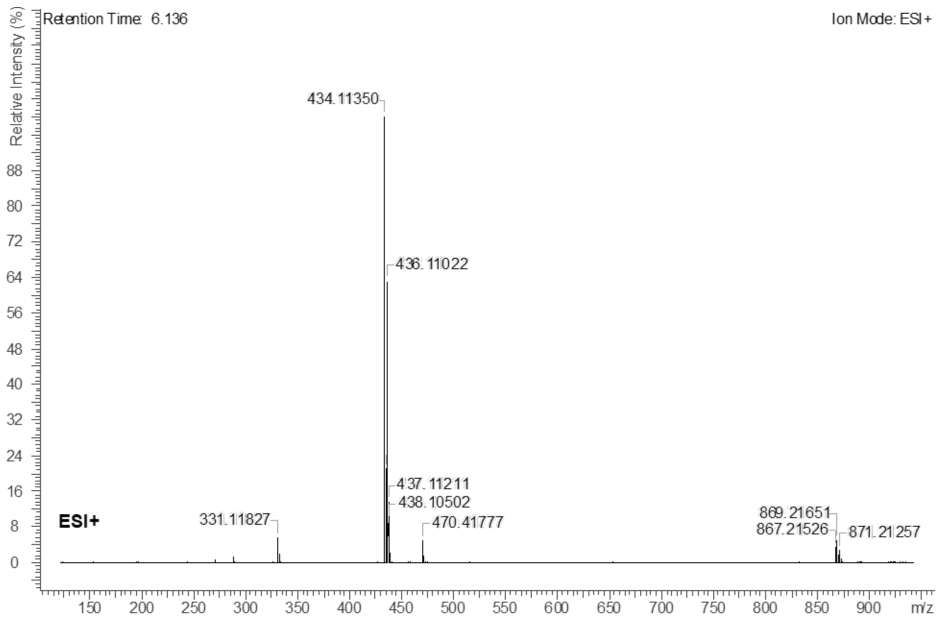

Figure S52. HRMS of **I1**.

Compound **K1**

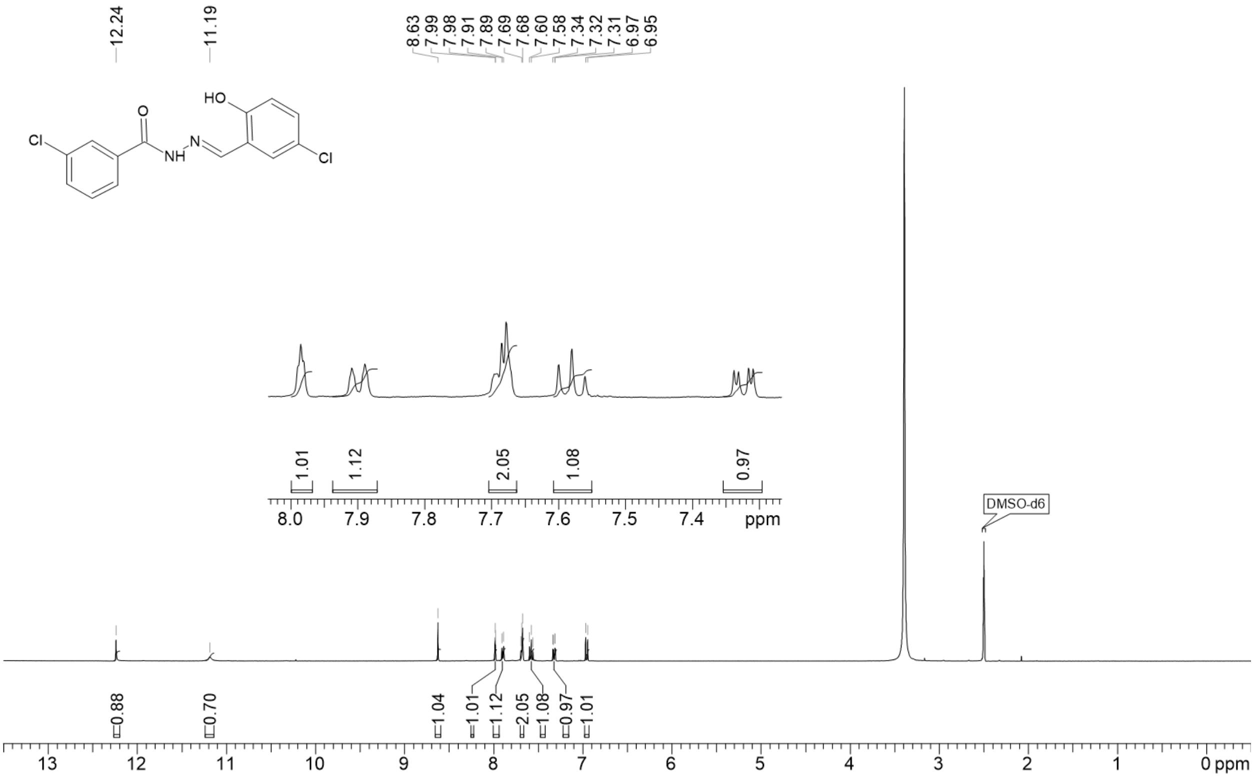

Figure S53. ^{1}H NMR spectrum of **K1**.

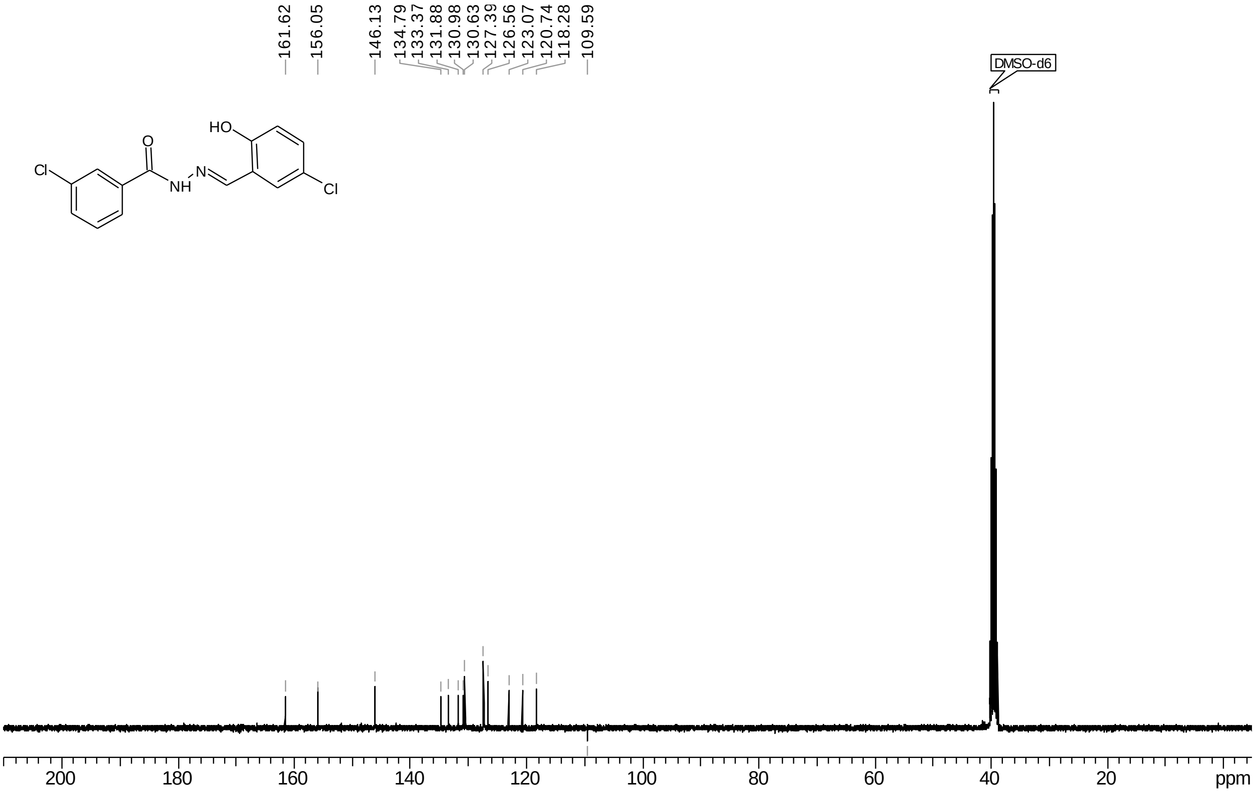

Figure S54. ^{13}C NMR spectrum of **K1**.

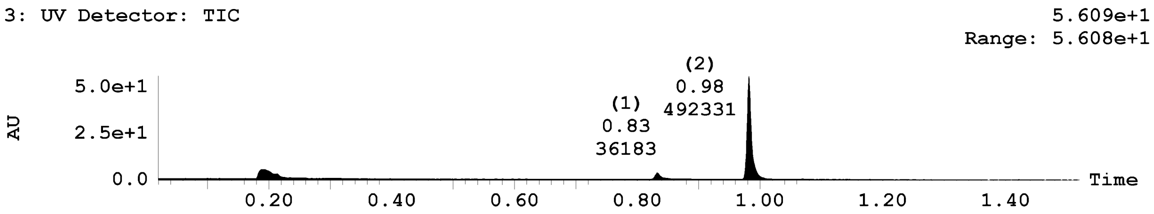

Peak Number	Time	Area %Total
1	0.83	6.85
2	0.98	93.15

Peak ID	Time	Mass Found	BPM
1	0.83	308.01	309.04

Peak ID	Time	Mass Found	BPM
2	0.98	308.01	309.01

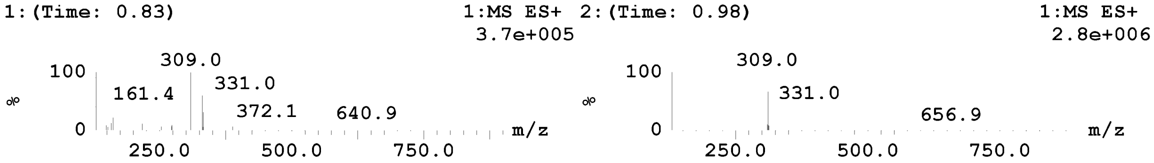

Figure S55. LCMS purity analysis of **K1**.

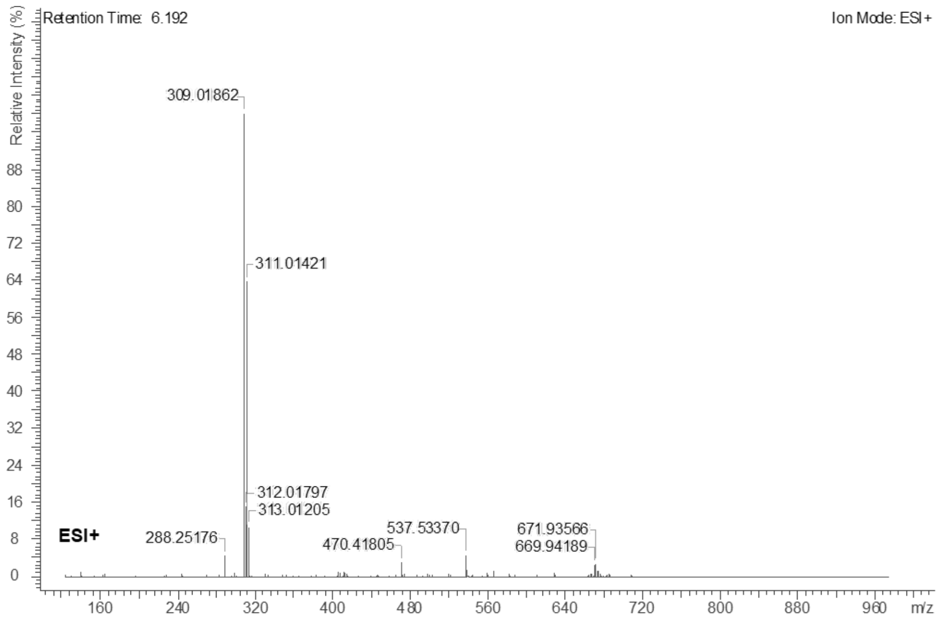

Figure S56. HRMS of **K1**.

Compound **K4**

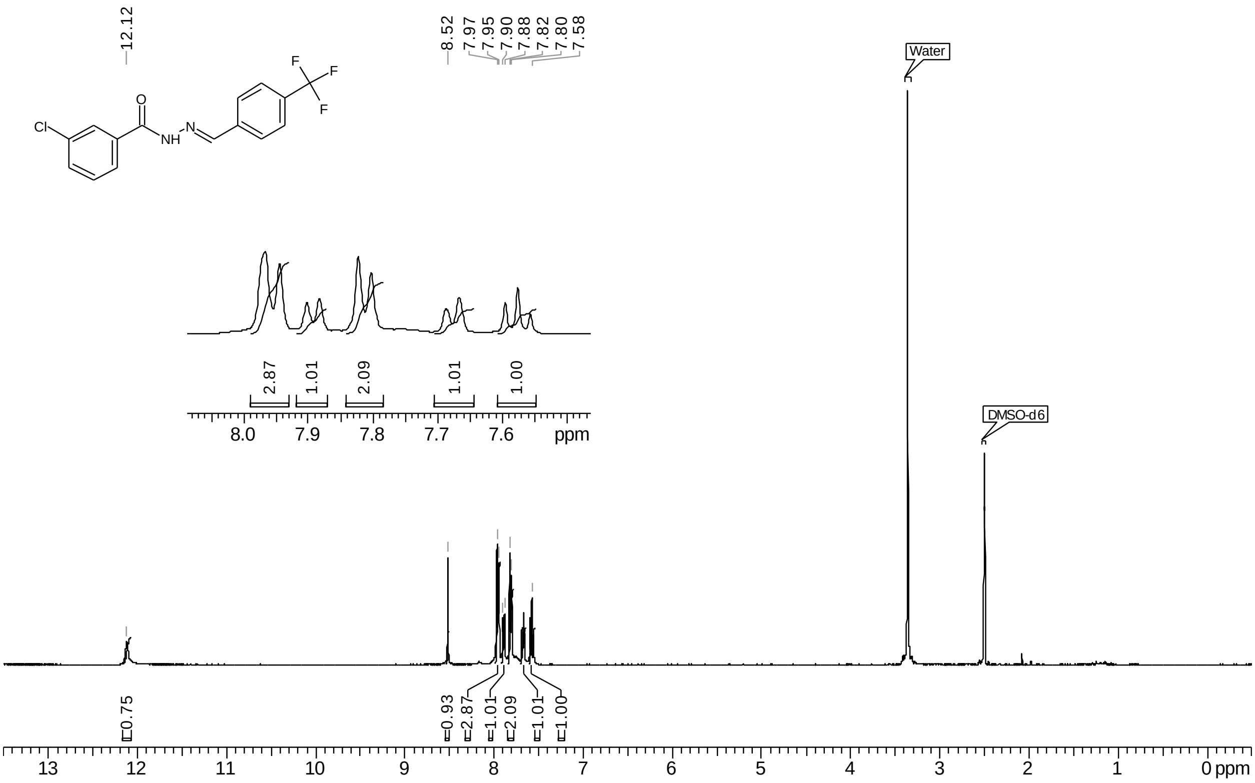

Figure S57. ^{1}H NMR spectrum of **K4**.

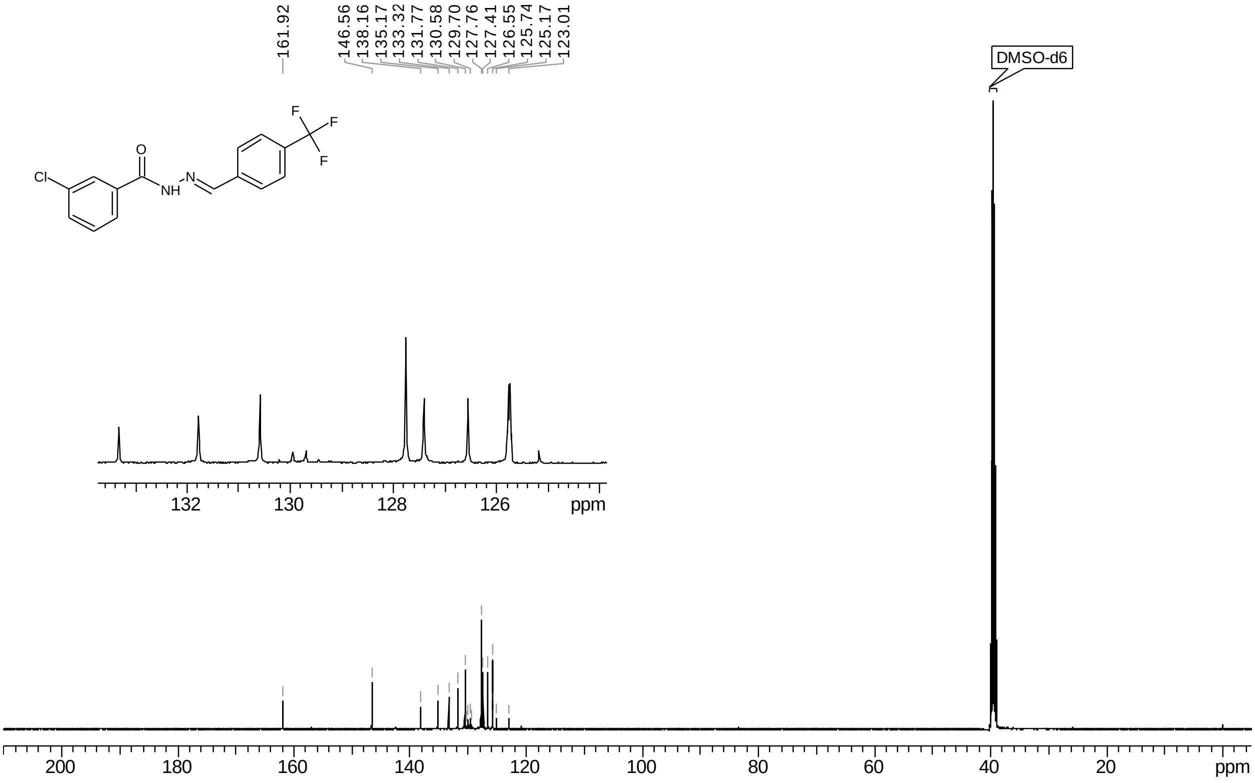

Figure S58. ^{13}C NMR spectrum of **K4**.

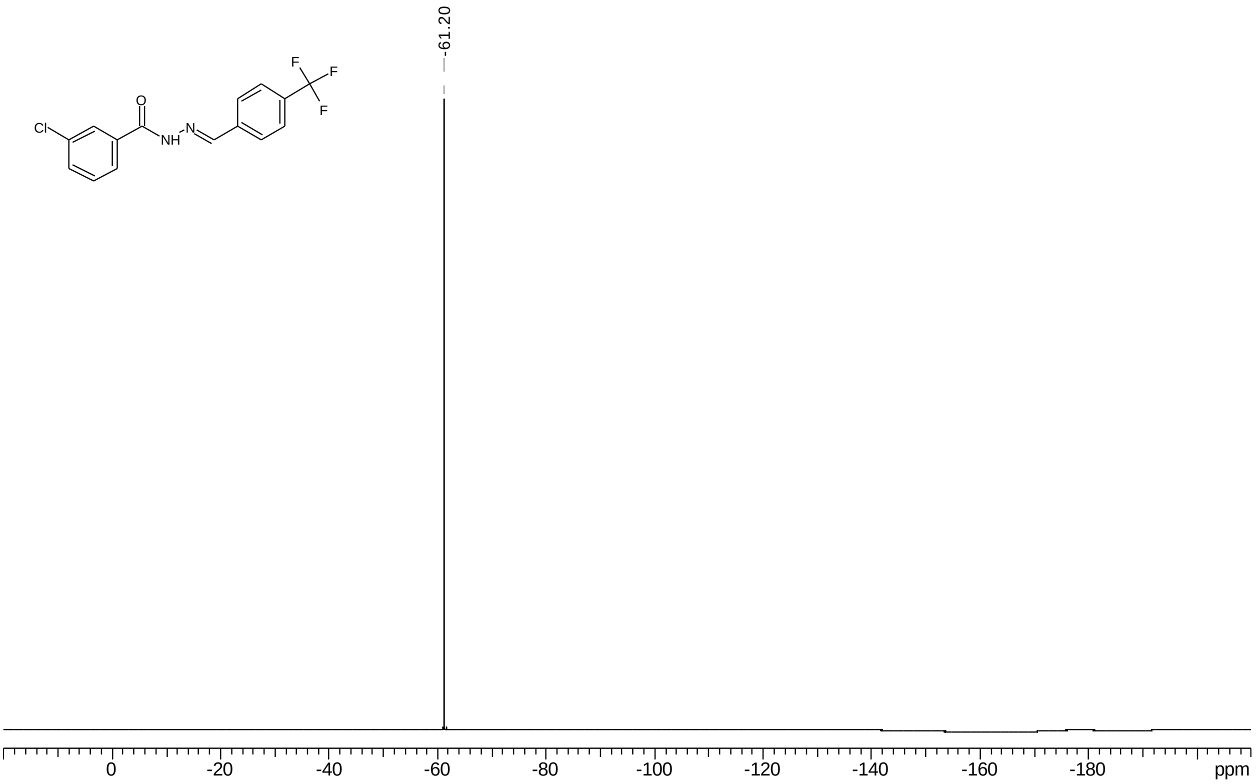

Figure S59. ^{19}F NMR spectrum of **K4**.

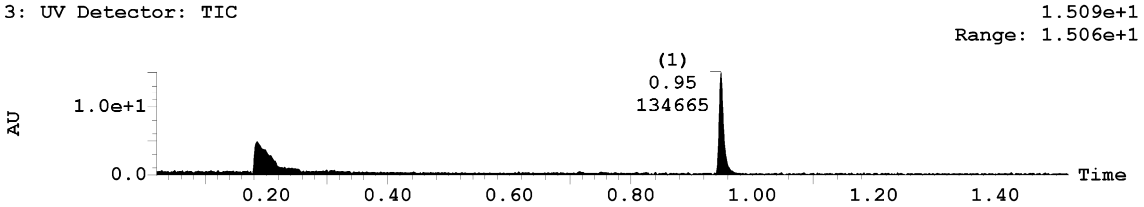

Peak Number	Time	Area %Total
1	0.95	100.00

Figure S60. LCMS purity analysis of **K4**.

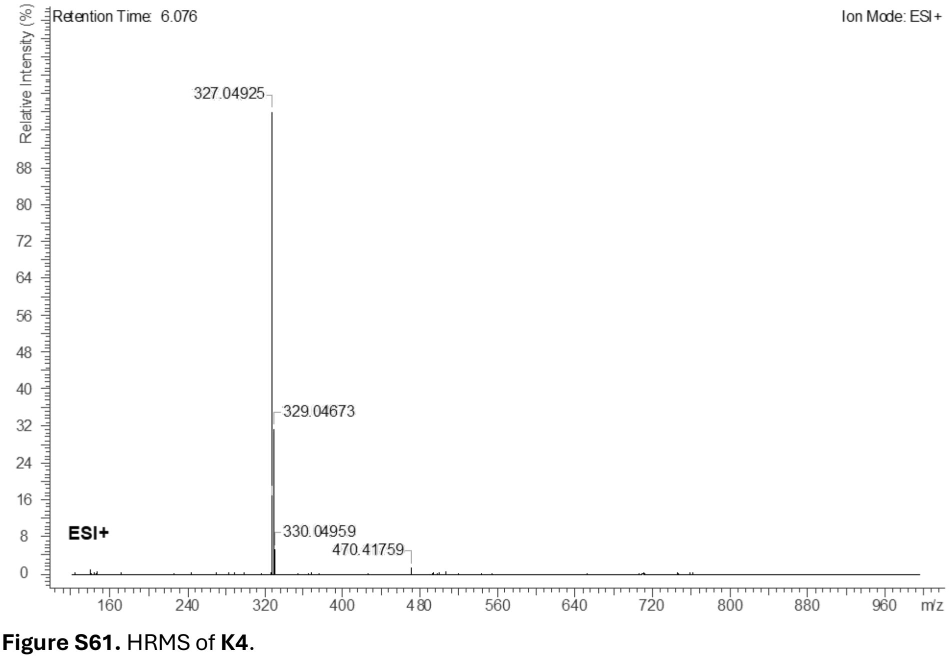

Figure S61. HRMS of **K4**.

Compound **L1**

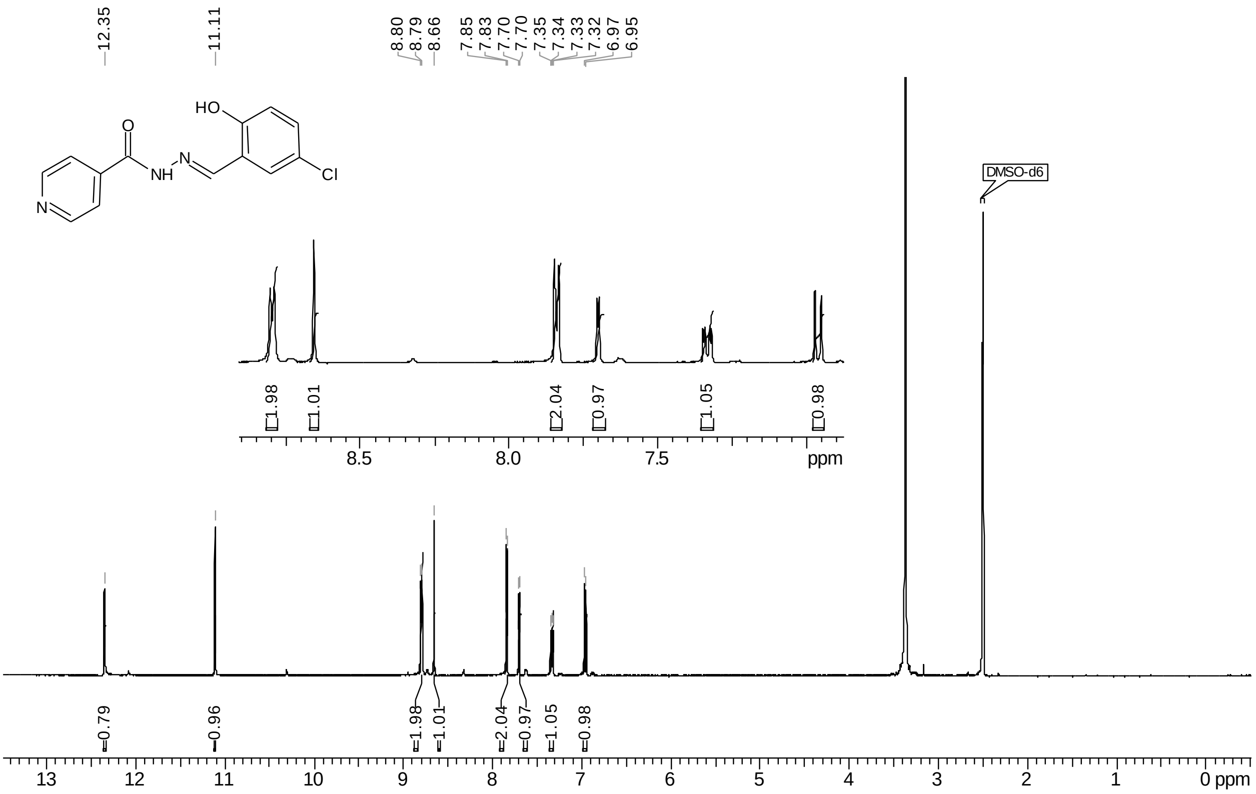

Figure S62. ^{1}H NMR spectrum of **L1**.

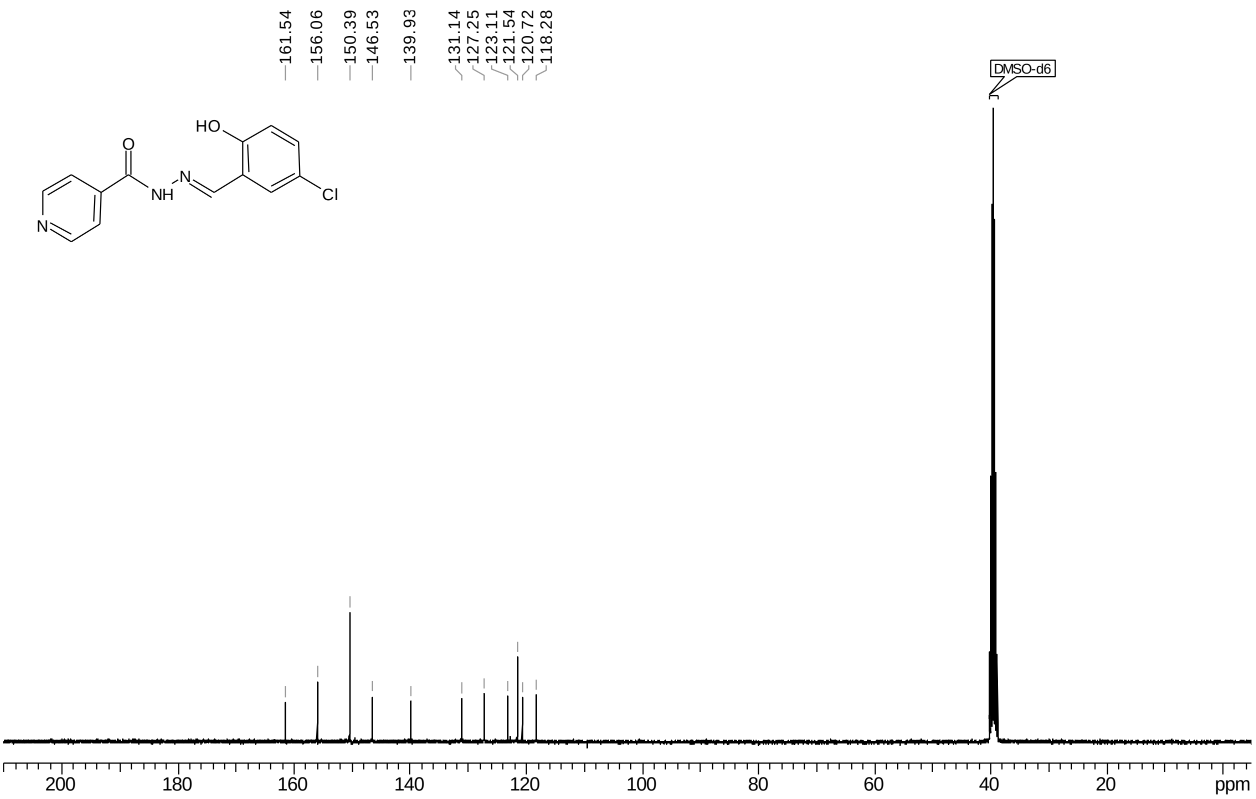

Figure S63. ^{13}C NMR spectrum of **L1**.

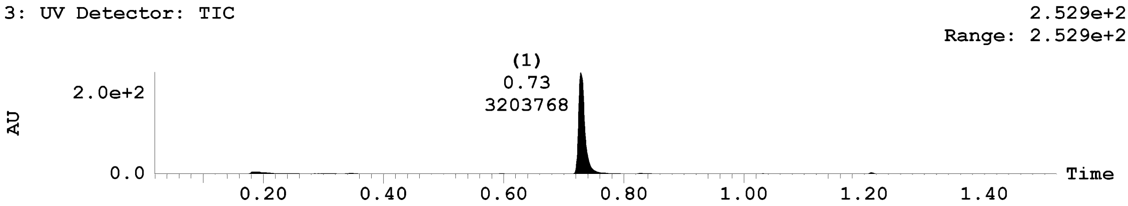

Peak Number	Time	Area %Total
1	0.73	100.00

Figure **S64.** LCMS purity analysis of **L1**.

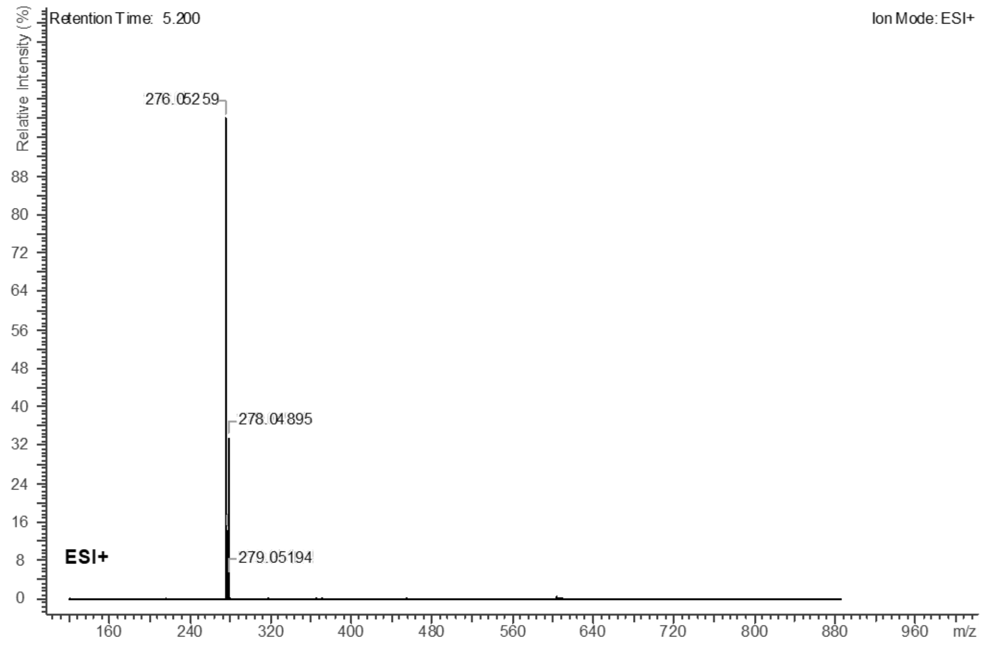

Figure **S65.** HRMS of **L1**.

Supplementary references

(1) Jumde, R. P.; Guardigni, M.; Gierse, R. M.; Alhayek, A.; Zhu, D.; Hamid, Z.; Johannsen, S.; Elgaher, W. A. M.; Neusens, P. J.; Nehls, C.; Haupenthal, J.; Reiling, N.; Hirsch, A. K. H. Hit-Optimization Using Target-Directed Dynamic Combinatorial Chemistry: Development of Inhibitors of the Anti-Infective Target 1-Deoxy-D-Xylulose-5-Phosphate Synthase. *Chem Sci* **2021**, *12* (22), 7775–7785. https://doi.org/10.1039/D1SC00330E.

(2) Hartman, A. M.; Gierse, R. M.; Hirsch, A. K. H. Protein-Templated Dynamic Combinatorial Chemistry: Brief Overview and Experimental Protocol. *European J Org Chem* **2019**, *22*, 3581–3590. https://doi.org/10.1002/ejoc.201900327.

(3) Kolbe, K.; Möckl, L.; Sohst, V.; Brandenburg, J.; Engel, R.; Malm, S.; Bräuchle, C.; Holst, O.; Lindhorst, T. K.; Reiling, N. Azido Pentoses: A New Tool To Efficiently Label *Mycobacterium Tuberculosis* Clinical Isolates. *ChemBioChem* **2017**, *18* (13), 1172–1176. https://doi.org/10.1002/cbic.201600706.

(4) Zelmer, A.; Carroll, P.; Andreu, N.; Hagens, K.; Mahlo, J.; Redinger, N.; Robertson, B. D.; Wiles, S.; Ward, T. H.; Parish, T.; Ripoll, J.; Bancroft, G. J.; Schaible, U. E. A New in Vivo Model to Test Anti-Tuberculosis Drugs Using Fluorescence Imagin8g. *J. Antimicrob. Chemother.* **2012**, *67* (8), 1948–1960. https://doi.org/10.1093/jac/dks161.

(5) Hirsch, A. K. H.; Alphey, M. S.; Lauw, S.; Seet, M.; Barandun, L.; Eisenreich, W.; Rohdich, F.; Hunter, W. N.; Bacher, A.; Diederich, F. Inhibitors of the Kinase IspE: Structure-Activity Relationships and Co-Crystal Structure Analysis. *Org Biomol Chem* **2008**, *6* (15), 2719–2730. https://doi.org/10.1039/b804375b.

(6) Ropponen, H. K.; Diamanti, E.; Siemens, A.; Illarionov, B.; Haupenthal, J.; Fischer, M.; Rottmann, M.; Witschel, M.; Hirsch, A. K. H. Assessment of the Rules Related to Gaining Activity against Gram-Negative Bacteria. *RSC Med Chem* **2021**, *12* (4), 593–601. https://doi.org/10.1039/d0md00409j.

(7) Ropponen, H.; Diamanti, E.; Johannsen, S.; Illarionov, B.; Hamid, R.; Jaki, M.; Sass, P.; Fischer, M.; Haupenthal, J.; Hirsch, A. K. H. Exploring the Translational Gap of a Novel Class of *Escherichia Coli* IspE Inhibitors. *ChemMedChem* **2023**, *18* (19). https://doi.org/10.1002/cmdc.202300346.

(8) Choi, S.; Narayanasamy, P. Investigating Novel IspE Inhibitors of the MEP Pathway in Mycobacterium. *Microorganism* **2024**, *12* (18). https://doi.org/https://doi.org/10.3390/microorganisms12010018.
